# The Latest Achievements in the Design of Permanent Fillings for Conservative Dentistry Based on Indenoquinoxaline Derivatives as Photoinitiators of Visible-Light Polymerization: Mass and Colour Stability

**DOI:** 10.3390/ijms26115424

**Published:** 2025-06-05

**Authors:** Ilona Pyszka, Oliwia Szczepańska, Beata Jędrzejewska

**Affiliations:** Faculty of Chemical Technology and Engineering, Bydgoszcz University of Science and Technology, 85-326 Bydgoszcz, Polandbeata@pbs.edu.pl (B.J.)

**Keywords:** photopolymerization, photocurable composition, indenoquinoxaline derivatives, binary photoinitiating systems, mass, colour stability

## Abstract

The demand for polymer composite materials in the dental market is increasing every year. This rise is due to their excellent properties and ongoing technological advancements. The goal of this study was to develop new photoinitiators included in the liquid organic matrix, which is one of the main components of dental composites. Therefore, a series of compounds based on the indenoquinoxaline skeleton was synthesized, differing in the substituent. The spectroscopic properties of these compounds allowed their use as visible-light photoinitiators of radical polymerization in combination with (phenylthio)acetic acid. In addition to the polymerization kinetics, the lifetime and quantum yield of the triplet-state formation and the rate constants of its quenching by (phenylthio)acetic acid were determined. The durability of the designed composites was also assessed. Ageing tests included hydrothermal ageing, allowing for the determination of sorption, solubility, and mass change. Solutions imitating the oral cavity environment—distilled water, artificial saliva, *n*-heptane, and 3% acetic acid—as well as solutions containing pigments were used for these studies. Determination of the mass change and colour stability allowed for the assessment of how these materials react to long-term exposure in the oral environment. It was found that the solution simulating the natural oral environment has a significant impact on the hydrolytic stability and colour stability of the materials.

## 1. Introduction

Composite materials play a key role in modern dentistry. They are commonly used for fillings, tooth reconstruction, and aesthetic prosthetic restorations. They cover a remarkably diverse group of restorative materials in terms of properties and function. These differences result, among others, from the composition of the multi-component materials [1,2]. Polymer matrix composites consist of an organic phase (liquid polymer matrix) and an inorganic phase (filler). It may also contain auxiliary additives, such as polymerization inhibitors, colour stabilizers, dyes, and others. Each of these components affects the final properties of the resulting composite in some way [1].

When developing new dental composites, attention should be paid primarily to the appropriate selection of the polymer matrix composition and the type of filler. Most commercially available dental materials are based on acrylic resins, such as Bis-GMA (bisphenol A–glycidyl methacrylate), UDMA (urethane dimethacrylate) and TEGDMA (triethylene glycol dimethacrylate) used to dilute the composite [3,4,5]. The inorganic filler is usually silica, borosilicate glass, zirconium, or aluminosilicate particles. Its main role is to improve mechanical properties, abrasion, and aesthetics [4,6].

The appropriate selection of the type and quantity of individual components has a decisive influence on the quality of the final product. It should also be added that the oral environment in which the polymer composite is placed is very complex and can affect the clinical properties of dental materials [7,8,9]. The use of any material, especially in a diverse environment, is associated with the occurrence of irreversible changes in the original properties of composites. This results in a reduction in its durability. Such deterioration of the composite properties over time is referred to as aging [10]. Therefore, the durability of all composite materials based on a polymer matrix is subject to aging processes and is mainly dependent on the environment in which they are used.

The consequence of the degradation processes of dental material occurring in the oral cavity environment may be the partial loss of hard tooth tissues resulting from the need to replace the filling [11,12,13,14,15,16,17,18,19,20,21,22].

One of the most commonly used commercial photoinitiators in dental composites is camphorquinone (CQ), often combined with amine co-initiators such as ethyl 4-dimethylaminobenzoate (EDMAB) to accelerate curing [23,24,25,26]. CQ-based systems absorb light mainly in the blue region, typical of dental lamp emission. However, they have some limitations due to the low molar absorption coefficient, yellowing of the cured material, and incomplete polymerization in deeper layers. These drawbacks may affect the aesthetic quality as well as the long-term durability of restorative materials.

The development of alternative photoinitiators aims to overcome the above-mentioned limitations, in particular, to improve the absorption properties of the photoinitiator, to increase the photochemical efficiency of the initiating system, and to improve the stability of the composites.

Therefore, the primary goal of our research described in this paper was to design new two-component photocurable systems based on indenoquinoxaline derivatives and (phenylthio)acetic acid (PhTAA), which are part of a liquid organic matrix, and their application in dental composites. Spectroscopic properties of the synthesized photoinitiators, such as the absorption band position and molar absorption coefficient, the lifetime and quantum yield of the triplet-state formation, and the rate constants of quenching of the excited triplet state of the tested compounds by (phenylthio)acetic acid, as well as the polymerization rates, were determined. In the next stage, the tested photoinitiating systems were applied to develop dental composites used in regenerative dentistry. The obtained composites were subjected to aging processes to determine their hydrolytic stability and colour stability. The results were compared with commercial CQ-based systems.

## 2. Results

### 2.1. Spectroscopic Properties

**Figure 1:**
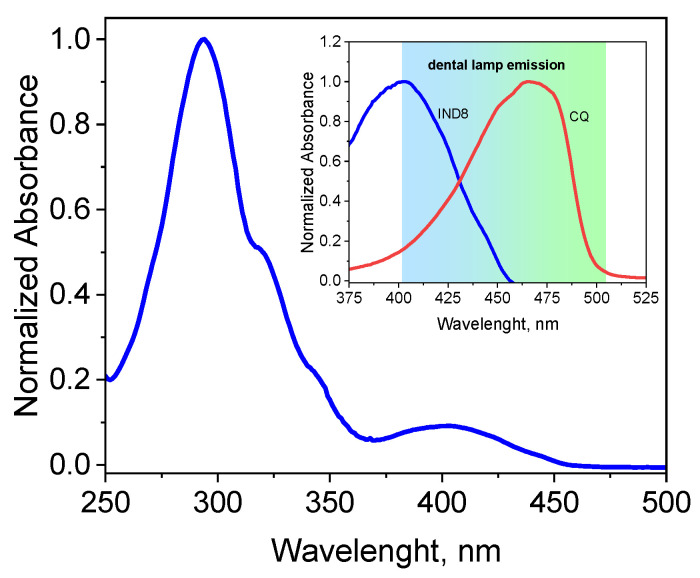
Normalized electronic absorption spectrum of IND8 in ethyl acetate. Inset: Normalized electronic absorption spectrum of IND8 and CQ in ethyl acetate. Blue–green colour indicates the light emission region of the Cromalux 75 lamp.

An example of the electronic absorption spectrum of the indenoquinoxaline derivative IND8 in ethyl acetate is shown in Figure 1. The spectra of the remaining synthesized compounds (IND1-IND7, IND9-IND10) are given in the Supplementary Materials (Figure S1).

Based on the electronic absorption spectra, the wavelength at which the absorption maximum occurs (λ_max_) and the molar absorption coefficients (ε) were determined (Table 1).

Figure 1 compares the absorption spectra of the obtained indenoquinoxaline IND8 with the absorption spectrum of camphorquinone. The commercial compound absorbs radiation in the visible region in the range of 400–500 nm [27], which means it is widely used as an initiator of photocurable dental materials [28,29]. However, the disadvantage of compounds absorbing light in this region is their intense yellow colour. This in turn affects the quality of the final product and consequently the aesthetics of the reconstruction of hard dental tissues. The presence of a conjugated double-bond system in the synthesized indenoquinoxaline molecules (IND1-IND10) enables the absorption of electromagnetic radiation emitted by dental lamps, which is the basis for the use of these dyes as photoinitiators of free radical polymerization for dental applications.

The electronic absorption spectra of the synthesized indenoquinoxalines are characterized by a typical shape for heterocyclic aromatic compounds with fused rings. They consist of two clearly separated bands—one in the blue light range and the other in the green light range (Figure 1 and Figure S1). These absorption bands correspond to the S_0_-S_2_ and S_0_-S_1_ electronic transitions. The absorption of blue and green light results in the yellow colour of the indenoquinoxaline solution, which takes on a different shade depending on the type of substituents present in its structure.

A characteristic feature of the absorption spectra of the synthesized indenoquinoxalines is the high intensity of the short-wavelength band, which is more than 10 times higher than the intensity of the long-wavelength band.

As can be seen from the data collected in Table 1 and the spectra shown in Figure 1 and Figure S1, the type of substituent in the indenoquinoxaline structure does not change the shape of the absorption bands but clearly affects their position. With respect to the parent dye (IND1), the presence of heavy atoms (–Cl, –Br, –I) in the indenoquinoxaline structure causes a slight shift (about 1–3 nm) of the absorption maximum toward longer wavelengths (IND1 vs. IND2, IND1 vs. IND3, IND1 vs. IND4; see Table 1). Only the introduction of a second chlorine atom to the indenoquinoxaline structure (IND8) causes a more significant shift of the long-wavelength band by 13 nm. Therefore, dyes containing more than one heavy atom will absorb the light emitted by the dental lamp more intensively.

Furthermore, the position of the absorption bands is clearly influenced by electron-donating (–CH_3_, –OCH_3_) and electron-withdrawing (–COOCH_3_) substituents in their structure. A strong bathochromic shift of the absorption band with a strong hyperchromic effect is observed after the introduction of the methoxy group containing lone electron pairs capable of donating a non-bonding electron to the conjugated double bond of the aromatic ring (IND1 vs. IND6). However, the presence of one alkyl substituent (-CH_3_) causes only a slight shift of the bands by about 3 nm towards longer wavelengths (IND1 vs. IND5) because its donor properties are weaker. The introduction of another methyl group increases the bathochromic shift to 8 nm (IND1 vs. IND9). IND6, IND5, and IND9 absorb visible light more efficiently than IND1 due to partial charge transfer from the oxygen (–OCH_3_) or carbon (–CH_3_) atom to the benzene ring. On the other hand, in the case of electron-withdrawing substituents, such as the ester group (-C(O)OCH_3_), which is able to accept an electron from the aromatic ring, a blue shift of the long-wavelength band (hypsochromic effect) is observed (IND1 vs. IND7, see Table 1).

Analysis of the obtained spectroscopic data showed that the modification of the indenoquinoxaline structure consisting of the introduction of electron-donating substituents and heavy atoms results in a shift of the absorption bands towards a longer wavelength and, consequently, their overlap with the visible region. This guarantees the absorption of radiation emitted by a dental lamp in the range of 390–500 nm used in the photopolymerization process.

### 2.2. Creating a Triplet State

**Figure 2:**
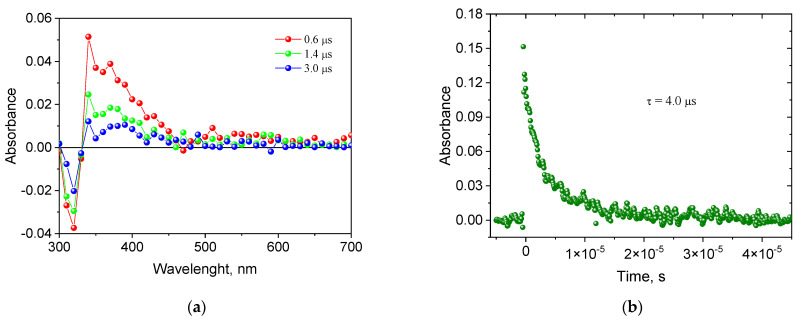
Transient absorption spectra (**a**) of IND2 in acetonitrile recorded at a different times after the pulse assigned to the triplet state and (**b**) transient absorption kinetic curve observed at 340 nm and 2 μs after the pulse for IND2.

The formation of the triplet state by the tested indenoquinoxalines was confirmed by recording the transient absorption spectra using the nanosecond laser flash photolysis technique. The spectral and kinetic characteristics of the triplet state of the exemplary indenoquinoxaline IND2 are shown in Figure 2. When excited with a 355 nm laser beam in a deoxygenated acetonitrile solution, the obtained indenoquinoxaline derivatives exhibit transient absorption that decays in the microsecond time scale. The absorption bands observed at 340 nm are attributed to the absorption of the triplet state. Analysis of the transient absorption kinetic curves (Figure 2b) allowed us to determine the lifetime of the triplet state. Its values range from 3.46 to 7.57 μs for IND1-IND10 (Table 2). It can be concluded that the lifetime of the triplet state of IND6 is approximately twice as long as that of IND1. This dye also shows the highest intersystem transition efficiency compared to the other synthesized derivatives, as evidenced by the triplet-state quantum yield value of 0.48.

The recorded transient absorption spectra confirm that these compounds generate a triplet state, which is especially important for the initiation of polymerization. The triplet state of the synthesized indenoquinoxalines was effectively quenched by the electron donor PhTAA used in the tested photoinitiating systems.

**Figure 3:**
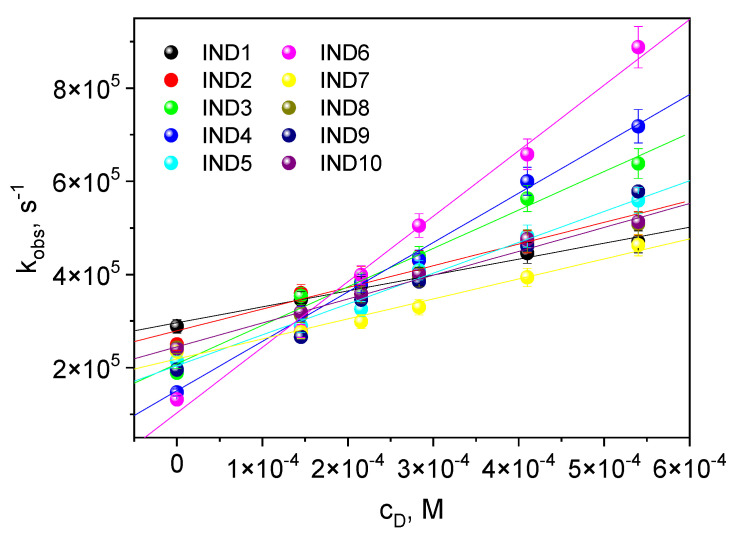
Stern–Volmer relationship of the triplet-state quenching process for the dyes studied ((phenylthio)acetic acid (PhTAA) was used as an electron donor (an excited state quencher)).

The quenching rate constant *k_q_* (Table 2) was determined from the triplet–triplet transient absorption spectra at a given wavelength using different quencher concentrations and the classical Stern–Volmer equation [30] (Figure 3):

(1) $$k_{obs}=\tau_{T}^{-1}+k_{q}[Q]$$

where *k_q_*—quenching rate constant of the excited state, *τ_T_*—lifetime of the excited state in the absence of an electron donor, and [*Q*]—molar concentration of the quencher.

The quenching rate constant is equal to the electron transfer rate constant when the only way to quench a triplet state is by an electron donor. It can then be assumed that,

(2) $$k_{q}=k_{el}$$

The conducted studies show that the rate of excited-state quenching is clearly influenced by the structure of the indenoquinoxaline derivatives. (Phenylthio)acetic acid effectively quenches IND4 and IND6 triple states. Therefore, they have a higher electron transfer rate constant than the other indenoquinoxalines tested and should better initiate the polymerization of triacrylates.

### 2.3. Photoinitiation of Free Radical Polymerization of Triacrylates

**Figure 4:**
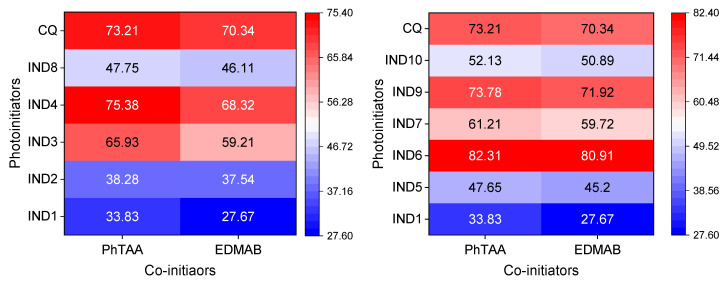
Heat maps of polymerization rate (μmol s^−1^) based on data obtained during photoinitiated polymerization using initiators: IND1-IND10 and CQ, and co-initiators: PhTAA and EDMAB (0.1 M). The light intensity of the dental lamp was 20 mW cm^−2^.

Favorable spectroscopic properties such as the intensity and position of absorption bands as well as the formation of a long-lived triplet excited state enabled the use of the indenoquinoxaline derivatives as photoinitiators in binary photoinitiating systems containing PhTAA or EDMAB as a co-initiator (electron donor). Figure 4 presents heat maps showing the dependence of the initial photopolymerization rate of TMPTA on the type of both the photoinitiator and co-initiator.

**Figure 5:**
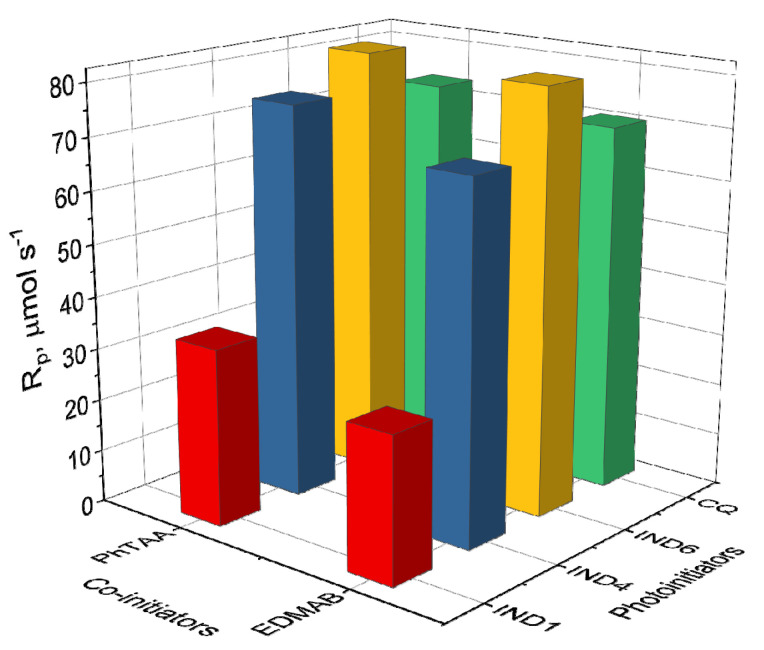
Comparison of the initial polymerization rates (R_p_, μmol s^−1^) of TMPTA determined for systems containing IND1, IND4, IND6, and CQ as photoinitiators and PhTAA and EDMAB at a concentration of 0.1 M as co-initiators. The light intensity of the dental lamp during photopolymerization was 20 mW cm^−2^.

In testing the possibility of using indenoquinoxalines in dentistry, the reference point for comparing the efficiency of photoinitiation of polymerization reactions was systems containing commercial compounds, e.g., camphorquinone (CQ), as a photoinitiator and ethyl *N*,*N*-dimethylaminobenzoate (EDMAB) as a co-initiator. As shown in Figure 5, the synthesized dyes (IND1-IND10) paired with the co-initiator PhTAA or EDMAB initiate the photopolymerization process at a rate similar to the commercial systems (e.g., CQ-PhTAA 73.21 μmol s^−1^ vs. IND4-PhTAA 75.38 μmol s^−1^ and IND6-PhTAA 82.31 μmol s^−1^). This indicates that the tested indenoquinoxalines are good photoinitiators of the free-radical polymerization of TMPTA in the range of light emitted by a dental lamp.

Moreover, it can be stated that (phenylthio)acetic acid (PhTAA) used in the tested photocurable compositions is an effective co-initiator. When comparing initial polymerization rates, it can be assumed that its electron-donating properties are comparable to those of ethyl-*N*,*N*-dimethylaminobenzoate (EDMAB) used in dentistry. In addition, the advantages of (phenylthio)acetic acid are its proven biological and antibacterial activity and wide medical application [31,32].

**Figure 6:**
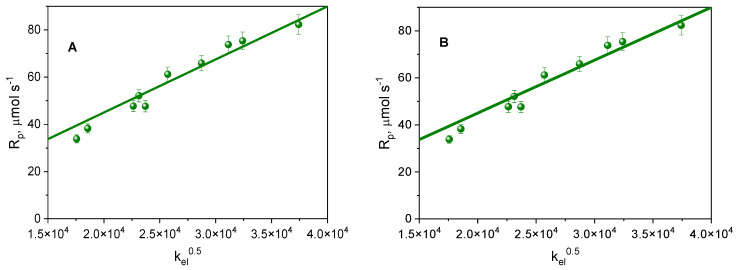
Dependence of the initial rate of photoinitiated polymerization of TMPTA on (**A**) the square root of the quantum yield of triplet-state formation, and (**B**) the square root of the electron transfer rate constant for the synthesized photoinitiators IND1-IND10. PhTAA (0.1 M) was used as a co-initiator. Light intensity emitted by a dental lamp was 20 mW cm^−2^.

As is known, free radicals that initiate polymerization in the case of binary systems can be formed, among others, as a result of the photoinduced electron transfer (PET) process. During the PET process, an electron is transferred from a co-initiator (electron donor) to an excited photoinitiator (electron acceptor) [33,34,35]. If radical formation occurs in the excited triplet state, then according to the kinetic equations, the initial rate of photoinitiated polymerization (*R_p_*) depends directly on the square root of the quantum yield of the triplet-state formation. As shown in Figure 6A, such a linear relationship was observed for the systems studied. It follows that the electron transfer process from the electron donor (PhTAA) to the photoinitiator (IND1–IND10) occurs in the excited triplet state.

For the photoinitiating systems studied, a rectilinear dependence of the initial rate of photoinitiated TMPTA polymerization on the square root of the electron transfer rate constant is also observed (Figure 6B). This confirms that the intermolecular electron transfer (PET) process limits the initial rate of free radical polymerization.

In summary, it can be stated that two-component photoinitiating systems based on indenoquinoxaline derivatives (IND1-IND10) and (phenylthio)acetic acid (PhTAA) may be of significant importance as innovative restorative materials for conservative dentistry. They can be deposited directly at the dentin–pulp interface, support regenerative functions, and cure quickly to form bulk polymers. In addition, the designed two-component photoinitiating systems effectively initiate the polymerization process, which results in a significant shortening of the polymerization time and a significant reduction in the photoinitiator concentration. This leads to savings due to lower financial outlays on materials and electrical energy.

### 2.4. Mass Stability

The factor that determines the durability of the filling is the mass stability of the dental composite. It is most often determined by sorption and solubility.

#### 2.4.1. Sorption

As a result of the interaction of dental fillings with the aqueous environment and fluids found in the oral cavity, unreacted monomers and small oligomers are eluted from them, and water is absorbed by the composite. The absorbed water occupies the space between the polymer chains or is bound to the polymer. This process is controlled by diffusion and lasts for several weeks [19,36]. Consequently, solvent sorption contributes to the weakening of the mechanical strength of the composite due to the reduced stability of the filler particle connection with the organic matrix [37].

Table S1 presents the average values of sorption changes obtained for the tested samples after 7, 14, 21, and 28 days of storage in solutions, i.e., distilled water, 3% acetic acid solution, artificial saliva, *n*-heptane, and coffee.

Comparison of sorption values for the tested samples allows us to state that under the influence of solutions such as distilled water, artificial saliva, or coffee, no statistically significant differences in sorption values are observed. However, differences occur in the case of hydrophobic solutions (*n*-heptane) and 3% acetic acid—the most aggressive of the used media.

**Figure 7:**
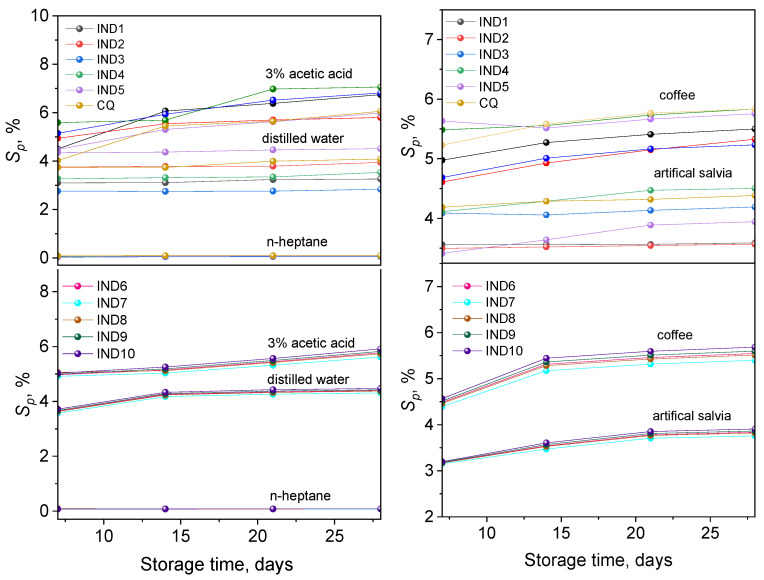
Dependence of the mean values of sorption changes in the tested IND1-IND10 and CQ materials on the conditioning time in 3% acetic acid solution, distilled water, artificial saliva, coffee, and *n*-heptane.

Figure 7 presents the dependencies of the average values of sorption changes on the conditioning time for the tested groups of solutions.

The data presented in Table S1 and Figure 7 show that the conditioning time of sample in solutions simulating the oral cavity environment affects the sorption of the tested materials. With the extension of the sample storage time in 3% acetic acid solutions simulating aqueous food with pH < 4.5, the average values of sorption changes (*S_p_*) increase. For samples stored in distilled water, artificial saliva, or coffee, sorption is set at a certain constant level after approx. 21 days of conditioning. After another 7 days of sample storage in these solutions, no changes in sorption are observed. On the other hand, in the case of samples conditioned in *n*-heptane simulating fatty food, the sorption values are the lowest and stable with the extension of storage time. Analyzing the experimental data presented in Figure 7 and Table S1, the sorption (*S_p_*) of the solution by the tested samples is the highest in the acidic environment, lower in coffee, distilled water, and artificial saliva, and the lowest in *n*-heptane. The values describing the sorption of the tested materials for all solutions are positive and range from 0.03 to 5.83%.

The most commonly used medium for determining the sorption capacity of commercial dental materials is distilled water and artificial saliva [17,19,37,38,39]. Research conducted by other authors for a wide range of composite materials confirms that this parameter can reach maximum values of 7% [19]. For our materials, the sorption values are lower than the limit value. This means that the tested material is comparable to commercial materials in terms of water and artificial saliva sorption.

In the systems characterized by the highest polymerization rate (e.g., containing photoinitiators IND6 and IND9), the lowest sorption values were observed in both aqueous and organic solution tests. A probable explanation for this phenomenon is a higher degree of monomer conversion achieved in a shorter time, resulting in a more densely crosslinked polymer structure. Reducing the amount of free space in the network makes it more difficult for liquid molecules to penetrate deep into the polymer matrix. Therefore, faster curing and, consequently, a higher network density significantly reduce sorption. A similar phenomenon was described by Tuna et al. [40]. The authors showed that an increased crosslinking rate in acrylic systems results in a reduction in the absorption of both water and organic solvents.

#### 2.4.2. Solubility

The solubility of the material is caused by the presence of solutions in the environment of the filled defect. This can lead to an unfavorable biological effect due to the washing out of unreacted substrates and polymer degradation products. In such cases, we are dealing with weakening of the connections between the filler and the organic matrix. The solubility phenomenon also leads to hydrolytic degradation of the composite material, which results in increased porosity and reduced strength parameters [4,16,19].

**Figure 8:**
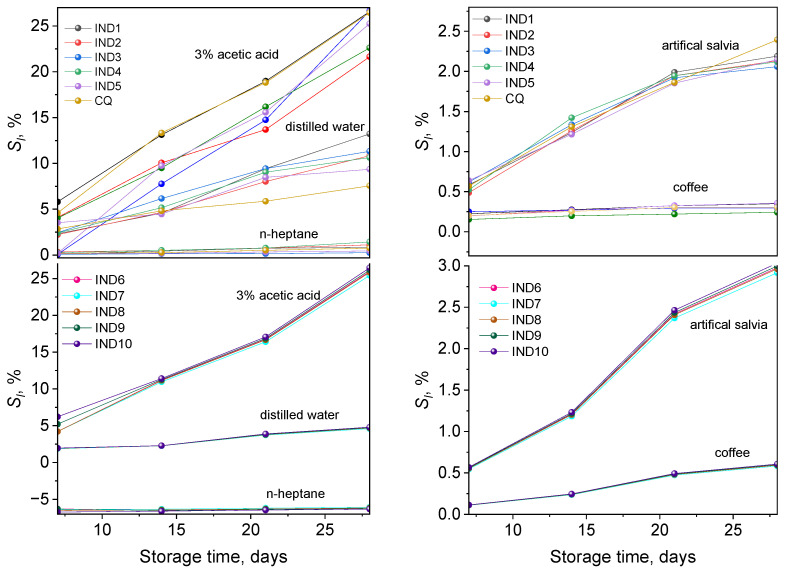
The dependence of the mean solubility values of the tested materials on the conditioning time in 3% acetic acid solution, artificial saliva, distilled water, *n*-heptane, and coffee.

The average solubility values obtained for the tested samples in distilled water, 3% acetic acid solution, artificial saliva, *n*-heptane, and coffee are summarized in Table S1, while Figure 8 illustrates the dependence of these values on the conditioning time.

When analyzing the data presented in Table S1 and Figure 8, the highest solubility (*S_l_*) of the samples occurs in the case of conditioning in a 3% acetic acid solution. Lower solubility values were obtained for samples tested in coffee, distilled water, and artificial saliva. On the other hand, the lowest solubility values are obtained for samples placed in *n*-heptane. As is known, the solubility of restorative materials depends on their structure. For example, the materials based on Bis-GMA are characterized by higher solubility in comparison with materials based on UDMA [41]. Experimental data confirm that after a properly conducted polymerization process, the solubility of composite materials should be low and within 2% [19,41,42,43]. Our research shows that the type of solution that is in contact with the filling material also affects the degree of solubility. Acidic solutions contribute to the increase in solubility more than water or artificial saliva [19,20]. They can cause erosion of the filler surface, which in turn leads to a reduction in the mass of the composite material and a weakening of its strength parameters [16].

#### 2.4.3. Change in Mass

The average values of the mass change in the tested samples in distilled water, 3% acetic acid solution, artificial saliva, *n*-heptane, and coffee are presented in Table S1, while Figure S2 shows the course of the dependence of changes in this parameter in selected solutions simulating the oral cavity environment on the conditioning time.

The largest mass changes occur in samples stored in coffee, distilled water, and artificial saliva, while the smallest changes occur in 3% acetic acid solution and slight changes occur in *n*-heptane. The mass changes for samples stored in 3% acetic acid solution and *n*-heptane are negative. The obtained results for the tested composites are comparable with the literature values [44].

### 2.5. Colour Stability

Colour stability is of the greatest importance from the point of view of aesthetics of dental fillings. Colour changes depend primarily on the material’s ability to support the sorption of pigments and dyes from solutions, the properties of the matrix, especially its hydrophilicity, filler content, and particle size, as well as surface roughness.

**Figure 9:**
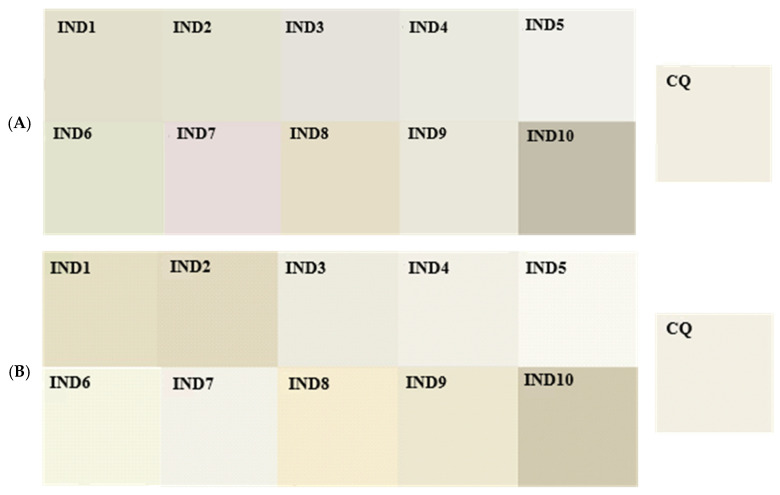
Colour of the tested materials IND1-IND10 and CQ (**A**) before and (**B**) after 28 days of conditioning in artificial saliva.

Figure 9 shows the colours of the tested materials, obtained based on the average values of L, a, and b coordinates in the Lab colour space, marked on the surface of the tested material samples, respectively, before and after 28 days of conditioning in artificial saliva.

Based on the average values obtained of the L, a, and b parameters, the values of the colour change (Δ*E*) of the tested materials were calculated, depending on the type of solution used and the conditioning time (Table 3). The colour measurements were compared to the ideal whiteness according to the standard tooth whiteness chart (*L* = 98.93, *a* = −0.28, *b* = 0.54).

Table 3 presents the values of the colour change (Δ*E*) of the tested materials, calculated based on the values of the *L*, *a*, and *b* parameters, depending on the type of solution used and the conditioning time.

The colour stability results (Table 3) of the tested materials in the applied solutions simulating the oral cavity environment at specific conditioning times show that statistically significant differences in colour in relation to ideal white coincide with the point of exceeding the clinically acceptable Δ*E* limit. Colour changes for materials for which Δ*E* takes values below 1 are imperceptible to the human eye. Slightly higher Δ*E* values in the range of 1–3.3 are noticeable to the human eye but clinically acceptable. This means that it is possible to place pairs of materials for which Δ*E* < 1 is in the immediate vicinity, because the difference in their colour is impossible to observe. As the colour difference in the range of 1–3.3 is noticeable only to a qualified observer, this value is considered clinically acceptable; therefore, materials showing a Δ*E* value in this range can also remain in close proximity [45]. It should be noted, however, that the colour of the enamel is an individual feature of each person and is rarely perfectly white. The shade of teeth can range from white to slightly yellowish or grayish. The enamel itself is semi-transparent, and its final colour results from the colour of the dentin underneath. The following factors influence the colour of enamel: genetics, enamel thickness, diet, oral hygiene, age, fluorosis, medications, and diseases. Thinner enamel makes the colour of the dentin (usually yellowish) more visible, which can cause a darker shade of teeth [46].

**Figure 10:**
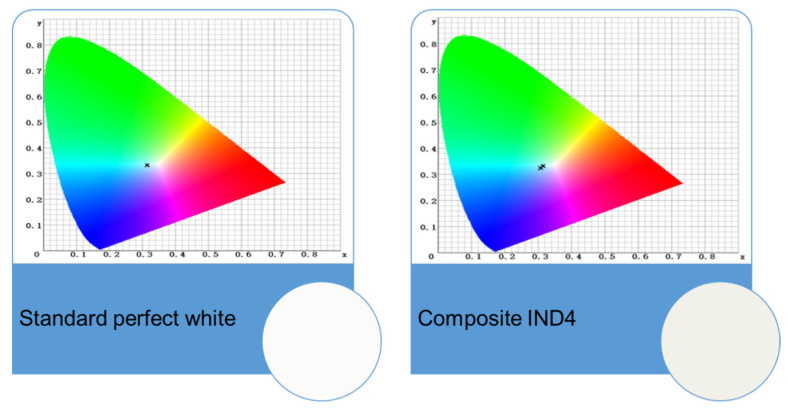
Colour xy coordinates for the perfect white standard and the composite containing IND4. The colour of the composite is shown in the circle inset.

An analysis of the data presented in Table 3 showed that in the solution simulating a greasy, hydrophobic environment (*n*-heptane), the colour changes are the smallest. This is probably due to the significant hydrophilicity of the tested materials and is consistent with the lowest *n*-heptane sorption. Increasing the conditioning time results in a decrease in colour stability in most cases. Significant colour changes occur in a 3% acetic acid solution. Acetic acid, as the most aggressive, causes the dissolution of materials and significant loss of mass, thus affecting the initial proportion of components and changing the original colour. In this case, a significant decrease in Δ*E* is observed, which for the composite containing IND4 takes the value of 3.07. Therefore, if this composite is placed in a tooth cavity with ideal whiteness, only a qualified observer will notice the difference (Figure 10). The greatest colour changes are observed in the solution containing pigments, i.e., coffee. The explanation for this phenomenon is the significant hydrophilicity of the tested materials, resulting in the sorption of solutions containing pigments, which as a consequence leads to discoloration. The Δ*E* values after 28 days of conditioning of the composites in coffee reach the highest values. On the other hand, the commercial reference material CQ is characterized by colour stability similar to composites containing IND3, IND4, and IND8.

The tested experimental materials, apart from monomers, initiators, and fillers, did not contain any additives, such as colour stabilizers. Even though the clinically acceptable parameters are exceeded already at the initial stage of the conditioning process, it should also be remembered that the conditions used are extreme. The exception is the use of distilled water and, above all, artificial saliva, the presence of which in the oral cavity is constant. A step towards achieving increased colour stability of the experimental materials is the addition of colour stabilizers.

## 3. Materials and Methods

### 3.1. Reagents

Reagents for the synthesis of photoinitiators: ninhydrin, o-phenylenediamine, 4-chloro-o-phenylenediamine, 4-bromo-1,2-diaminobenzene, 4-methyl-o-phenylenediamine, 4-methoxy-o-phenylenediamine dihydrochloride, methyl 3,4-diamonbenzoate, 4,5-dichloro-o-phenylenediamine, 4,5-dimethyl-1,2-phenylenediamine, 4-iodo-1,2-diaminobenzene, 2,3-diaminopyridine; and solvents for synthesis, photopolymerization, spectroscopic and chromatographic measurements, as well as mass and colour stability, i.e., acetonitrile, acetic acid, ethyl acetate, 1-methyl-2-pyrrolidinone, *N*,*N*-dimethylformamide, *n*-heptane, and deuterated dimethyl sulfoxide (DMSO-*d*_6_), were purchased from Merck Chemical Co. (Darmstadt, Germany).

A monomer, trimethylolpropane triacrylate (TMPTA); a commercial photoinitiator, camphorquinone (CQ); and electron donors, (phenylthio)acetic acid (PhTAA) and ethyl 4-dimethylaminobenzoate (EDMAB) were purchased from Sigma-Aldrich. An inorganic filler, neutral dental glass IDG (Inter Dental Glass) GM 35429 (with the composition in wt.%: SiO_2_–30, CaO–10, Al_2_O_3_–30, F–15, P_2_O_5_ < 10, Na_2_O < 10), was purchased from Schott Dental Glass Co., Wolverhampton, UK.

Salivia sintetica CTS (SSC) used in the mass and colour stability study was purchased from C.T.S. s.r.l. Italy. The coffee solution was prepared by placing 2.5 g of instant coffee (Jacobs Kronung) in 250 mL of boiling water (according to the manufacturer’s instructions).

TLC plates, of the thin layer (DC-Plastikfolien Silica gel 60 F_254_, 0.2 mm) and preparative (Silica gel 60 F_254+366_, 2 mm) chromatography types, were sourced from Merck.

### 3.2. Methods

#### 3.2.1. Nuclear Magnetic Resonance

Nuclear magnetic resonance spectra were recorded in deuterated dimethyl sulfoxide (DMSO-d_6_) on a Bruker AscendTM 400 NMR spectrophotometer.

#### 3.2.2. HPLC Analyses

HPLC analyses were performed by HPLC systems equipped with a UV-Vis detector (detection wavelength was 400 nm), Binary HPLC pump, and Symmetry C18 column (3.5 μm, 4.6 × 75 mm). Separation was conducted under isocratic conditions with a 1.0 mL/min flow rate at r.t., 10 μL injection volume, and HPLC-grade MeOH as a mobile phase.

#### 3.2.3. Electronic Absorption Spectra

Electronic absorption spectra in an ethyl acetate solution were recorded using a Shimadzu UV-vis Multispec-1501 spectrophotometer.

#### 3.2.4. Photopolymerization

The classical microcalorimetric method was used to study the kinetics of photoinitiated polymerization [47,48,49]. Detailed information about the setup used is given in our previous article [50]. The photocurable mixture contained 0.1 mL of 1-methyl-2-pyrrolidone (MP) acting as a solvent and 0.9 g of 2-ethyl-2-hydroxymethyl-1,3-propanediol triacrylate (TMPTA) monomer. The synthesized indenoquinoxaline derivatives IND1-IND10 were used as photoinitiators at a concentration of 1.12 × 10^−3^–1.80 × 10^−3^ M, depending on the molar absorption coefficient. The co-initiator was a (phenylthio)acetic acid (PhTAA) at a concentration of 0.1 M. For each sample, the measurements were repeated three times. The photoinitiation efficiency of the polymerization reaction was compared to a system containing a commercial photoinitiator camphorquinone (CQ) and a co-initiator commonly used in dentistry, ethyl 4-dimethylaminobenzoate (EDMAB). The concentration of the camphorquinone was 0.675 M, while the concentration of the co-initiator was 0.1 M.

#### 3.2.5. Lifetime of an Excited Triplet State

The lifetime of the excited triplet state of the synthesized photoinitiators was determined in a deoxygenated acetonitrile solution based on the transient absorption spectra recorded on nanosecond LSK 60 Laser Flash Photolysis (Applied Photophysics, Leatherhead, UK). A pulsed laser model LPY 150 from Lambda Physik emitting radiation at 355 nm was used to excite the sample. The energy of the laser pulses was 65 mJ.

#### 3.2.6. Quantum Yield of Triplet-State Formation

The quantum yield of triplet-state formation was determined using the method described by Lament et al. [51] and by us [48] based on the analysis of transition absorption spectra recorded on a nanosecond flash photolysis apparatus.

#### 3.2.7. The Rate Constant of Quenching of the Excited Triplet State

The rate constant of quenching of the excited triplet state was determined based on the transient absorption spectra recorded during nanosecond flash photolysis measurements. (Phenylthio)acetic acid (PhTAA) was used as the quencher at a concentration of 1.50 × 10^−3^–5.50 × 10^−3^ M.

#### 3.2.8. Mass Stability Test

To study mass stability, five basic solutions simulating the natural environment of the oral cavity during consumption of various types of meals were prepared [52,53,54,55,56]. The following were used: distilled water, which corresponded to the environment created by hydrated food with pH > 4.5, artificial saliva simulating that naturally present in the oral cavity, *n*-heptane creating the environment of fatty food, a 3% acetic acid solution corresponding to hydrated food with pH < 4.5, and a solution containing pigments—coffee.

The method of marking and the sorption and solubility limits of polymeric restorative materials are regulated by the PN-EN ISO 4049 standard [57]. The prepared samples, which are equivalent to dental fillings, are weighed (m_1_), placed in 10 mL of the above-mentioned solutions, and conditioned at 37 °C for 7, 14, 21, and 28 days [16,17,18,37,38,42,58,59]. After a specified storage time, the samples of the tested materials were dried with a paper towel to remove the solution absorbed on the surface, weighed (m_2_), then dried to a constant weight (± 0.1 mg) in a desiccator at 37 °C in the presence of silica gel as a drying agent and weighed again (m_3_). After each completed cycle, the samples are placed back in freshly prepared solutions. The time after which the subsequent values of sorption, solubility, and mass changes are determined is summed up. Mass stability tests were performed on five samples of each tested composite (n = 5) for four ageing times: 7, 14, 21, and 28 days.

Sorption (S_p_) of each of the tested solutions, solubility (S_l_), and mass change (D_m_) are determined based on Formulas (3)–(5), respectively:

(3) $$S_{p}=\left(\frac{m_{2}-m_{3}}{m_{2}}\right)\cdot 100\%$$

(4) $$S_{l}=\left(\frac{m_{1}-m_{3}}{m_{1}}\right)\cdot 100\%$$

(5) $$D_{m}=\frac{m_{2}-m_{1}}{m_{1}}\cdot 100\%$$

where

*S_p_*—the mass that is reversibly absorbed during storage in relation to the mass of the swollen sample.

*S_l_*—irreversible mass change, unchanged after drying in a desiccator in relation to the initial mass of the sample.

*D_m_*—change in mass during storage before drying in relation to the initial mass of the sample.

#### 3.2.9. Colour Stability Test

The colour of the tested dental filling materials was determined based on colorimetric measurements in the CIE Lab colour space, performed on an SV-100/300 spectrophotometer. The CIE Lab scale allows for the numerical value of colour to be presented using three coordinates: L, a, and b. Changes along the axes of the individual parameters determine changes in lightness (L: 0 for black materials and 100 for white materials), colour changes from green to red (a: −120 to +120, respectively), and colour changes from blue to yellow (b: −120 to +120, respectively). In the CIE Lab colour space, the colour change is determined based on Equation (6):

(6) $$∆E=\sqrt{∆L^{2}+∆a^{2}+∆b^{2}}$$

where ΔL, Δa, and Δb denote changes in the values of individual coordinates between the tested sample and the reference sample [60].

In order to determine statistically significant differences in the initial colour of the tested materials and the values of L, a, and b coordinates in the Lab colour space, the colour was marked on the surface of samples corresponding to dental fillings before the conditioning process and 28 days after placing the samples in distilled water, artificial saliva, *n*-heptane, 3% acetic acid solution, and a solution containing pigments—coffee. For each sample (n = 3), three measurements of changes in the L*, a*, and b* coefficients were performed, and the reported ΔE values are the arithmetic means of these three measurements ± standard deviation.

### 3.3. Design of the Photoinitiator Structure

Ten organic compounds based on the 11H-indeno[1,2-b]quinoxaline-11-one (IND1-IND10) skeleton were designed for this study. This skeleton has a specific structure, as it consists of four fused rings. Three of them are six-membered aromatic rings, one of which has two nitrogen heteroatoms. The fourth one forms a cyclopentanone system. These structural conditions provide the molecule with a flat and highly rigid structure that prevents rotation of the benzene rings as well as isomerization. As a result, channels for deactivation of excited states are eliminated. This aspect is extremely important when designing compounds for use in photochemical processes, including photopolymerization [35,61,62].

Furthermore, quinoxalines are common structural skeletons found in many drugs and bioactive compounds [63,64]. It has been proven that heterocyclic systems containing oxygen, nitrogen, sulfur, and other heteroatoms in five- and six-membered rings often exhibit pharmaceutical and biological activity, including anti-inflammatory, cardiotonic, antimicrobial, and antibacterial effects [65,66,67,68,69]. The biological activity of 11H-indeno[1,2-b]quinoxaline-11-one has also been documented as an excellent inhibitor of platelet-derived growth factor receptor tyrosine kinase [63].

When designing compounds containing the indenoquinoxaline system as photoinitiators for applications in dentistry, we considered the influence of individual structural fragments on their spectroscopic properties. Of particular importance was the introduction of substituents or groups inducing the bathochromic effect to shift the absorption band into the visible region, corresponding to the emission range of dental lamps. This was aimed at increasing the efficiency of free radical polymerization initiation by the designed dental compositions. Therefore, electron-donating substituents (IND5, IND6), an electron-accepting substituent (IND7), and heavy atoms (IND2-IND4) were introduced into the basic structure of 11H-indeno[1,2-b]quinoxaline-11-one (IND1) in position 7 or 8. Disubstituted derivatives in positions 7 and 8 (IND8 and IND9) as well as a compound containing a pyridinium group (IND10) were also obtained.

#### Synthesis—General Procedure for Obtaining Indenoquinoxalines

**Figure 11:**
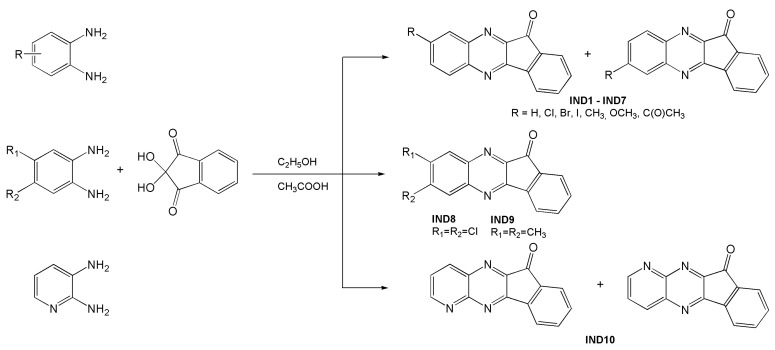
Synthesis of 11H-indeno[1,2-b]quinoxaline-11-one derivatives (IND1-IND10).

The first and fundamental task was to develop a universal method for the synthesis of indenoquinolines, which would be simple, easy, and would give high yields of the final product. The applied synthesis is based on the condensation of 1,2-diaminobenzene derivatives with ninhydrin in ethanol in the presence of a catalytic amount of glacial acetic acid. The schematic route for the synthesis of indenoquinoxalines (IND1-IND10) is shown in Figure 11.

Ninhydrin (1.78 g, 0.01 mol) and an equimolar amount of the appropriate derivative of 1,2-diaminobenzene (4-chloro-o-phenylenediamine, 4-bromo-1,2-diaminobenzene, 4-methyl-o-phenylenediamine, 4-methoxy-o-phenylenediamine dihydrochloride, methyl 3,4-diamonbenzoate, 4,5-dichloro-o-phenylenediamine, 4,5-dimethyl-1,2-phenylenediamine) or 2,3-diaminopyridine were placed in a round-bottomed flask. Ethanol (50 mL) and three drops of glacial acetic acid were added. The reaction mixture was stirred at room temperature until the raw materials were completely dissolved and then heated to reflux. Product formation was already observed after a few minutes of heating. The progress of the condensation was monitored by thin-layer chromatography (TLC) on silica gel 60F 254 with chloroform as the eluent. The reaction time was approximately 1 h. The isolated product was crystallized from *N*,*N*-dimethylformamide to obtain crystals in a 88–96% yield. The products were identified spectroscopically and by HPLC analysis. The NMR spectra and chromatograms are included in the Supplementary Materials. TLC chromatography showed that seven of the obtained indenoquinoxalines containing a substituent in position 7 or 8 (IND2-IND7 and IND10) are a mixture of isomers. Two compounds IND8 and IND9 are disubstituted derivatives of indenoquinoxaline with substituents in positions 7 and 8.

IND1; 11H-indeno[1,2-b]quinoxaline-11-one

Reagents: ninhydrin—1.78 g (0.01 mol); o-phenylenediamine—1.08 g (0.01 mol).

Product: C_15_H_8_N_2_O; light yellow crystals; 232.24 g/mol; yield 2.10 g (91%); m.p. 224–225 °C (lit. 225–226 °C [70,71,72]); R_f_ = 0.79, R_T_ = 1.136 min.

^1^H NMR (400 MHz, DMSO-d_6_) δ (ppm): 8.20–8.14 (t, 2H), 8.11–8.09 (d, ^3^J_H,H_ = 8 Hz, 1H), 7.95–7.84 (m, 4H), 7.74–7.70 (t, 1H)

^13^C{^1^H} NMR (100 MHz, DMSO-d_6_) δ (ppm): 189.8, 156.9, 150.3, 142.6, 142.3, 141.5, 137.4, 137.1, 133.2, 132.9, 131.4, 130.8, 129.8, 124.7, 122.7

IND2; mixture of isomers: 7-chloro-11H-indeno[1,2-b]quinoxaline-11-one and 8-chloro-11H-indeno[1,2-b]quinoxaline-11-one

Reagents: ninhydrin—1.78 g (0.01 mol); 4-chloro-o-phenylenediamine—1.42 g (0.01 mol).

Product: C_15_H_7_ClN_2_O; yellow crystals; 266.68 g/mol; yield 2.39 g (90%); m.p. 233–234 °C, R_f_ = 0.65, R_T_ = 1.321 min.

^1^H NMR (400 MHz, DMSO-d_6_) δ (ppm): 8.14–8.08 (m, 2H), 8.04–8.03 (m, 2H), 8.02–8.01 (m, 2H), 7.87–7.85 (m, 2H), 7.74–7.69 (m, 2H), 7.63–7.61 (d, ^3^J_H,H_ = 8 Hz, 2H), 7.58–7.53 (m, 2H).

^13^C{^1^H} NMR (100 MHz, DMSO-d_6_) δ (ppm): 189.4, 157.4, 156.6, 150.0, 149.3, 143.5, 142.9, 141.6, 141.3, 141.2 141.1, 138.5, 136.9, 136.9, 136.8, 136.6, 136.0, 133.1, 132.8, 132.7, 132.5, 131.1, 130.7, 130.3, 128.8, 124.9, 124.8, 122.7, 122.6.

IND3; mixture of isomers: 7-bromo-11H-indeno[1,2-b]quinoxaline-11-one and 8-bromo-11H-indeno[1,2-b]quinoxaline-11-one.

Reagents: ninhydrin—1.78 g (0.01 mol) and 4-bromo-1,2-diaminobenzene—1.87 g (0.01 mol).

Product: C_15_H_7_BrN_2_O; yellow crystals; yield—2.79 g (90%); 311.13 g/mol; m.p. 238–240 °C, R_f_ = 0.63, R_T_ = 1.359 min.

^1^H NMR (400 MHz, DMSO-d_6_) δ (ppm): 8.24–8.18 (m, 2H), 8.15–8.08 (m, 4H), 7.98–7.96 (d, ^3^J_H,H_ = 8 Hz, 2H), 7.84–7.78 (m, 2H), 7.74–7.71 (dd, 2H), 7.68–7.64 (m, 2H).

^13^C{^1^H} NMR (100 MHz, DMSO-d_6_) δ (ppm): 189.44, 157.45, 149.38, 143.55, 141.20, 141.17, 138.56, 136.99, 136.92, 136.88, 133.19, 132.90, 132.76, 132.55, 131.16, 130.71, 130.35, 128.82, 124.93, 124.86, 122.78, 122.63.

IND4; mixture of isomers: 7-iodo-11H-indeno[1,2-b]quinoxaline-11-one and 8-iodo-11H-indeno[1,2-b]quinoxaline-11-one.

Reagents: ninhydrin—1.78 g (0.01 mol) and 4-iodo-1,2-diaminobenzene—2.34 g (0.01 mol).

Product: C_15_H_7_IN_2_O; yellow crystals; yield 3.18 g (89%); 358.13 g/mol; m.p. 242–243 °C, R_f_ = 0.60, R_T_ = 1.340 min.

^1^H NMR (400 MHz, DMSO-d_6_) δ (ppm): 8.56–8.49 (m, 2H), 8.05–7.93 (m, 4H), 7.88–7.85 (m, 3H), 7.77–7.70 (m, 2H), 7.58–7.55 (t, 2H).

^13^C{^1^H} NMR (100 MHz, DMSO-d_6_) δ (ppm): 189.39, 143.66, 141.91, 141.15, 139.20, 138.77, 136.93, 132.87, 132.78, 132.47, 130.76, 124.90, 124.84, 122.75, 122.69.

IND5; mixture of isomers: 7-methyl-11H-indeno[1,2-b]quinoxaline-11-one and 8-methyl-11H-indeno[1,2-b]quinoxaline-11-one.

Reagents: ninhydrin—1.78 g (0.01 mol) and 4-methyl-o-phenylenediamine—1.22 g (0.01 mol).

Product: C_16_H_10_N_2_O, yellow crystals; yield 2.21 g (90%); 246.26 g/mol, m.p. 175–177 °C (lit. 176 °C [73,74]), R_f_ = 0.74, R_T_ = 1.244 min.

^1^H NMR (400 MHz, DMSO-d_6_) δ (ppm): 8.04 (s, 2H), 8.02–7.98 (m, 2H), 7.93–7.91 (m, 1H), 7.84 (s, 2H), 7.82–7.81 (m, 2H), 7.70–7.65 9 (m, 2H), 7.53–7.48 (m, 3H), 2.54–2.52 (s, 6H).

^13^C{^1^H} NMR (100 MHz, DMSO-d_6_) δ (ppm): 190.18, 190.10, 156.81, 155.98, 149.18, 148.39, 143.70, 143.22, 142.72, 141.73, 141.59, 141.55, 141.11, 141.05, 136.73, 136.65, 136.63, 136.47, 134.65, 132.46, 132.35, 132.20, 131.12, 130.61, 129.16, 128.88, 124.71, 124.67, 122.38, 122.27, 22.06, 21.74.

IND6; mixture of isomers: 7-methoxy-11H-indeno[1,2-b]quinoxaline-11-one and 8-methoxy-11H-indeno[1,2-b]quinoxaline-11-one.

Reagents: ninhydrin—1.78 g (0.01 mol) and 4-methoxy-o-phenylenediamine dihydrochloride—2.11 g (0.01 mol).

Product: C_16_H_10_N_2_O_2_; yellow crystals; yield 2.35 g (90%); 262.26 g/mol; m.p. 240–242 °C [75], R_f_ = 0.71, R_T_ = 1.189 min.

^1^H NMR (400 MHz, DMSO-d_6_) δ (ppm): 8.26–7.41 (14H), 4.058 (s, 3H), 4.00 (s, 3H)

^13^C{^1^H} NMR (100 MHz, DMSO-d_6_) δ (ppm): 161.08, 136.72, 136.44, 136.01, 132.62, 131.88, 130.37, 125.15, 124.71, 124.65, 123.07, 122.01, 109.38, 107.85, 55.92.

IND7; mixture of isomers: methyl 11-oxo-11H-indeno[1,2-b]quinoxaline-8-carboxylate and methyl 11-oxo-11H-indeno[1,2-b]quinoxaline-7-carboxylate.

Reagents: ninhydrin—1.78 g (0.01 mol) and methyl 3,4-diamonbenzoate—1.66 g (0.01 mol).

Product: C_17_H_10_N_2_O_3_; yellow crystals; yield 2.55 g (88%); 290.27 g/mol; m.p. 268–270 °C, R_f_ = 0.79, R_T_ = 1.177min.

^1^H NMR (400 MHz, DMSO-d_6_) δ (ppm): 8.67–8.63 (m, 2H), 8.37–8.34 (dd, 2H), 8.29–8.24 (m, 2H), 8.16–8.14 (d, ^3^J_H,H_ = 8 Hz, 2H), 7.95–7.90 (m, 4H), 7.79–7.76 (m, 2H), 3.97 (s, 6H).

^13^C{^1^H} NMR (100 MHz, DMSO-d_6_) δ (ppm): 189.23, 165.74, 158.59, 151.73, 144.92, 141.67, 141.09, 137.64, 137.57, 133.91, 132.79, 131.70, 131.18, 130.47, 124.84, 132.23, 53.25.

IND8; 7,8-dichloro-11H-indeno[1,2-b]quinoxaline-11-one.

Reagents: ninhydrin—1.78 g (0.01 mol) and 4,5-dichloro-o-phenylenediamine—1.77 g (0.01 mol).

Product: C_15_H_6_ClN_2_O; yellow crystals; yield 2.79 g (93%); 301.13 g/mol; m.p. 208–210 °C, R_f_ = 0.59, R_T_ = 1.593 min.

^1^H NMR (400 MHz, DMSO-d_6_) δ (ppm): 8.35 (s, 1H), 8.26(s, 1H), 8.14–8.12 (d, ^3^J_H,H_ = 8 Hz, 1H), 7.98–7.96 (d, ^3^J_H,H_ = 8 Hz, 1H), 7.85–7.81 (t, 1H), 7.69–7.66 (t, 1H).

^13^C{^1^H} NMR (100 MHz, DMSO-d_6_) δ (ppm): 188.99, 157.34, 150.16, 141.84, 141.34, 140.99, 137.20, 137.08, 136.90, 134.87, 133.10, 131.82, 130.29, 124.97, 122.87.

IND9; 7,8-dimethyl-11H-indeno[1,2-b]quinoxaline-11-one.

Reagents: ninhydrin—1.78 g (0.01 mol) and 4,5-dimethyl-1,2-phenylenediamine—1.36 g (0.01 mol).

Product: C_17_H_12_N_2_O; yellow crystals; yield 2.39 g (92%); 260.29 g/mol; m.p. 255–257 °C, R_f_ = 0.61, R_T_ = 1.343 min.

^1^H NMR (400 MHz, DMSO-d_6_) δ (ppm): 8.21–8.23 (d, ^3^J_H,H_ = 8 Hz, 1H), 8.01–7.92 (m, 3H), 7.80–7.76 (t, 1H), 7.64–7.60 (t, 1H), 2.55 (s, 3H), 2.53 (s, 3H).

^13^C{^1^H} NMR (100 MHz, DMSO-d_6_) δ (ppm): 148.45, 144.11, 144.00, 141.70, 141.27, 141.20, 136.68, 136.57, 132.41, 130.83, 128.58, 128.53, 124.69, 122.79, 122.68, 20.62, 20.29.

IND10; mixture of isomers: 6H-indeno[1,2-b]pyrido[3,2-e]pyrazin-6-one and 10H-indeno[1,2-b]pyrido[2,3-e]pyrazin-10-one.

Reagents: ninhydrin—1.78 g (0.01 mol) and 2,3-diaminopyridine—1.78 g (0.01 mol).

Product: C_14_H_7_N_3_O; yellow crystals; yield 2.23 g (96%); 233.22 g/mol; m.p. 300–302 °C, R_f_ = 0.69, R_T_ = 0.978 min.

^1^H NMR (400 MHz, DMSO-d_6_) δ (ppm): 9.17–9.12 (m, 2H), 8.66–8.63 (m, 1H), 8.62–8.59 (m, 1H), 8.19–8.17(m, 1H), 8.13–8.11 (m, 1H), 7.96–7.92 (m, 4H), 7.90–7.87 (m, 2H), 7.80–7.7.74 (m, 2H).

^13^C{^1^H} NMR (100 MHz, DMSO-d_6_) δ (ppm): 189.44, 188.93, 159.99, 157.60, 155.72, 154.02, 153.17, 151.53, 151.33, 141.14, 141.00, 140.26, 138.73, 138.63, 137.86, 137.72, 137.61, 137.51, 133.88, 133.63, 127.68, 236.14, 124.86, 124.81, 123.38, 122.97.

Analysis of the ^1^H NMR spectra showed no signal from the amino group at 3.0–5.0 ppm and the appearance of a new signal in the ^13^C NMR spectrum at 150.0–160.0 ppm from the carbon atoms of the –C=N– bond, indicating the formation of a condensation product. Furthermore, analysis of NMR spectra confirms that some of the compounds exist in isomeric form. However, from our previous work on isomers of indoloquinoxaline, pyrazolo[3,4-b]quinoxaline, pentaazacyclopenta[b]naphthalene, and pyridopyrazinoindole [76,77], it follows that the isomeric structures show a very similar photoinitiation efficiency of TMPTA polymerization. Therefore, in the presented work, the isomeric mixtures were not separated.

### 3.4. Preparation of Dental Composites

The obtained indenoquinoxaline derivatives were used to develop new, alternative dental composites. They consisted of two main components, i.e., a liquid organic matrix, undergoing cross-linking polymerization, and an inorganic filler, influencing the final properties of the composite.

The liquid organic matrix of the tested photocurable composites contained a photoinitiator, a co-initiator, a solvent, and a monomer. The exact composition of the organic matrix is given in Section 3.2.4. Filler (neutral dental glass—IDG, 1.5 g) was added to the prepared liquid organic matrix. The ratio of organic matrix to filler was 40:60 (% wt.). The whole was thoroughly mixed, and the prepared composite dental fillings were subjected to photocuring. The obtained cylindrical samples simulating dental fillings were used for mass and colour stability tests.

In the case of commercial composites, other auxiliary substances are also added, e.g., light stabilizers, which are responsible for preventing changes in the filling colour, and compounds that allow for matching the shade of the filling to the natural colour of the patient’s teeth, as well as inhibitors preventing premature polymerization during storage [23,25,78,79,80]. The experimental materials prepared in this work, which are equivalent to dental fillings, did not contain auxiliary substances allowing us to obtain real measurement results for the pure composite.

To verify the properties of experimentally obtained dental composites, the tested systems were compared with samples containing commercial compounds commonly used in dentistry: photoinitiator—camphorquinone (CQ), and co-initiator—ethyl 4-dimethylaminobenzoate (EDMAB) at concentrations of 0.675 M and 0.1 M, respectively.

## 4. Conclusions

In recent years, there has been a significant increase in the use of composite materials in dentistry. Currently, there are almost 200 diverse types of composite materials available on the market, which indicates a high demand and dynamic development of this segment. In response to market needs, new two-component photoinitiating systems based on indenoquinoxaline derivatives (IND1-IND10) and (phenylthio)acetic acid (PhTAA) have been developed. The designed systems are the main components of the liquid organic matrix constituting the basis of dental composites. Innovative composites based on indenoquinoxaline derivatives photopolymerize at a high speed (25–30 s), comparable to or higher in comparison with literature values for other experimental and commercial dental fillings. Assessment of the durability of the designed composites based on ageing tests allowed for the assessment of the behavior of these materials under the long-term impact of the oral cavity environment. It was found that the type of solution simulating the natural environment of the oral cavity has the greatest impact on the hydrolytic stability and colour stability of the tested material. The newly designed composites allow for precise matching of the colour and structure of the filling to the natural tooth, which is important for patients who care about the aesthetics of their smile.

In connection with this, the shortening of the photopolymerization time and the high attractiveness of the newly obtained composites based on indenoquinoxalines allow us to state that they can be of immense importance as innovative, restorative materials for regenerative dentistry.

## Figures and Tables

**Table 1:** Spectroscopic properties of the tested dyes in ethyl acetate.

Dye	$\lambda_{\max}^{\mathrm{Abs}.}$, nm	ε_max_, M^−1^·cm^−1^	Dye	$\lambda_{\max}^{\mathrm{Abs}.}$, nm	ε_max_, M^−1^·cm^−1^
IND1	287 381	34,600 3500	IND6	299 398	37,400 4560
IND2	275 382	33,100 3200	IND7	277 374	32,500 4400
IND3	279 383	34,200 3350	IND8	294 403	34,200 3660
IND4	293 384	35,100 3700	IND9	305 398	36,200 3880
IND5	294 384	35,500 3820	IND10	301 395	35,900 3650

**Table 2:** The initial rate of photoinitiated polymerization (*R_p_*), quantum yield of the triplet state (*Φ_T_*), lifetime of triplet-state formation (*τ_T_*), and quenching rate constant of the triplet state (*k_q_*) for the compounds studied.

Dye	*R_p_*, μmol s^−1^		*Φ_T_*	*τ_T_*, μs	*k_q_* *, M^−1^s^−1^
	PhTAA	EDMAB			
IND1	33.83	27.67	0.08	3.46	3.09 × 10^8^
IND2	38.28	37.54	0.1	4.00	3.45 × 10^8^
IND3	65.93	59.21	0.24	5.29	8.25 × 10^8^
IND4	75.38	68.32	0.31	6.68	1.05 × 10^9^
IND5	47.65	45.20	0.16	4.65	5.62 × 10^8^
IND6	82.31	80.91	0.48	7.57	1.40 × 10^9^
IND7	61.21	59.72	0.18	4.25	6.61 × 10^8^
IND8	47.75	46.11	0.11	4.10	5.12 × 10^8^
IND9	73.78	71.92	0.28	5.10	9.69 × 10^8^
IND10	52.13	50.89	0.13	4.15	5.35 × 10^8^

* Excited state quencher: (phenylthio)acetic acid (PhTAA).

**Table 3:** Colour change values (Δ*E*) of the tested materials calculated based on the values of parameters L, a, and b depending on the type of solution used and the conditioning time; Δ*E* > 3.3 denotes a clinically unacceptable colour change.

Dye	Storage Time, Days					
	7	28	7	28	7	28
	Distilled Water		Artificial Saliva		3% Acetic Acid	
IND1	13.42 ± 0.44	13.02 ± 0.21	13.65 ± 0.62	13.36 ± 0.56	13.35 ± 0.26	11.10 ± 0.96
IND2	13.02 ± 0.021	11.42 ± 0.27	12.73 ± 0.26	12.72 ± 0.48	12.54 ± 0.31	9.83 ± 0.11
IND3	10.20 ± 0.25	9.99 ± 0.31	10.28 ± 0.31	10.17 ± 0.73	10.33 ± 0.45	7.72 ± 0.49
IND4	7.82 ± 0.32	6.09 ± 0.42	7.91 ± 0.98	7.85 ± 0.51	7.45 ± 0.24	3.07 ± 0.24
IND5	14.87 ± 0.45	14.63 ± 0.27	14.99 ± 0.24	14.27 ± 0.32	14.79 ± 0.38	11.03 ± 0.91
IND6	15.83 ± 0.39	15.00 ± 0.24	15.22 ± 0.19	15.05 ± 0.68	15.44 ± 0.25	11.25 ± 0.74
IND7	11.33 ± 0.25	10.58 ± 0.38	11.25 ± 0.52	11.01 ± 0.46	11.13 ± 0.82	5.18 ± 0.31
IND8	10.59 ± 0.49	9.95 ± 0.52	10.99 ± 0.57	10.41 ± 0.31	10.13 ± 0.27	7.17 ± 0.87
IND9	12.88 ± 0.35	11.71 ± 0.17	12.89 ± 0.49	12.48 ± 0.49	12.75 ± 0.41	13.82 ± 0.75
IND10	16.97 ± 0.28	16.05 ± 0.42	16.63 ± 0.56	15.99 ± 0.56	16.63 ± 0.38	16.48 ± 0.58
CQ	9.90 ± 0.45	8.48 ± 0.27	9.43 ± 0.78	8.82 ± 0.32	9.43 ± 0.28	6.72 ± 0.14
	*n*-heptane		coffee			
IND1	13.22 ± 0.40	13.20 ± 0.21	13.22 ± 0.32	5.11 ± 0.48		
IND2	12.53 ± 0.72	12.50 ± 0.32	12.53 ± 0.24	13.59 ± 0.21		
IND3	10.63 ± 0.57	10.58 ± 0.58	10.63 ± 0.58	11.26 ± 0.57		
IND4	7.83 ± 0.63	7.63 ± 0.31	7.83 ± 0.62	8.91 ± 0.77		
IND5	14.52 ± 0.28	14.48 ± 0.74	14.52 ± 0.34	15.92 ± 0.21		
IND6	15.02 ± 0.55	14.96 ± 0.82	15.02 ± 0.27	17.28 ± 0.66		
IND7	11.31 ± 0.79	11.02 ± 0.62	11.31 ± 0.44	12.59 ± 0.49		
IND8	10.59 ± 0.82	10.29 ± 0.31	10.59 ± 0.51	12.63 ± 0.45		
IND9	12.62 ± 0.91	12.55 ± 0.29	12.62 ± 0.72	14.98 ± 0.69		
IND10	16.03 ± 0.32	15.98 ± 0.11	16.03 ± 0.23	18.23 ± 0.45		
CQ	9.37 ± 0.27	9.07 ± 0.25	9.37 ± 0.11	11.54 ± 0.82		

## Data Availability

All data generated or analyzed during this study are included in this published article and its Supplementary Materials**.**

## Supplementary Materials

The following supporting information can be downloaded at: https://www.mdpi.com/article/10.3390/ijms26115424/s1.
