# Supplementary material for: The Latest Achievements in the Design of Permanent Fillings for Conservative Dentistry Based on Indenoquinoxaline Derivatives as Photoinitiators of Visible-Light Polymerization: Mass and Colour Stability
Source: Int J Mol Sci. 2025 Jun 5;26(11):5424. doi: 10.3390/ijms26115424 (PMC12155062; doi:10.3390/ijms26115424)
Supplement: Supplementary file 1 [file ijms-26-05424-s001.zip › ijms-3657620-supplementary.pdf]

Supporting information

The latest achievements in the design of permanent fillings for conservative dentistry based on indenoquinoxaline derivatives as photoinitiators of visible light polymerization. Mass and color stability

Ilona Pyszka*, Oliwia Szczepańska, Beata Jędrzejewska

Faculty of Chemical Technology and Engineering, Bydgoszcz University of Science and Technology, 85-326 Bydgoszcz, Poland

* Correspondence: Ilona.Pyszka@pbs.edu.pl (I.P.); Tel.: +48-52-374-9039 (I.P.)

Table of contents **Page**

^{1}H and ^{13}C NMR spectra …………………………………………………………………………….. S2

HPLC chromatograms …………………………………………………………………..……... S22

Normalized electronic absorption spectra in ethyl acetate – Fig. S1 …………………………….. S27

Average values of solubility, sorption and mass change for the samples tested in distilled water, 3% acetic acid solution, artificial saliva, *n*-heptane and coffee – Table S1 ……………… S28

Dependence of the mean values of mass change of the tested materials on the conditioning time in selected solutions simulating the oral cavity environment – Fig. S2 ………………….. S32

^{1}H spectrum of **IN1**

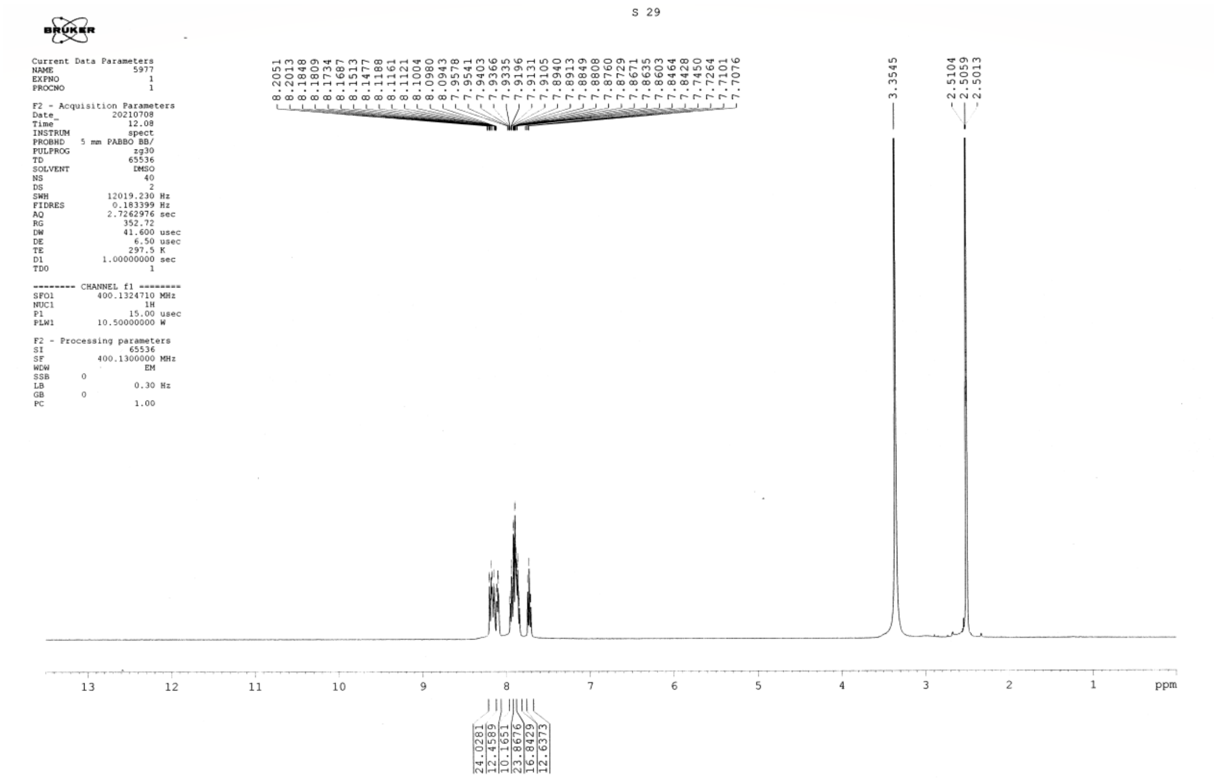

Enlarged spectrum in the range of 7-9.

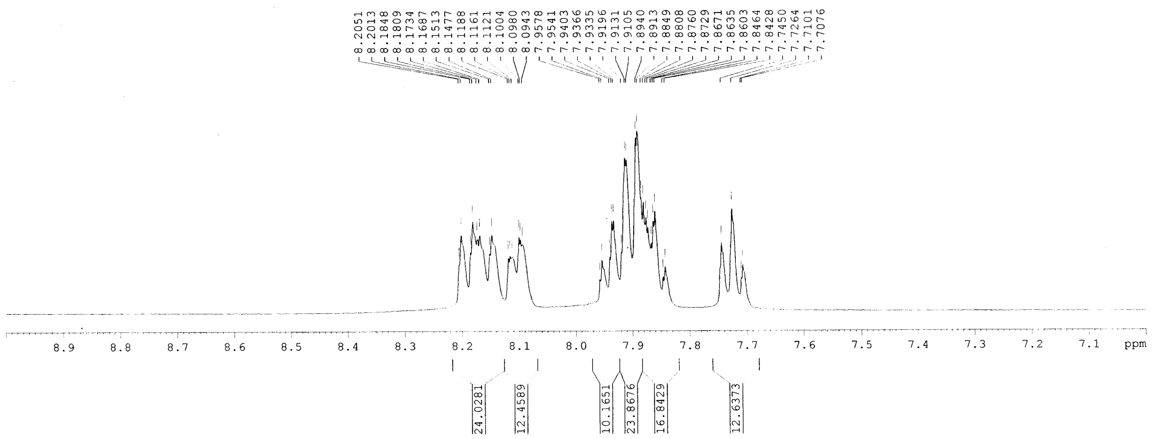

^{13}C spectrum of **IN1**

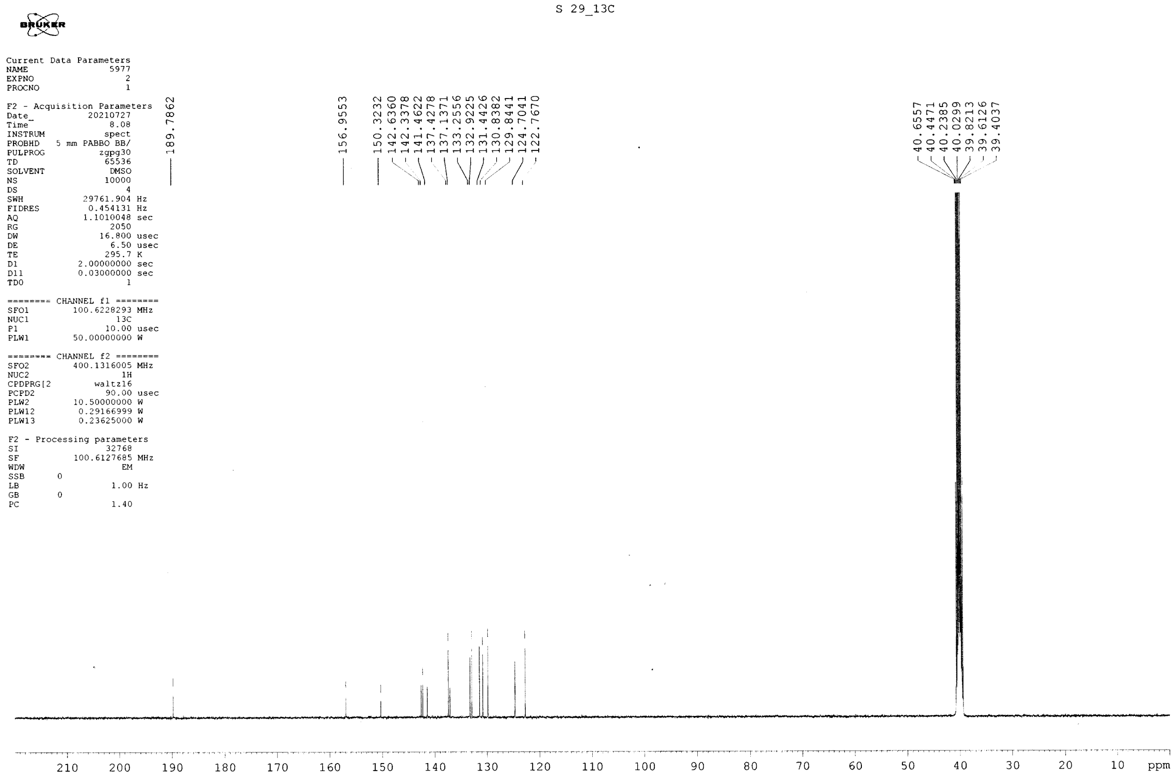

Enlarged spectrum in the range of 110-180 ppm.

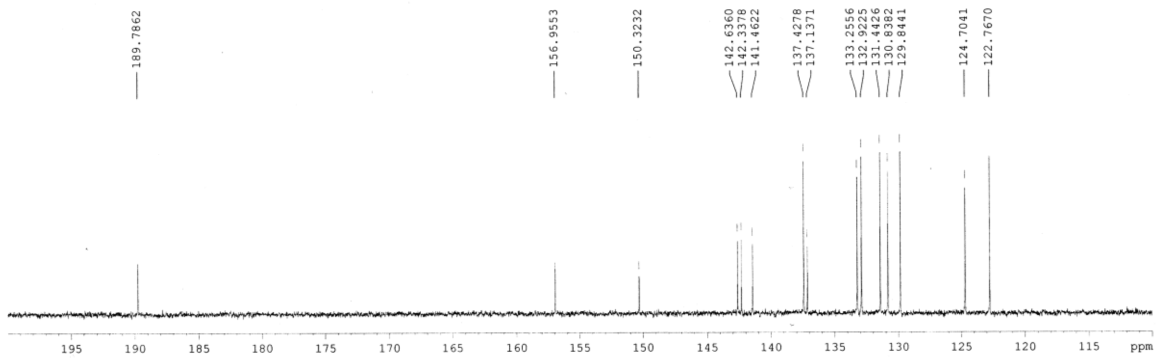

^{1}H spectrum of **IN2**

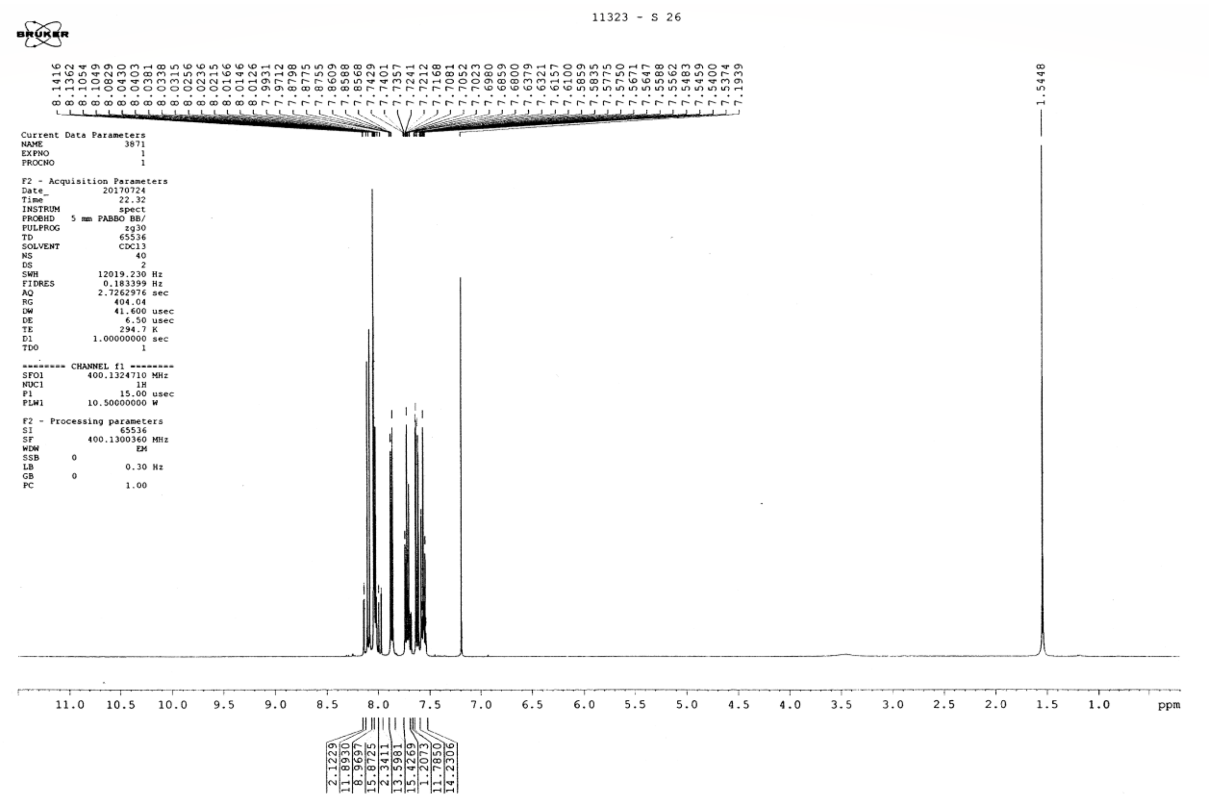

Enlarged spectrum in the range of 7-9.

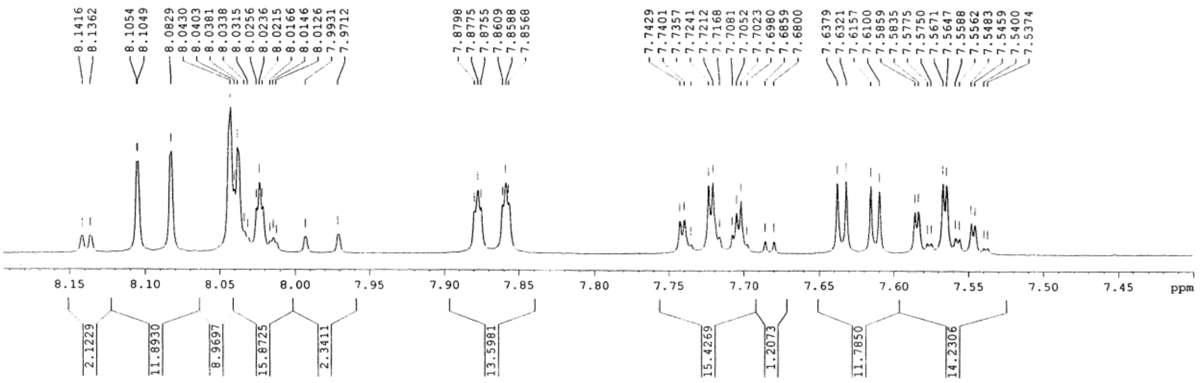

^{13}C spectrum of **IN2**

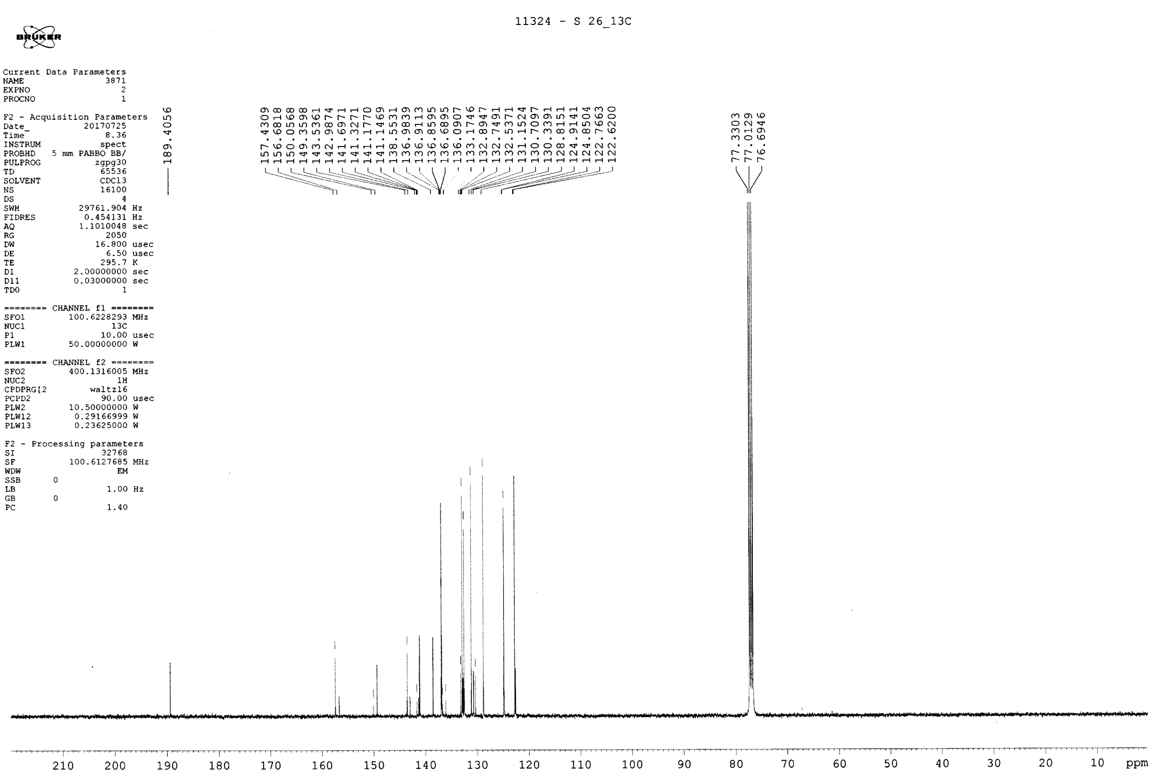

Enlarged spectrum in the range of 120-200 ppm.

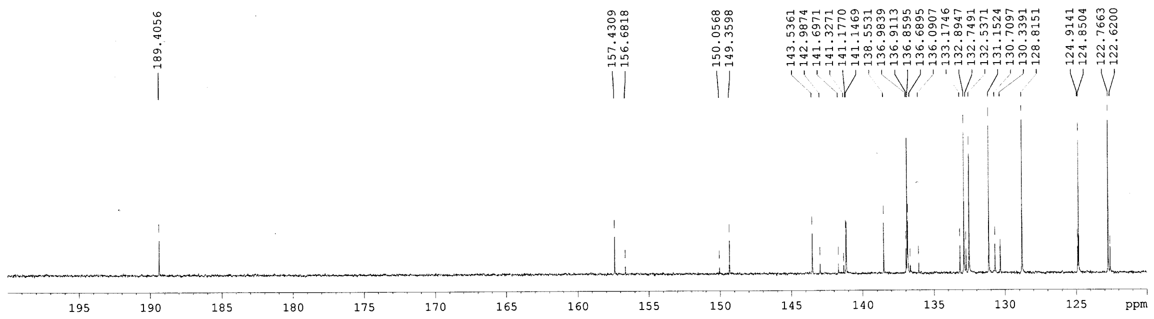

^{1}H spectrum of **IN3**

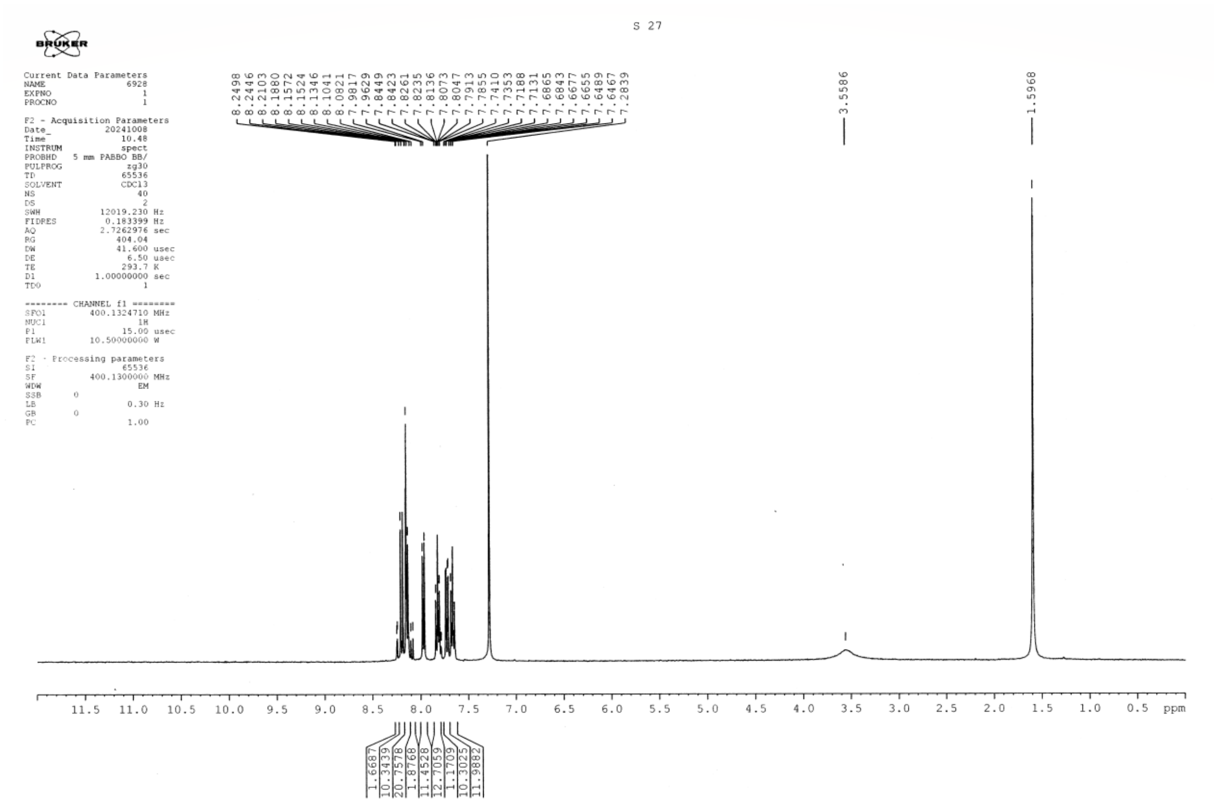

Enlarged spectrum in the range of 7-9 ppm.

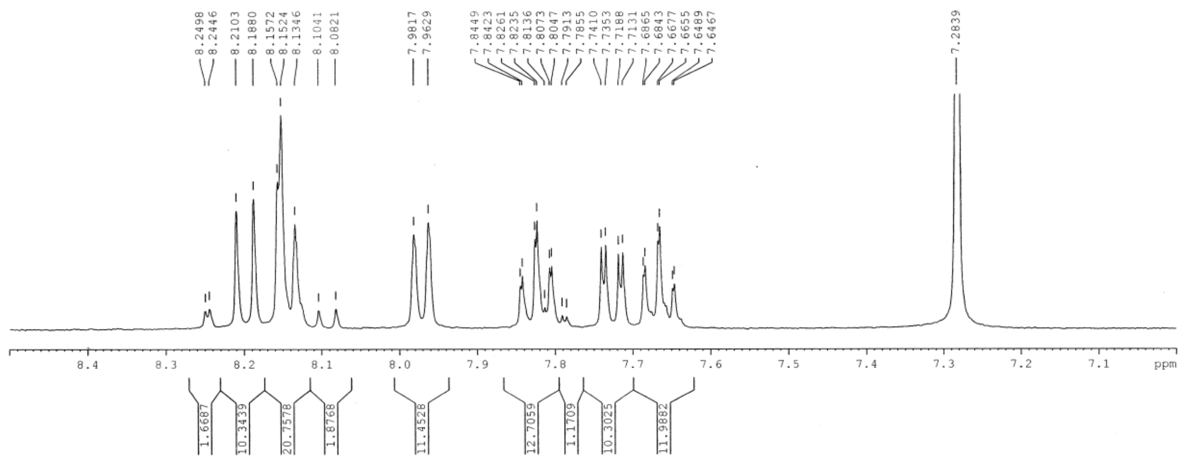

^{13}C spectrum of **IN3**

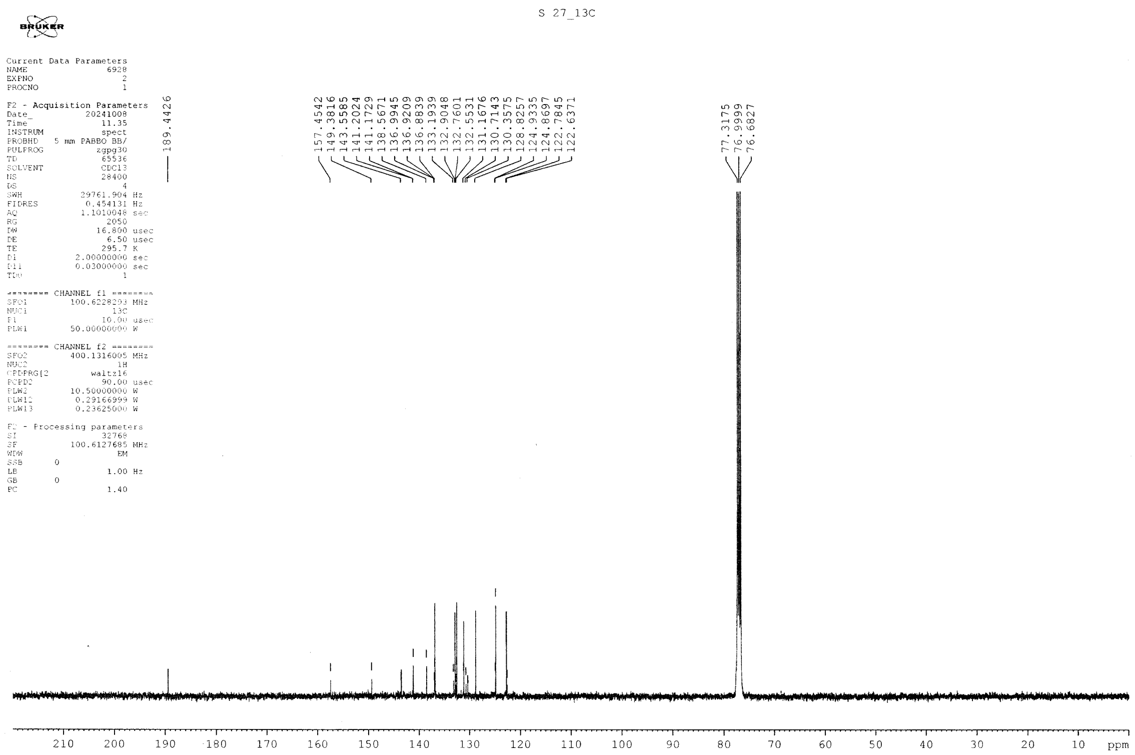

Enlarged spectrum in the range of 110-150 ppm.

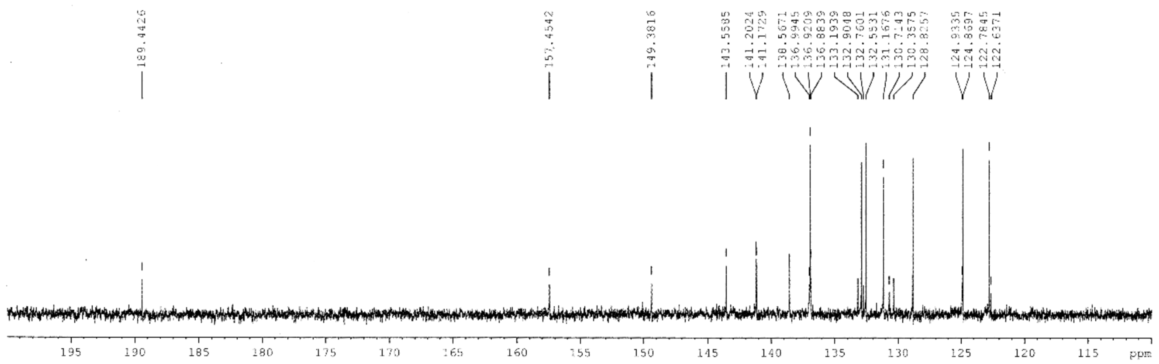

^{1}H spectrum of **IN4**

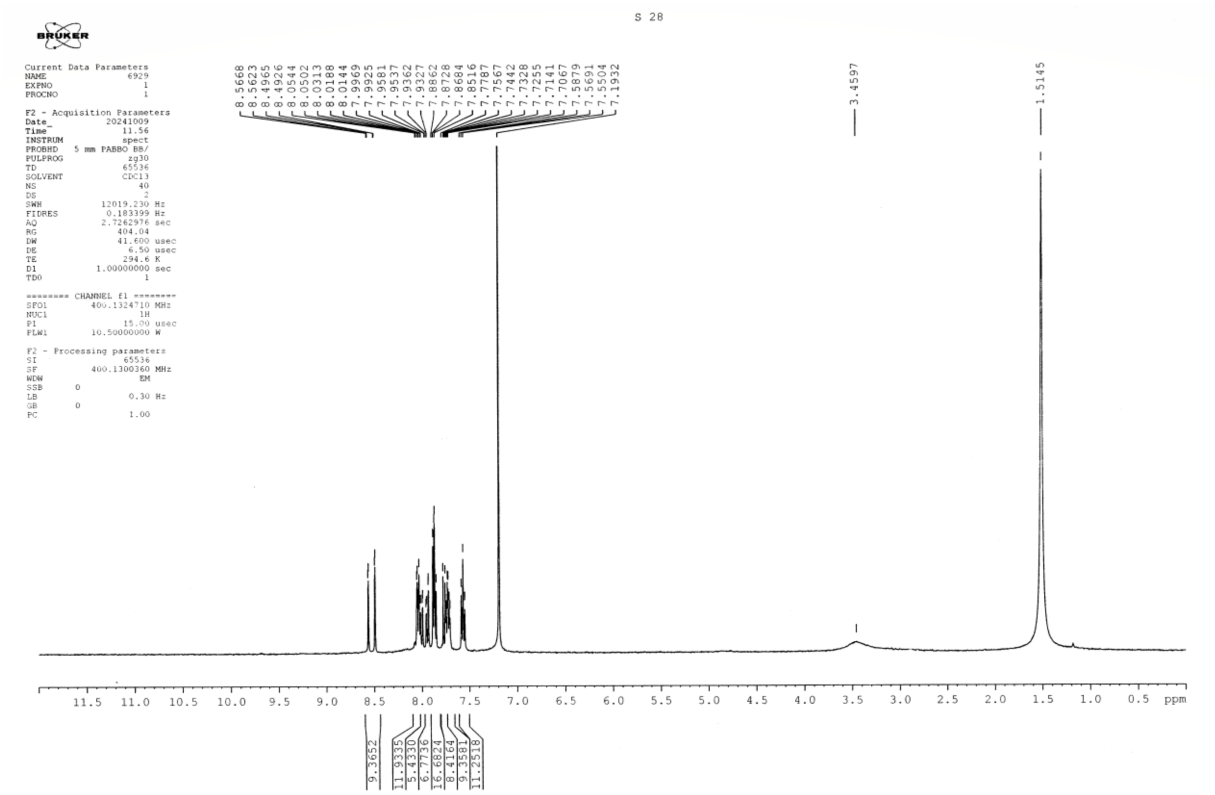

Enlarged spectrum in the range of 7-9 ppm.

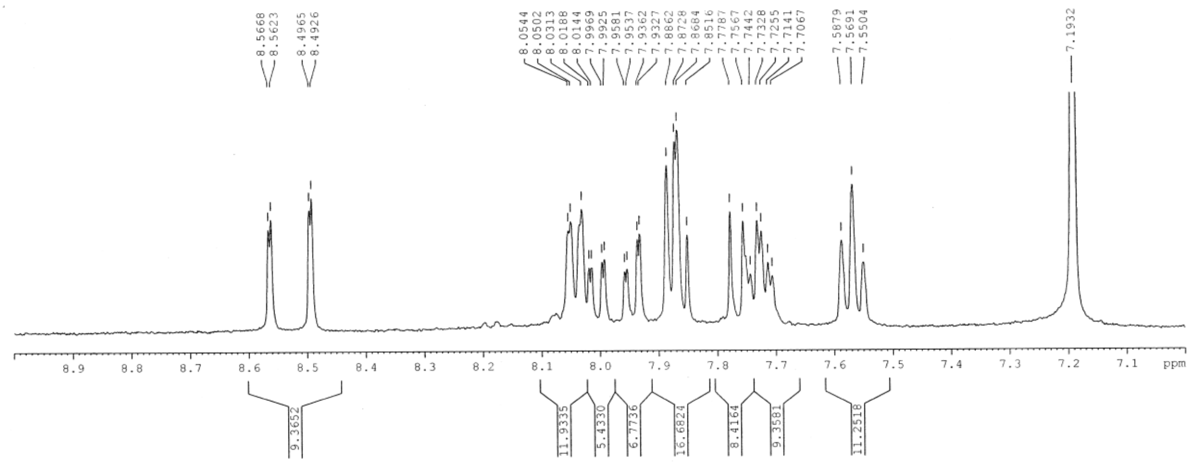

^{13}C spectrum of **IN4**

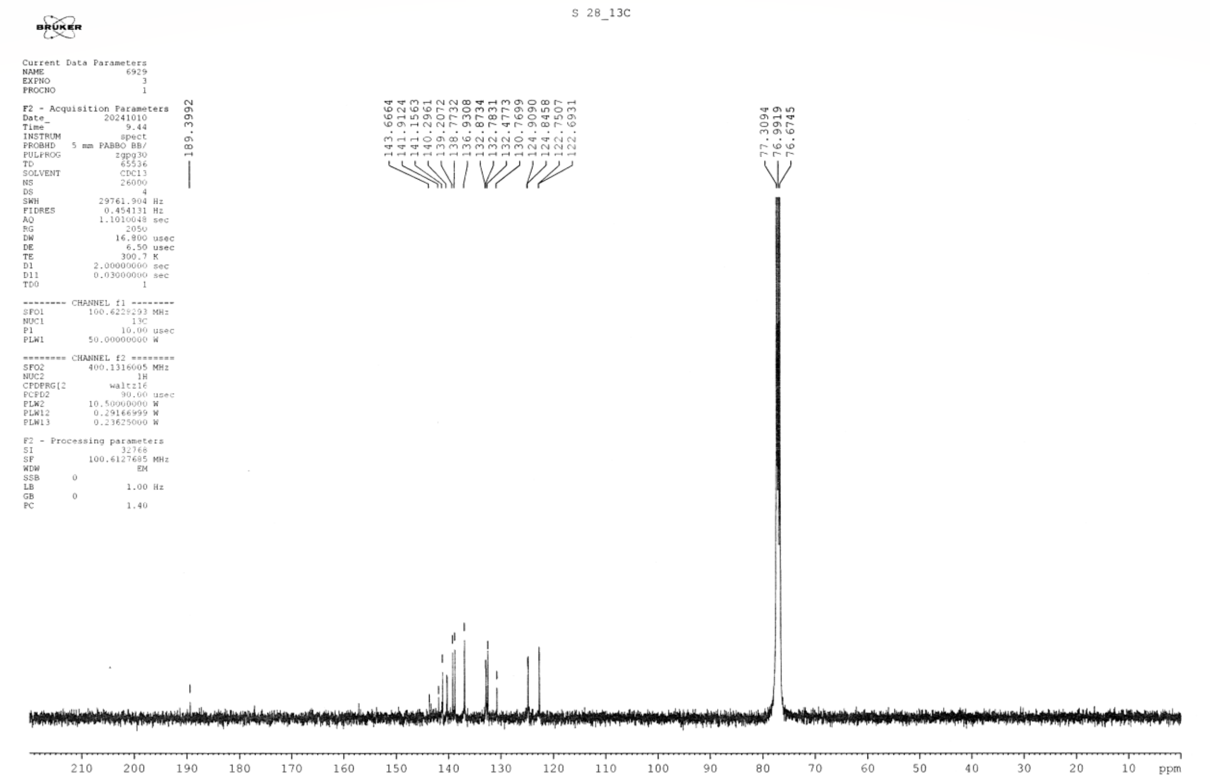

Enlarged spectrum in the range of 110-150 ppm.

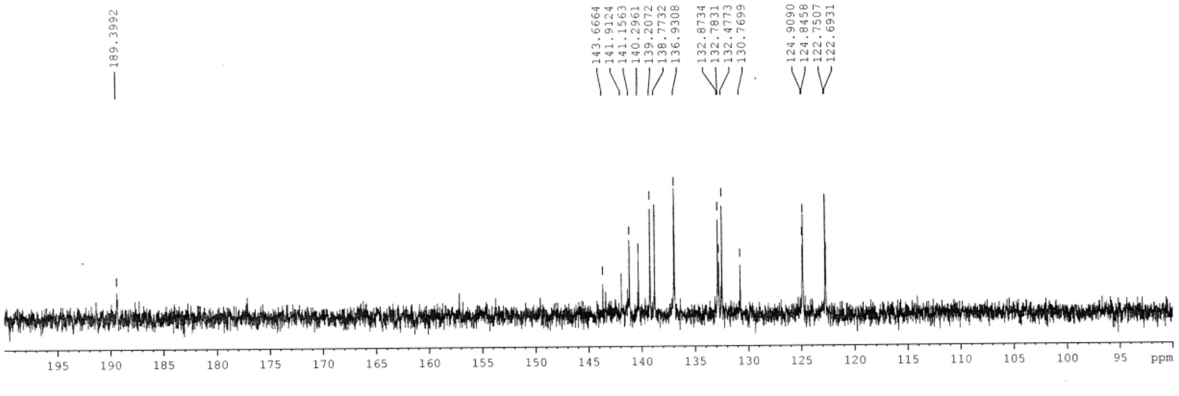

^{1}H spectrum of **IN5**

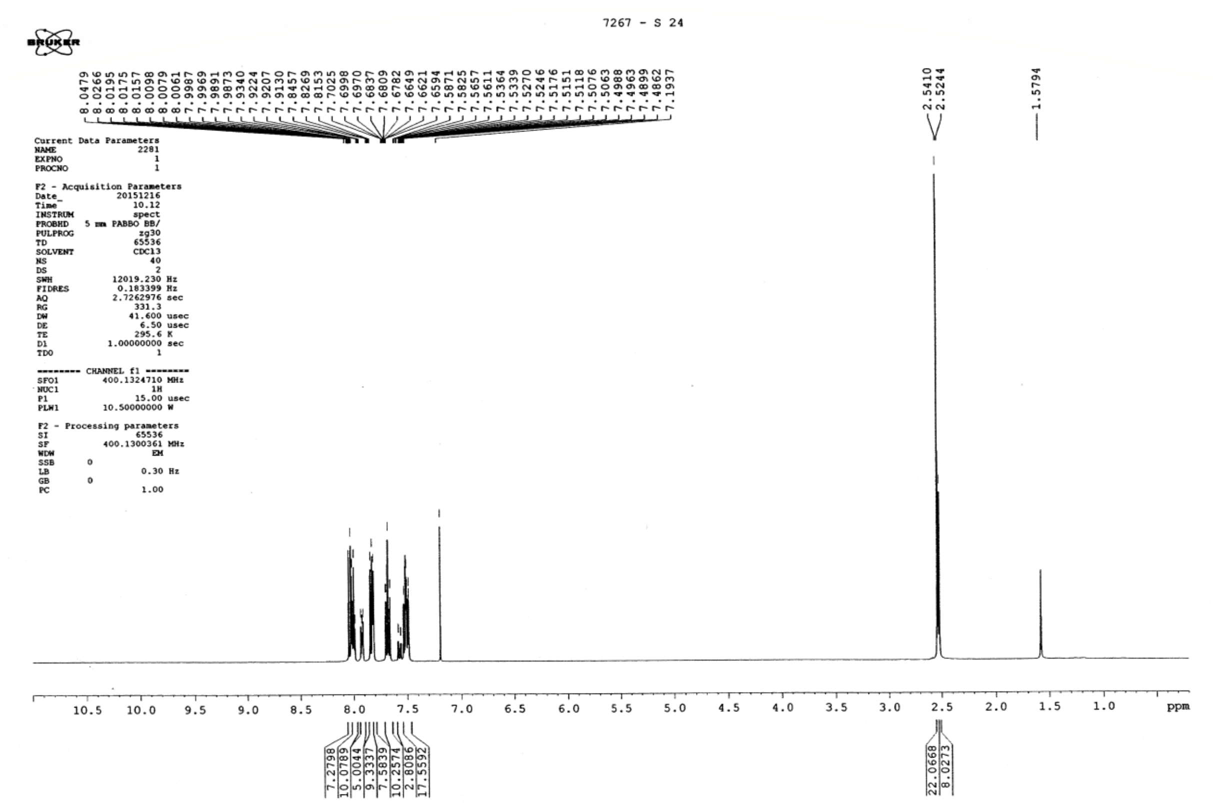

Enlarged spectrum in the range of 7-9 ppm.

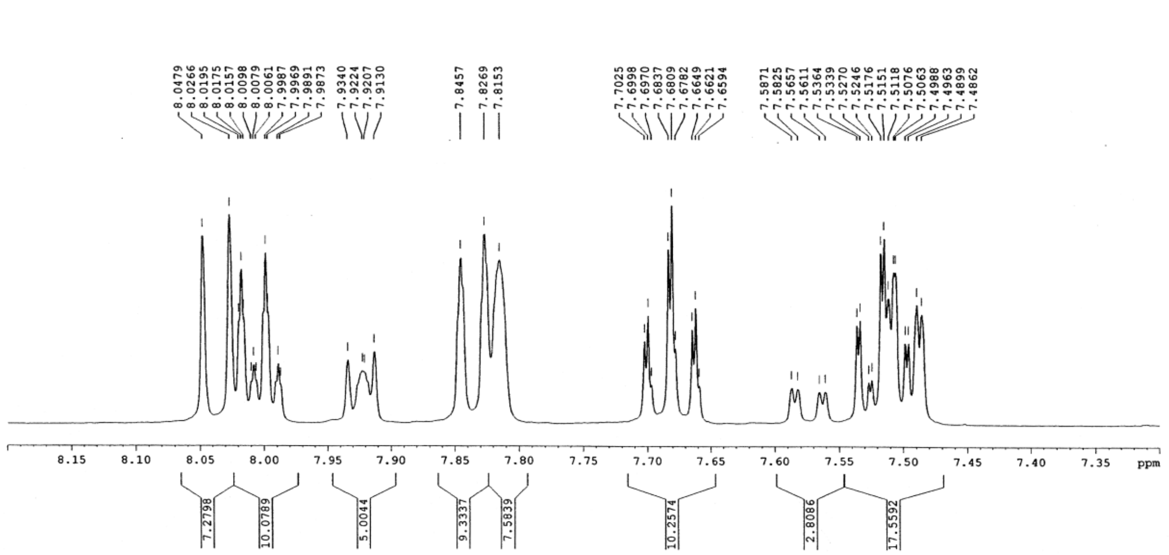

^{13}C spectrum of **IN5**

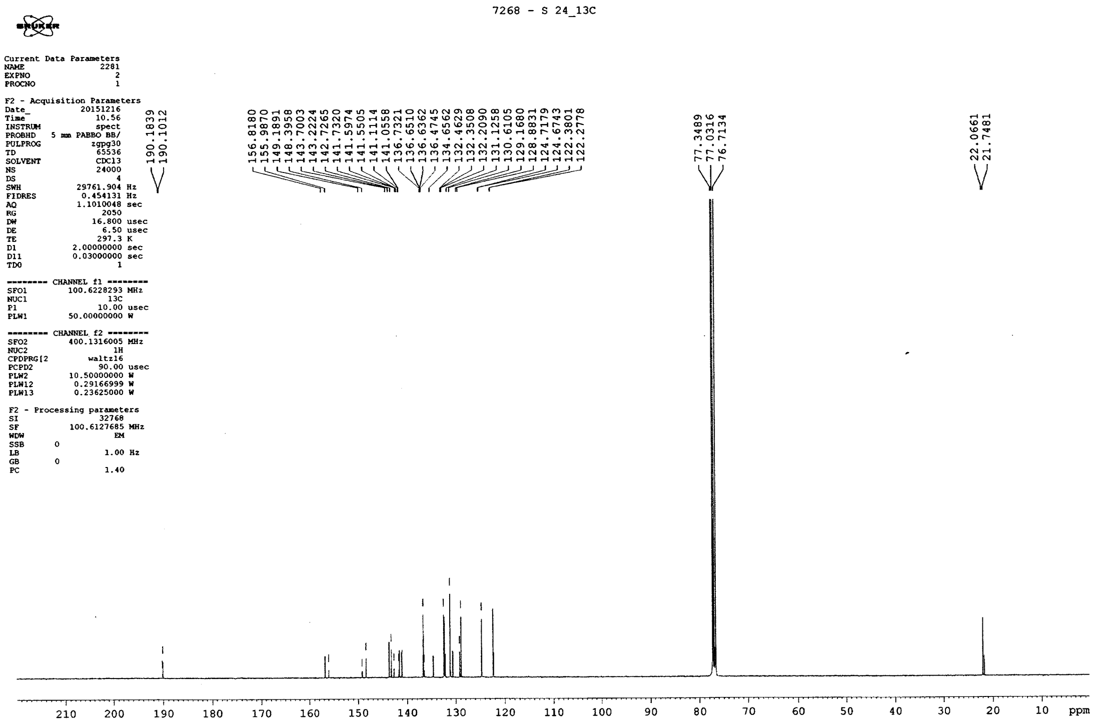

Enlarged spectrum in the range of 120-160 ppm.

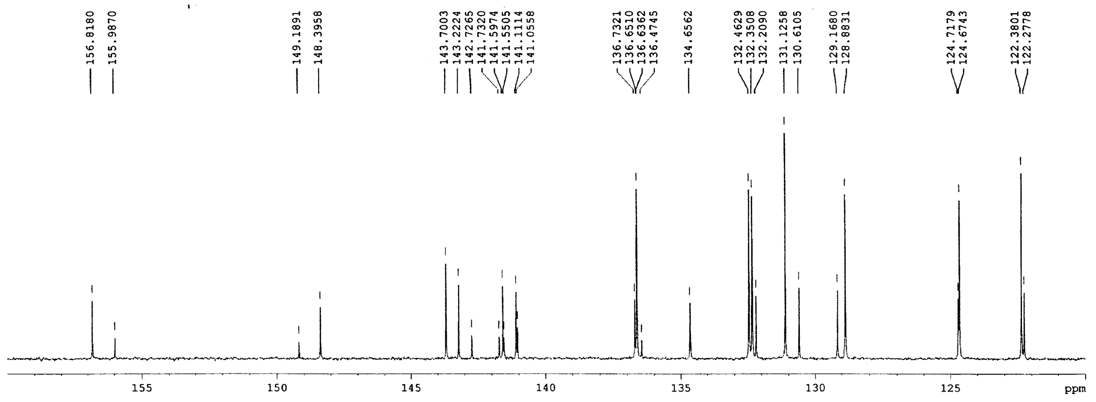

^{1}H spectrum of **IN6**

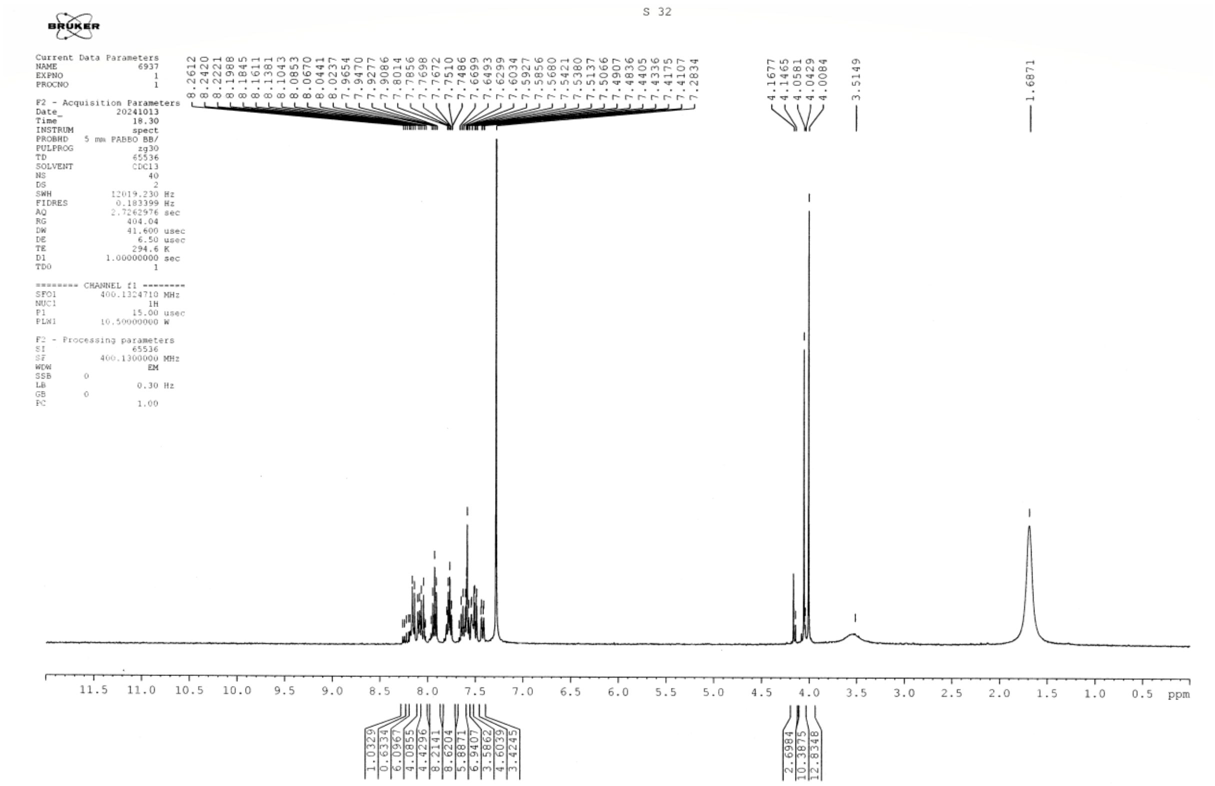

Enlarged spectrum in the range of 7-9 ppm.

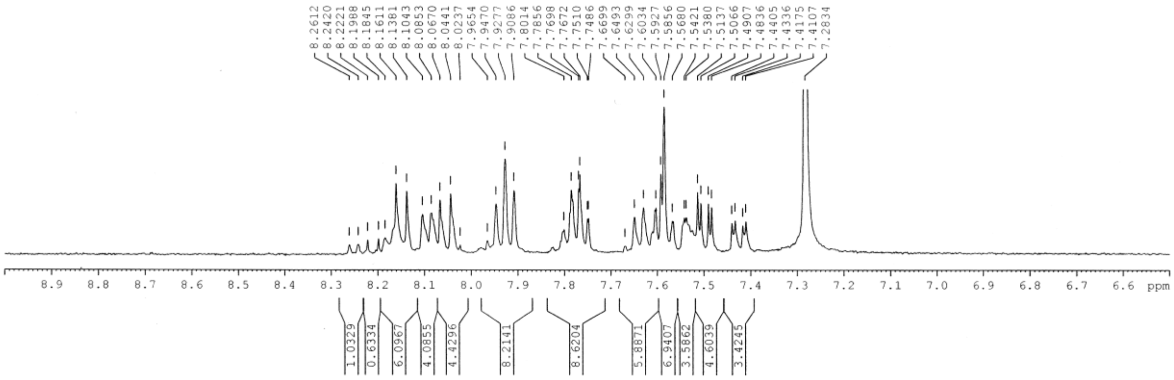

^{13}C spectrum of **IN6**

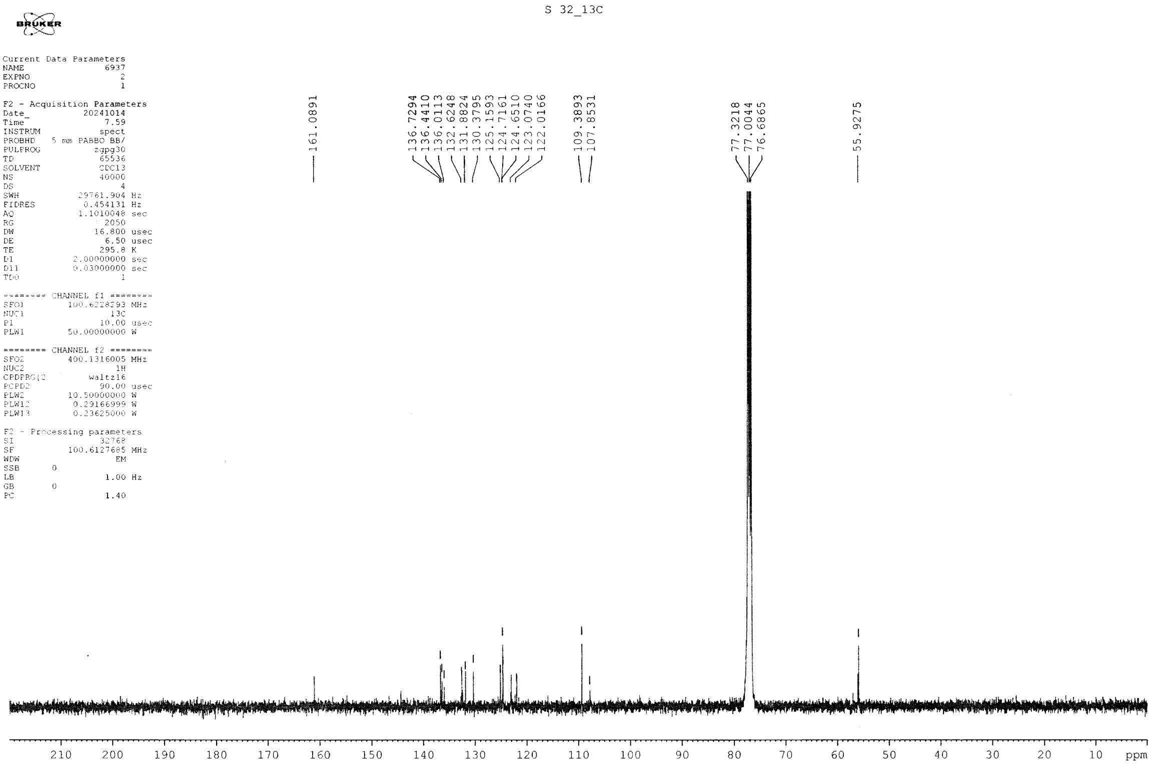

Enlarged spectrum in the range of 100-170 ppm.

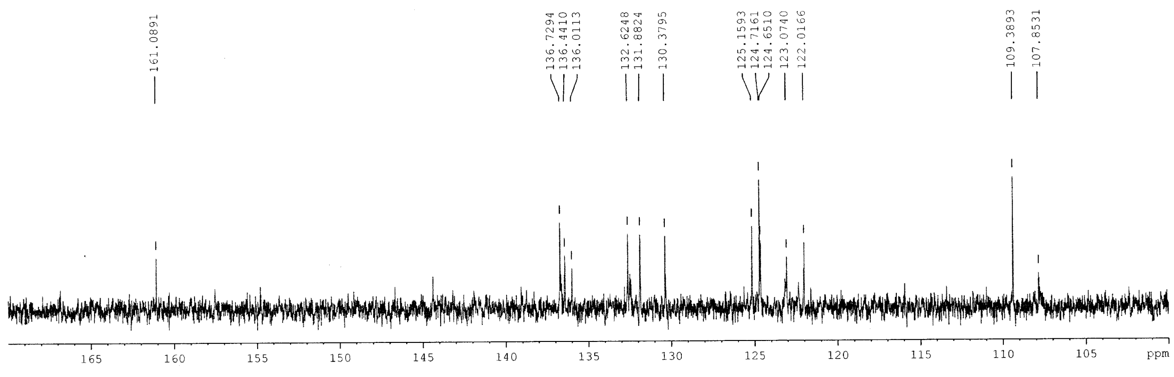

^{1}H spectrum of **IN7**

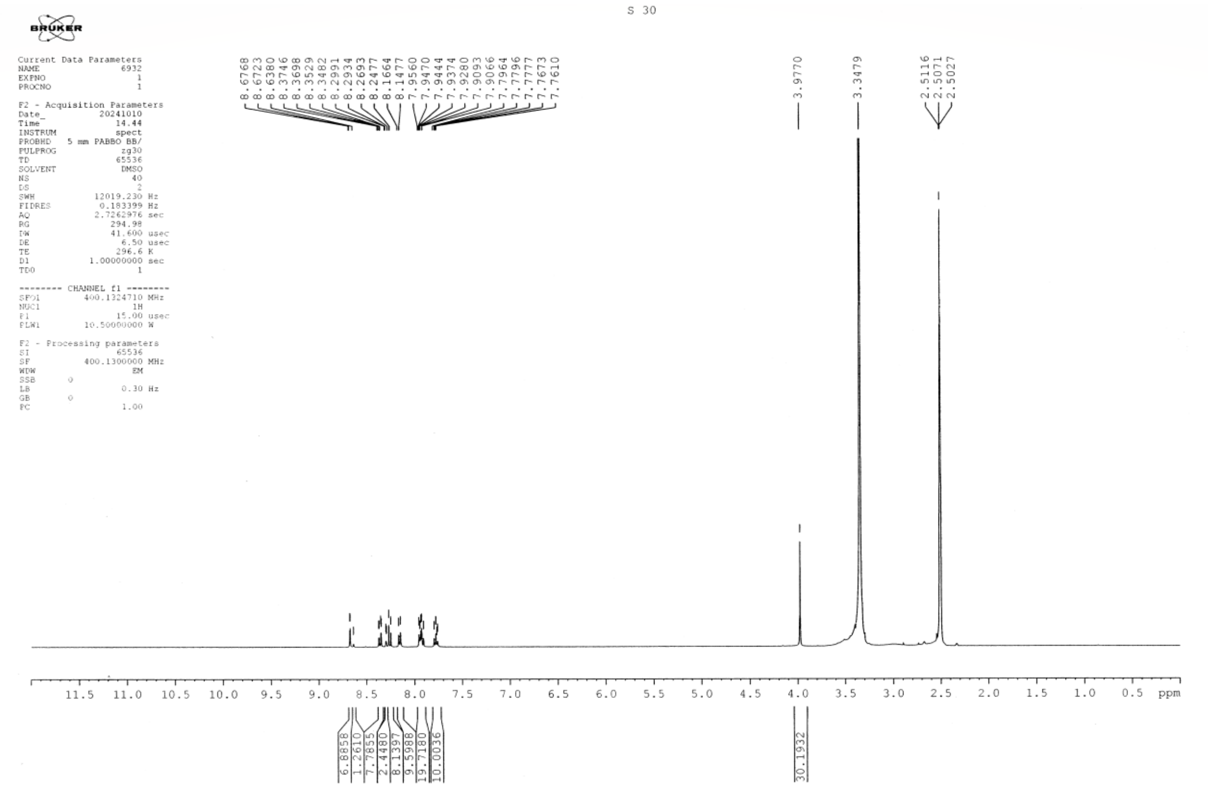

Enlarged spectrum in the range of 7-9 ppm.

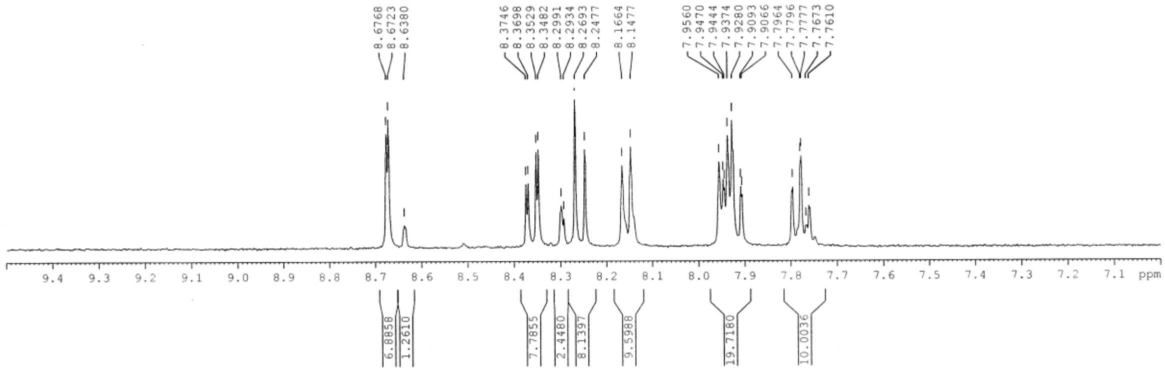

^{13}C spectrum of **IN7**

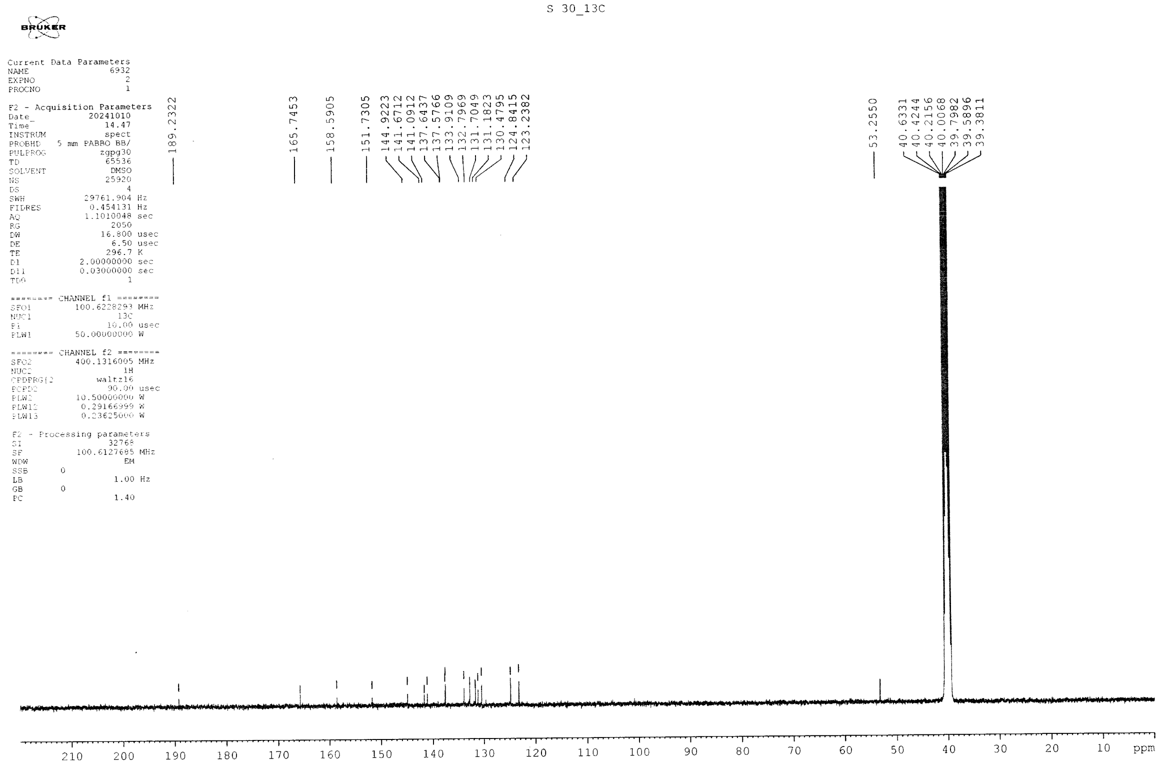

Enlarged spectrum in the range of 10-190 ppm.

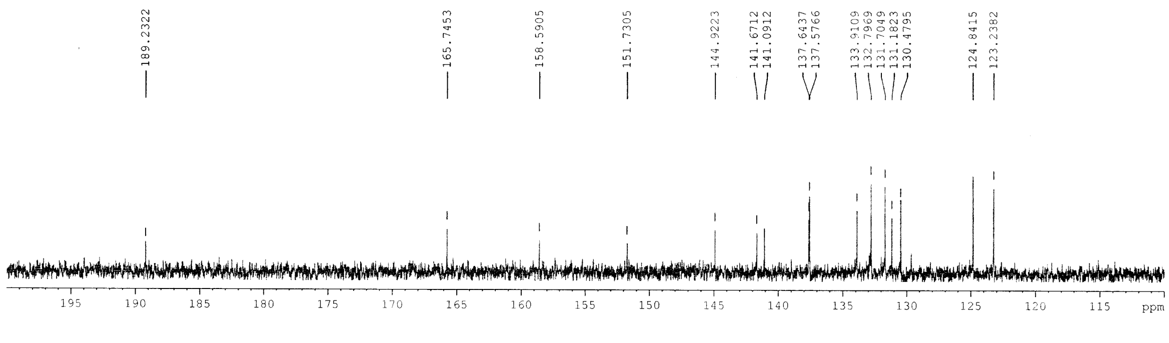

^{1}H spectrum of **IN8**

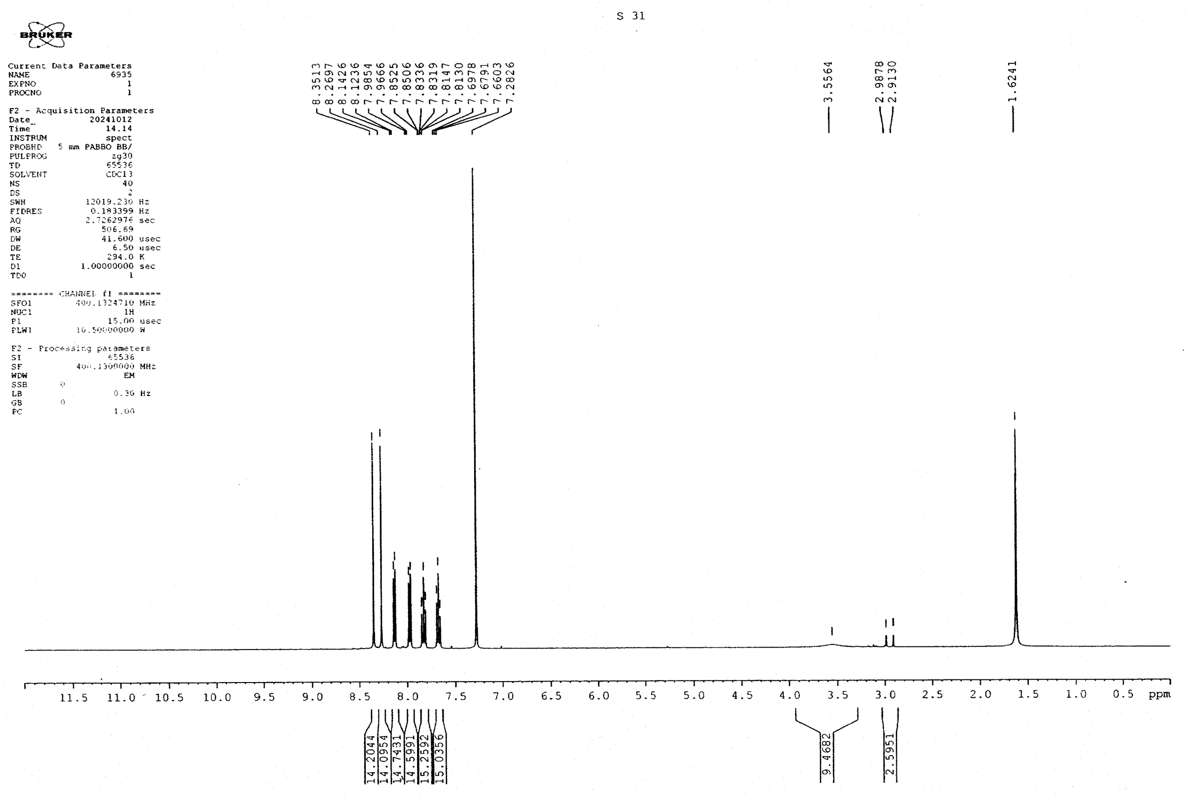

Enlarged spectrum in the range of 7-9 ppm.

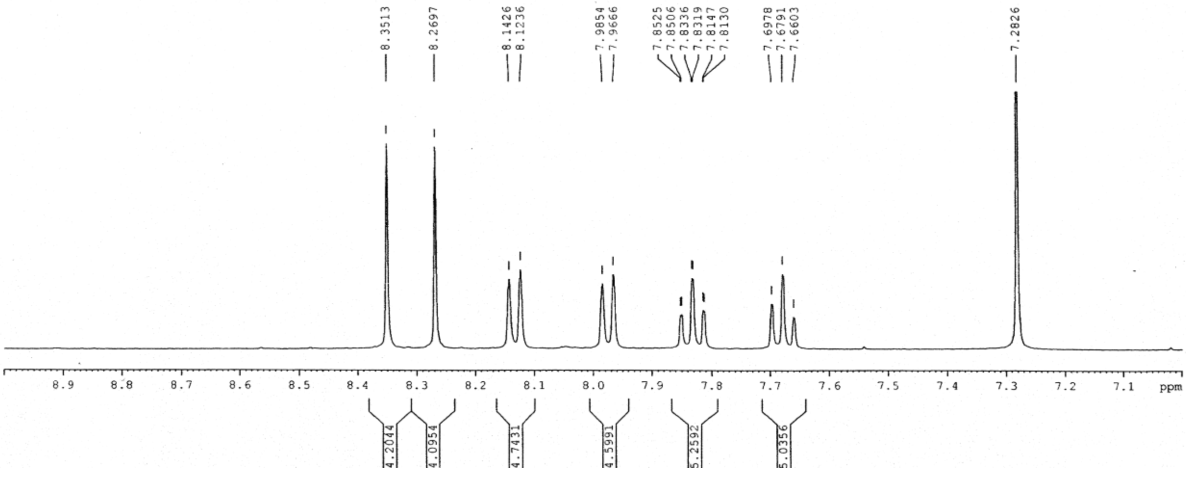

^{13}C spectrum of **IN8**

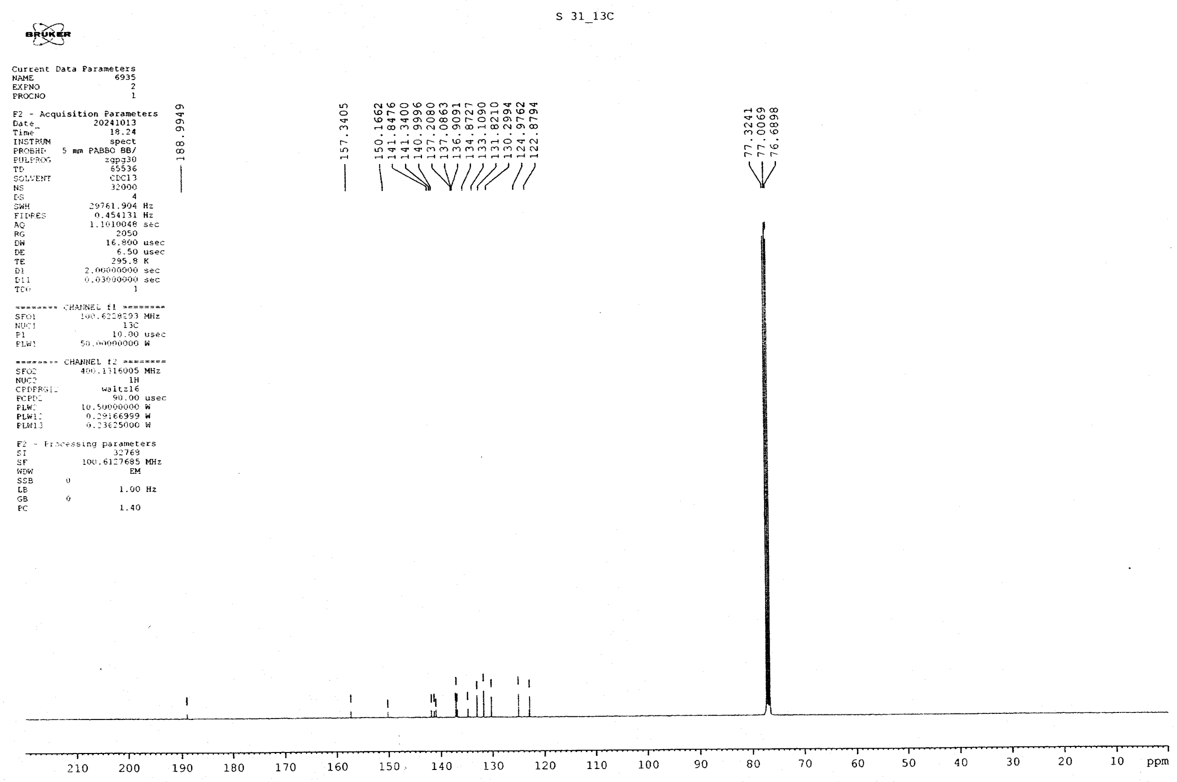

Enlarged spectrum in the range of 115-195 ppm.

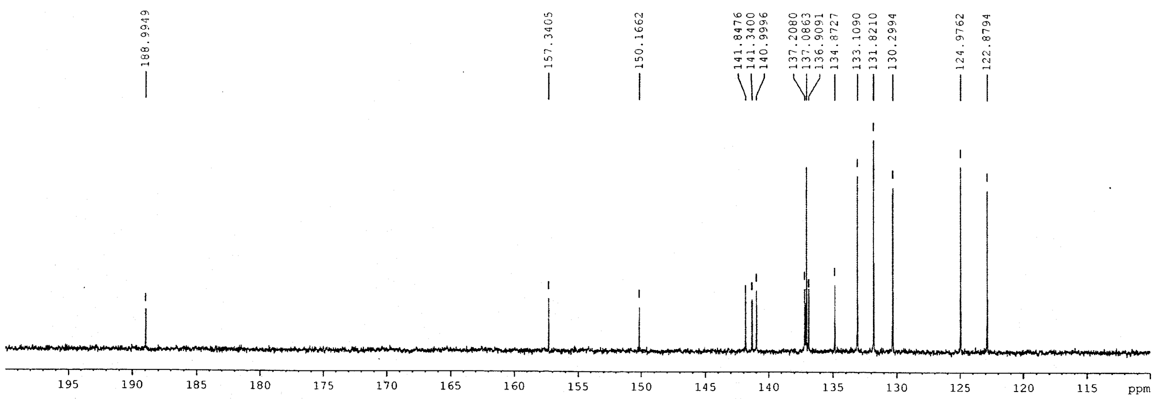

^{1}H spectrum of **IN9**

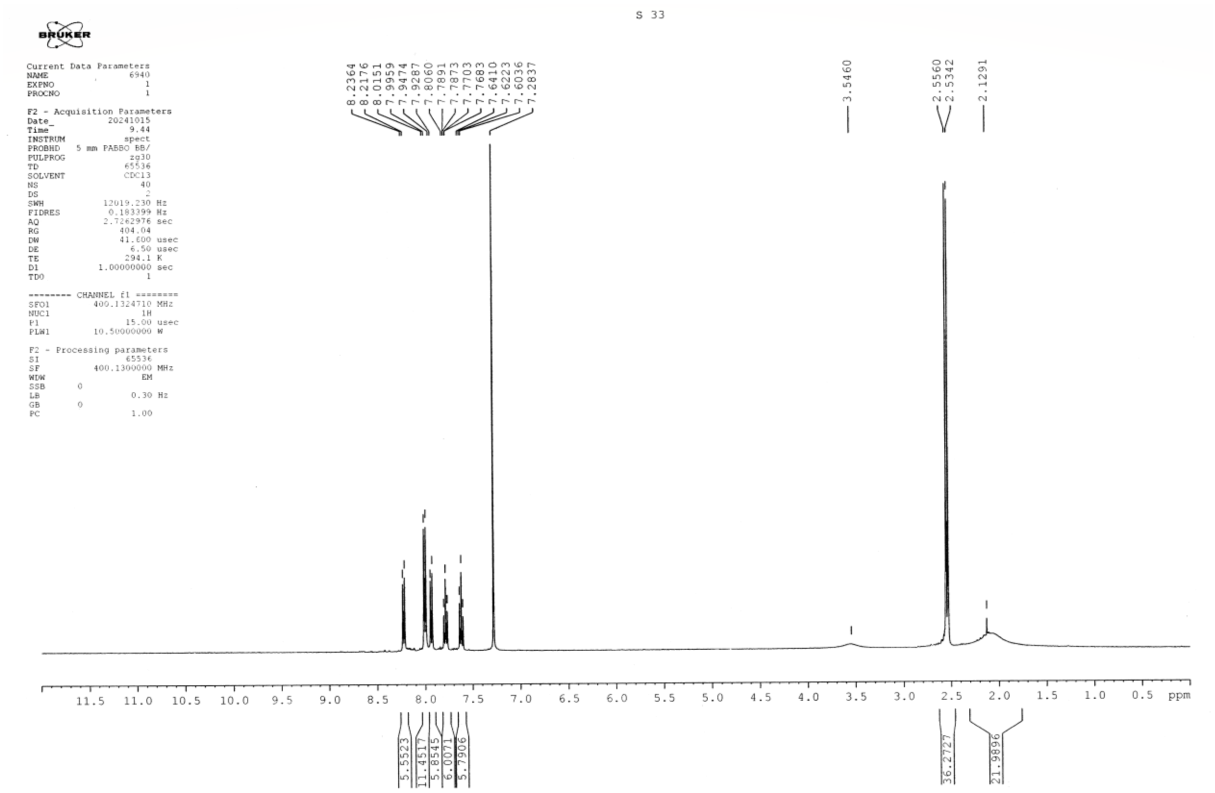

Enlarged spectrum in the range of 7-9 ppm.

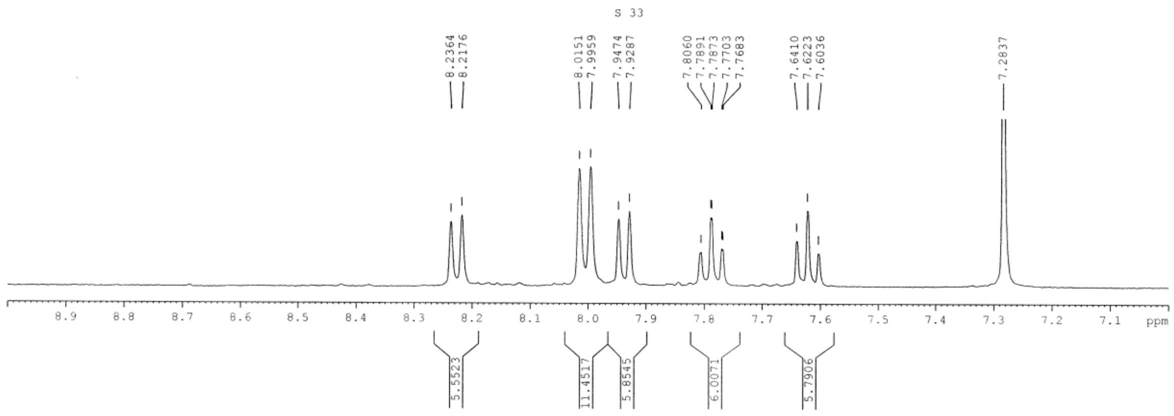

^{13}C spectrum of **IN9**

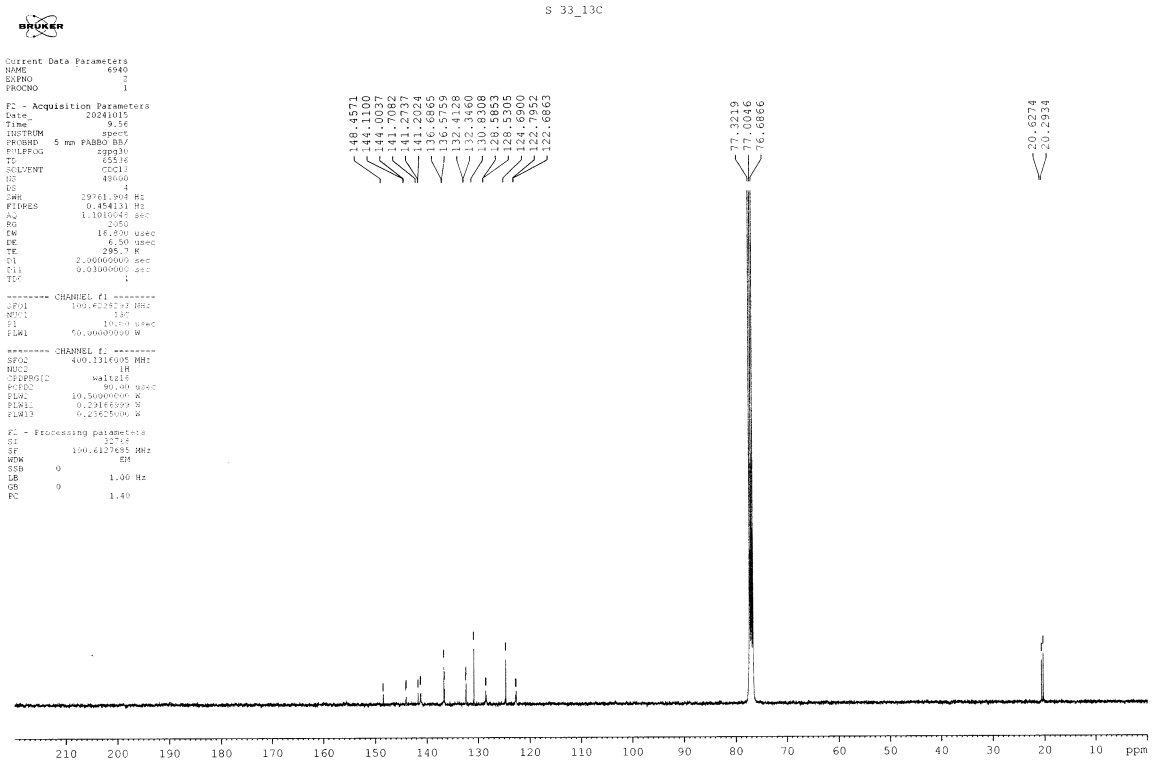

Enlarged spectrum in the range of 15-155 ppm.

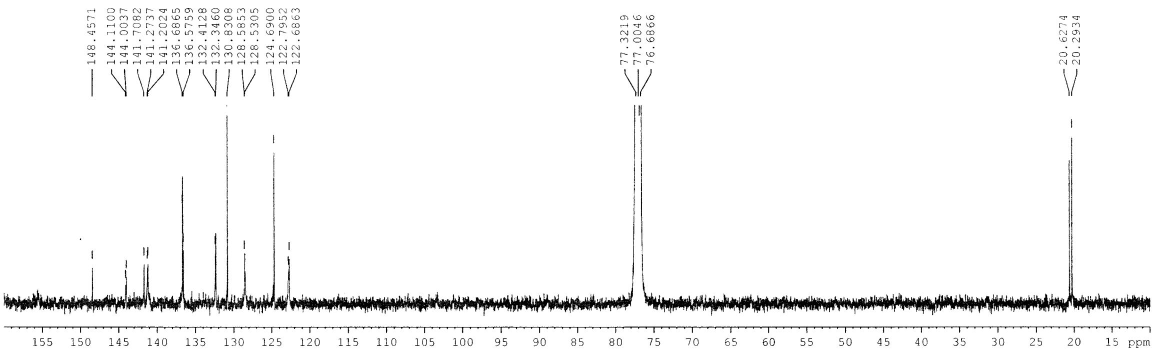

¹H spectrum of **IN10**

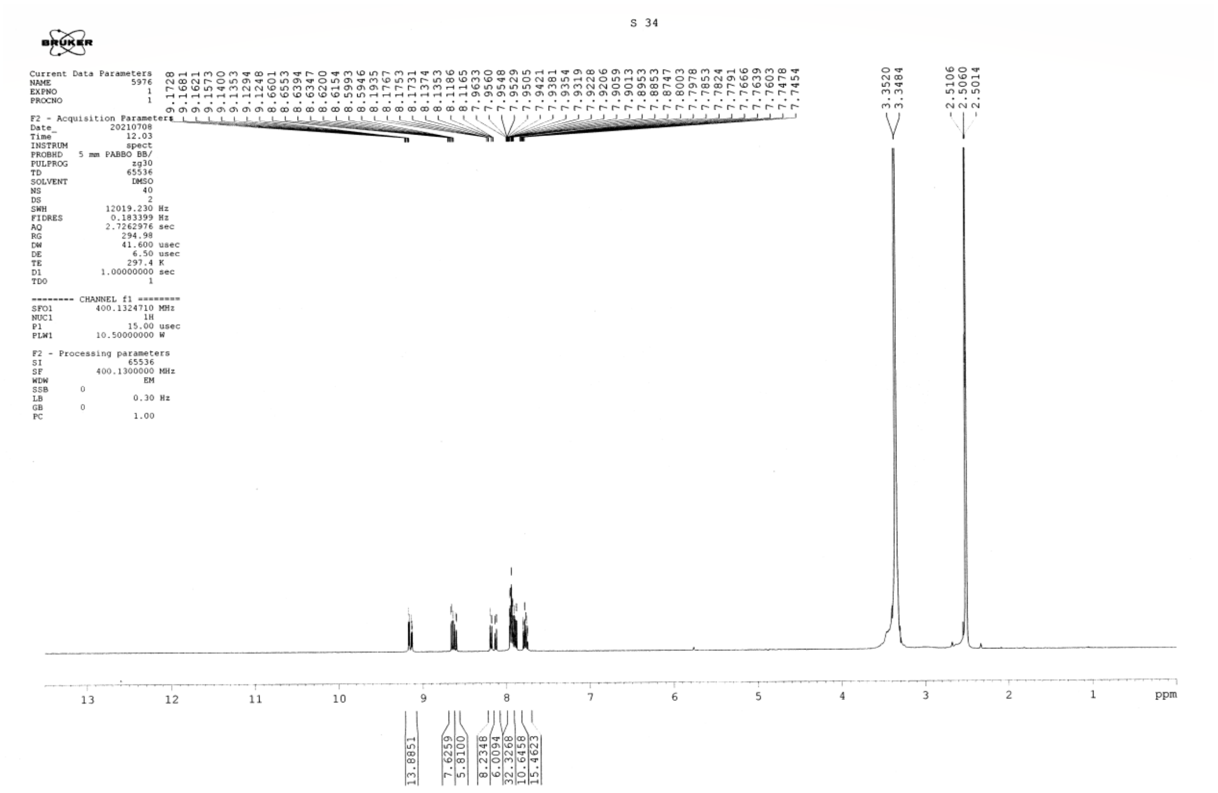

Enlarged spectrum in the range of 7-10 ppm.

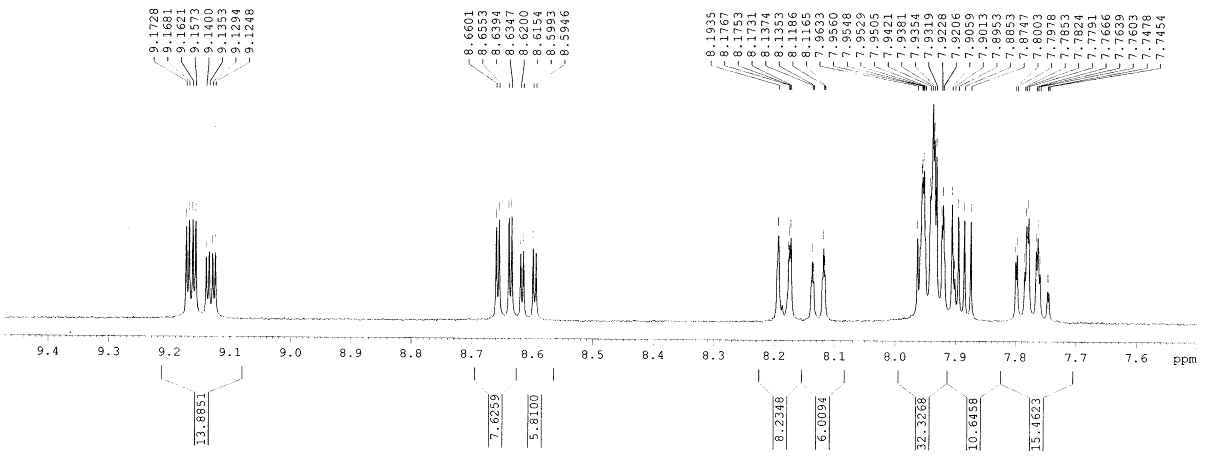

^{13}C spectrum of **IN10**

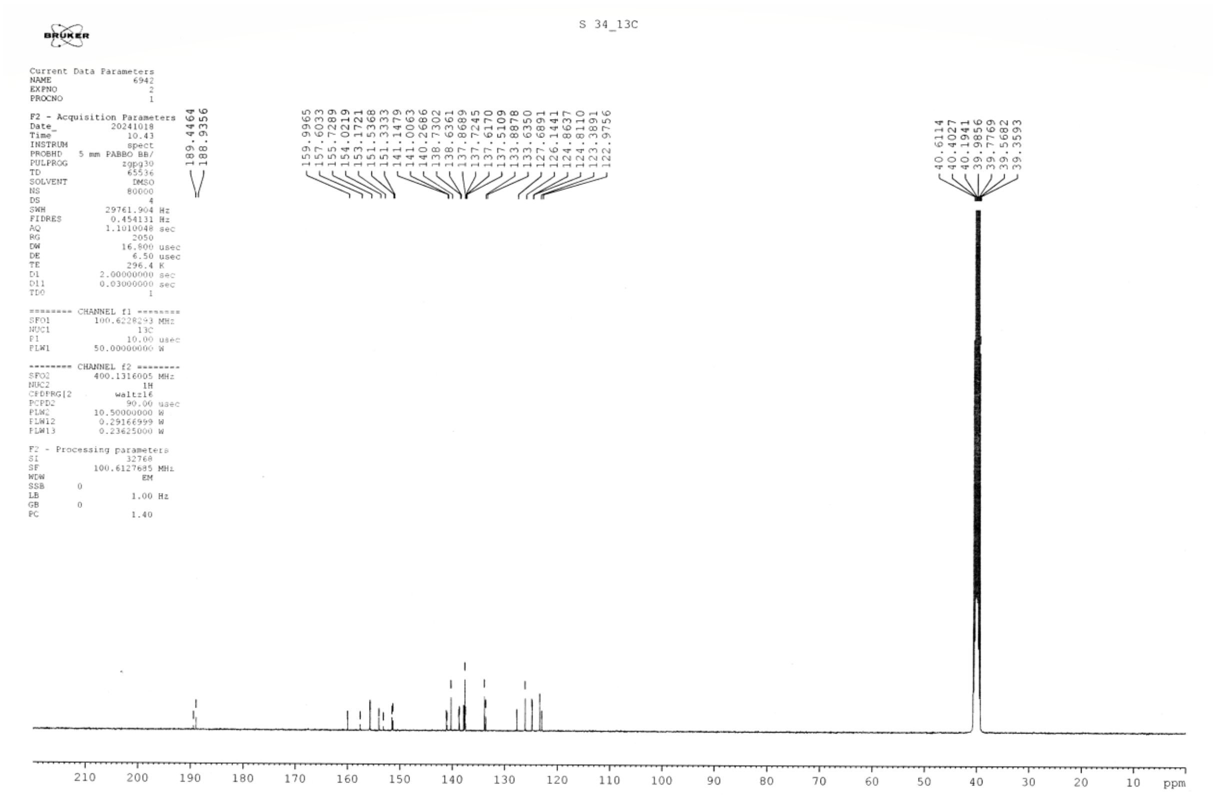

Enlarged spectrum in the range of 120-190 ppm.

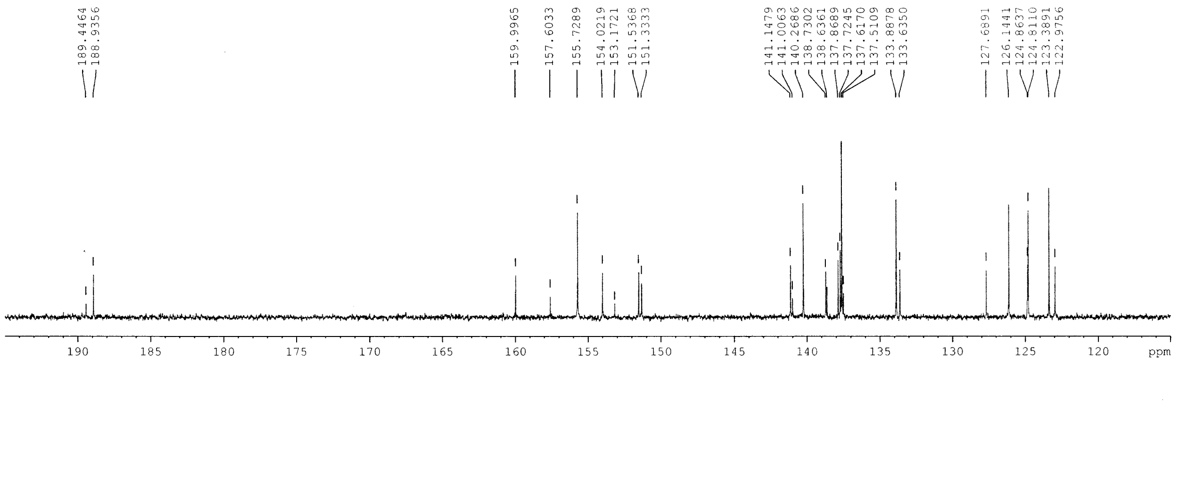

HPLC chromatogram of IN1

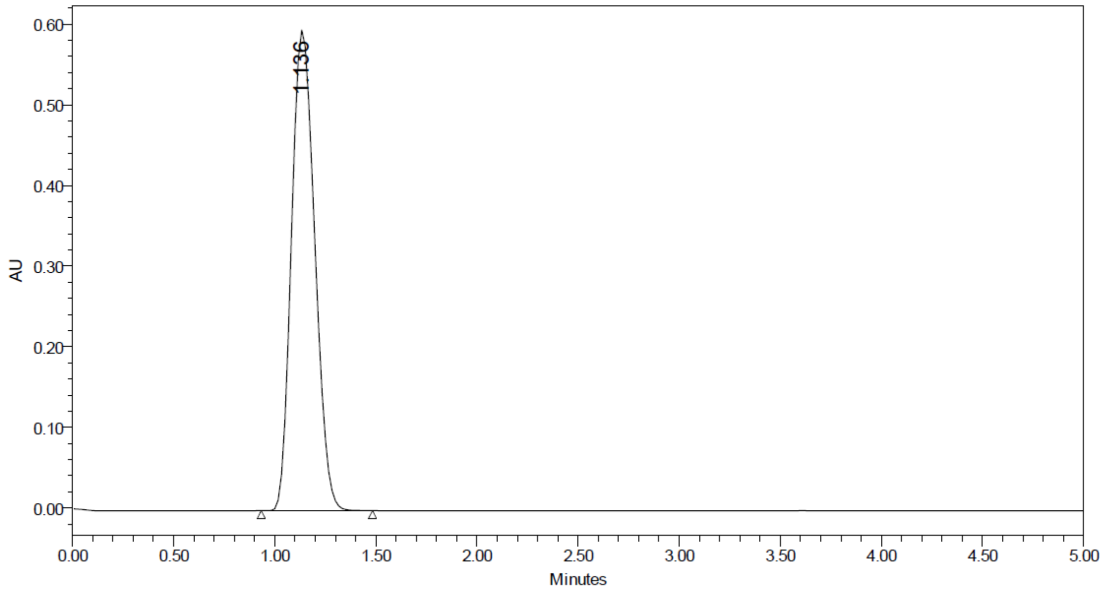

	RT (min)	Peak Type	Area (µV*sec)	% Area	Height (µV)	% Height	Integration Type	Points Across Peak	Start Time (min)	End Time (min)
1	1.136	Unknown	4753030	100.00	595936	100.00	BB	33	0.933	1.483

HPLC chromatogram of IN2

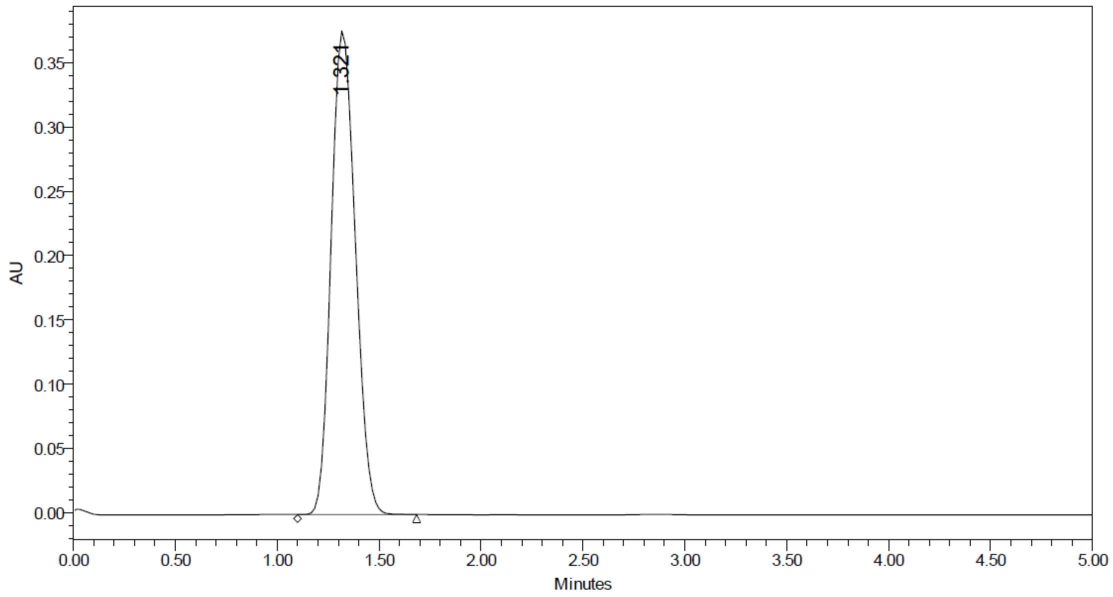

	RT (min)	Peak Type	Area (µV*sec)	% Area	Height (µV)	% Height	Integration Type	Points Across Peak	Start Time (min)	End Time (min)
1	1.321	Unknown	3106958	100.00	376606	100.00	VB	35	1.100	1.683

HPLC chromatogram of IN3

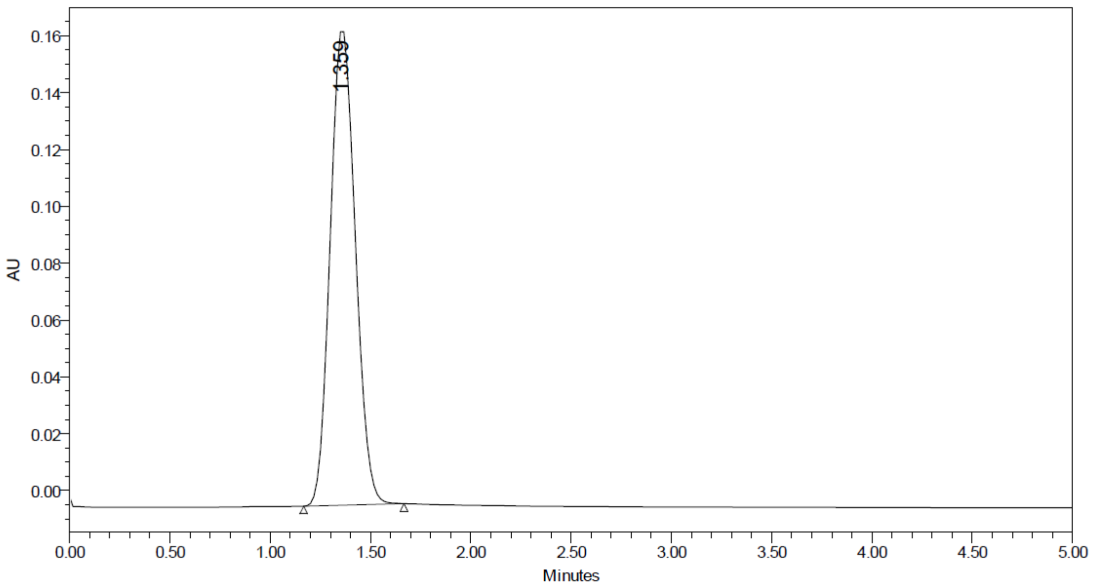

	RT (min)	Peak Type	Area (µV*sec)	% Area	Height (µV)	% Height	Integration Type	Points Across Peak	Start Time (min)	End Time (min)
1	1.359	Unknown	1453241	100.00	168025	100.00	BB	30	1.167	1.667

HPLC chromatogram of IN4

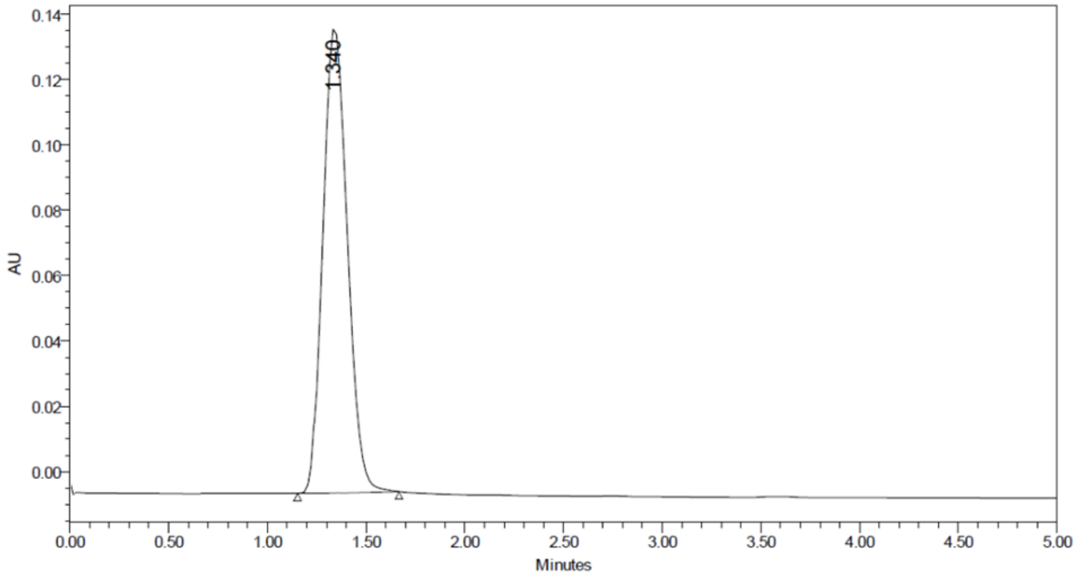

	RT (min)	Peak Type	Area (µV*sec)	% Area	Height (µV)	% Height	Integration Type	Points Across Peak	Start Time (min)	End Time (min)
1	1.340	Unknown	1412770	100.00	142660	100.00	bb	32	1.150	1.667

HPLC chromatogram of IN5

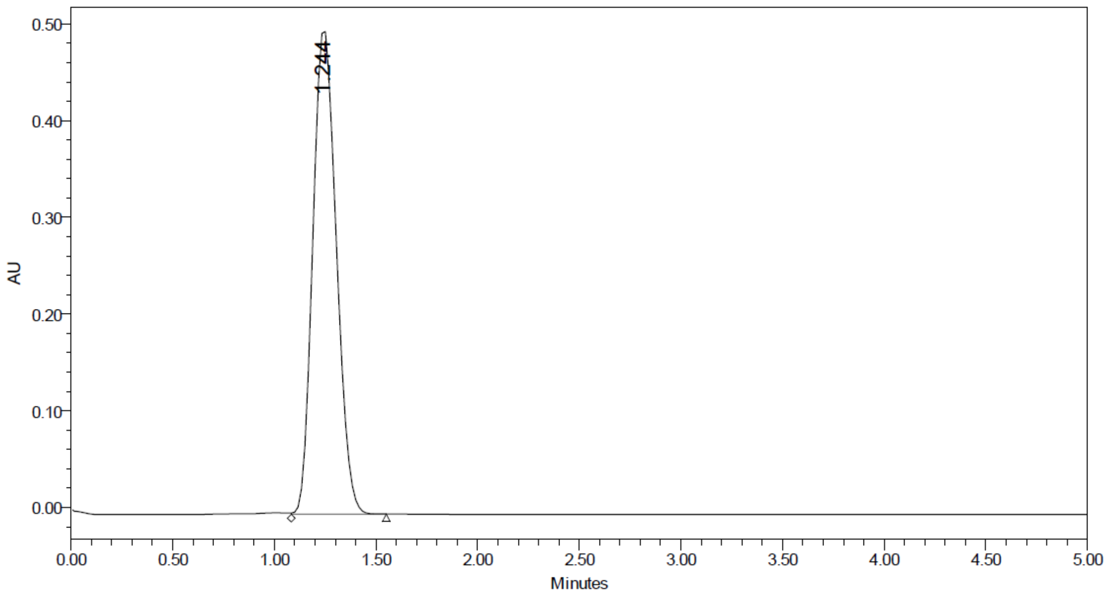

	RT (min)	Peak Type	Area (µV*sec)	% Area	Height (µV)	% Height	Integration Type	Points Across Peak	Start Time (min)	End Time (min)
1	1.244	Unknown	4036669	100.00	501221	100.00	VB	28	1.083	1.550

HPLC chromatogram of IN6

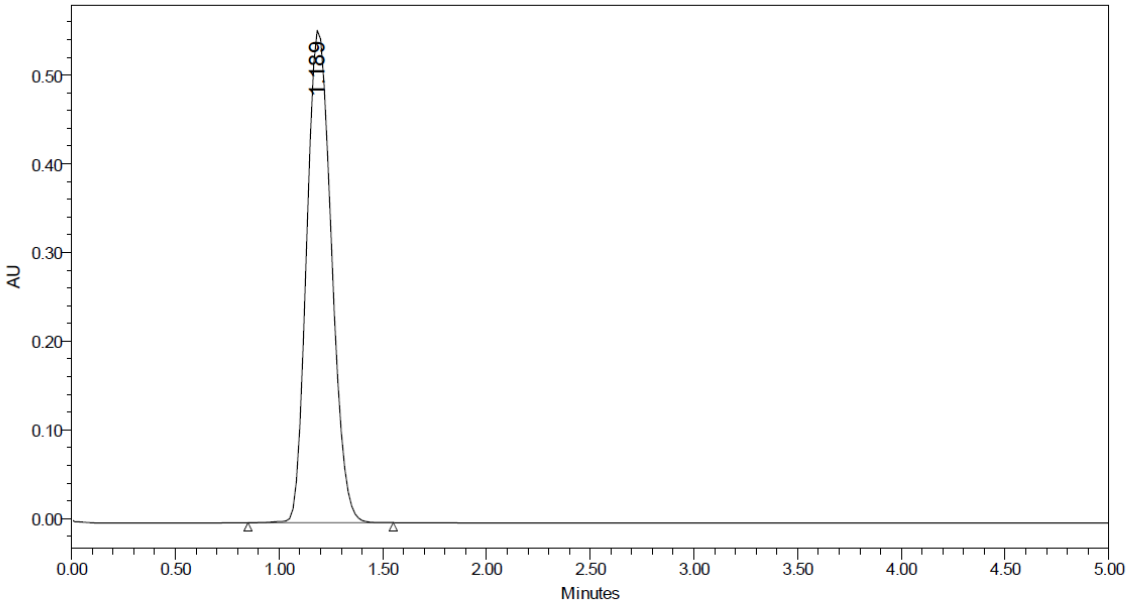

	RT (min)	Peak Type	Area (µV*sec)	% Area	Height (µV)	% Height	Integration Type	Points Across Peak	Start Time (min)	End Time (min)
1	1.189	Unknown	4621696	100.00	556830	100.00	BB	42	0.850	1.550

HPLC chromatogram of IN7

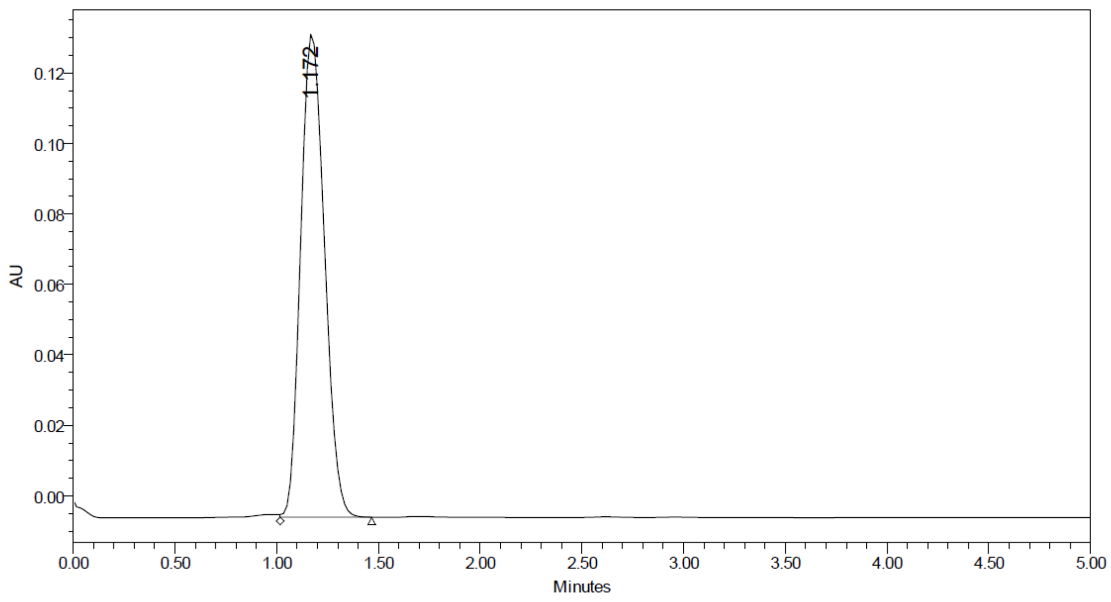

	RT (min)	Peak Type	Area (µV*sec)	% Area	Height (µV)	% Height	Integration Type	Points Across Peak	Start Time (min)	End Time (min)
1	1.172	Unknown	1112653	100.00	137361	100.00	VB	27	1.017	1.467

HPLC chromatogram of IN8

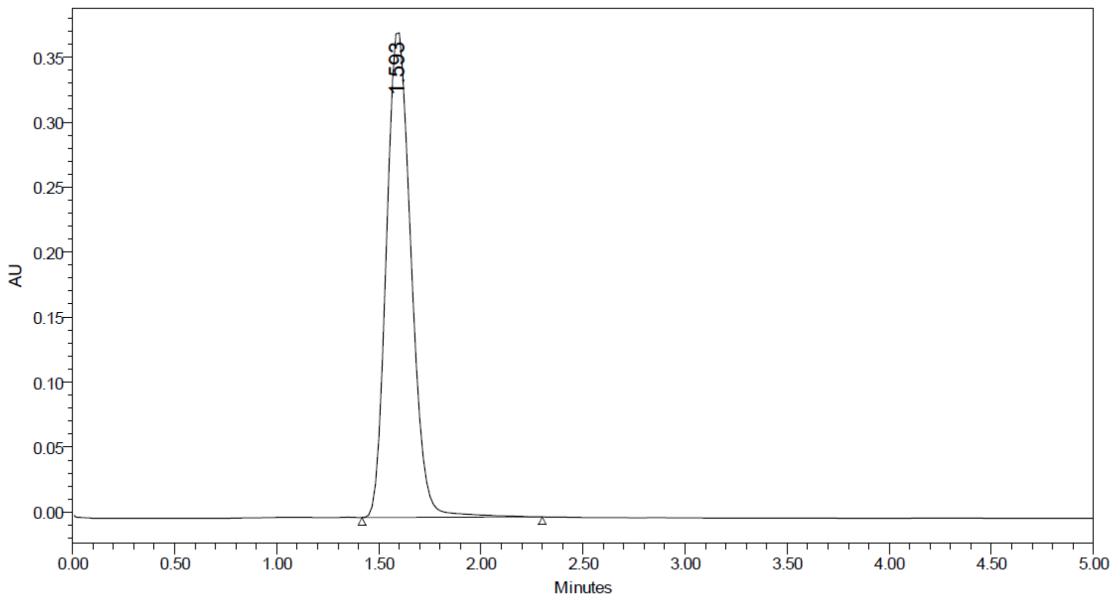

	RT (min)	Peak Type	Area (µV*sec)	% Area	Height (µV)	% Height	Integration Type	Points Across Peak	Start Time (min)	End Time (min)
1	1.593	Unknown	3151749	100.00	374779	100.00	BB	53	1.417	2.300

HPLC chromatogram of IN9

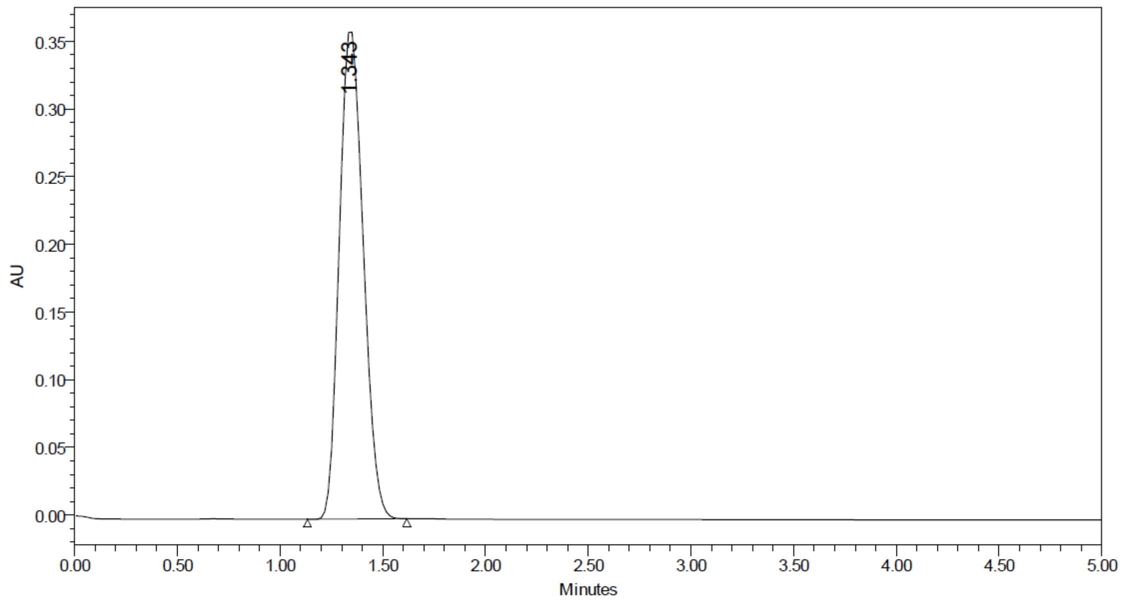

	RT (min)	Peak Type	Area (µV*sec)	% Area	Height (µV)	% Height	Integration Type	Points Across Peak	Start Time (min)	End Time (min)
1	1.343	Unknown	2918773	100.00	361957	100.00	BB	29	1.133	1.617

HPLC chromatogram of IN10

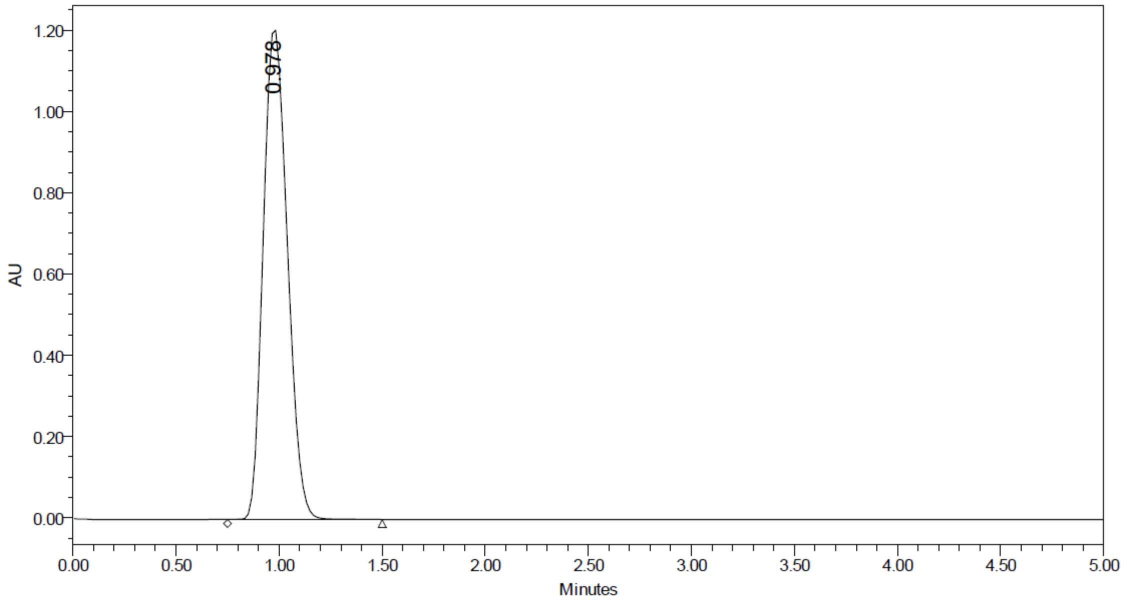

	RT (min)	Peak Type	Area (µV*sec)	% Area	Height (µV)	% Height	Integration Type	Points Across Peak	Start Time (min)	End Time (min)
1	0.978	Unknown	9801018	100.00	1208187	100.00	VB	45	0.750	1.500

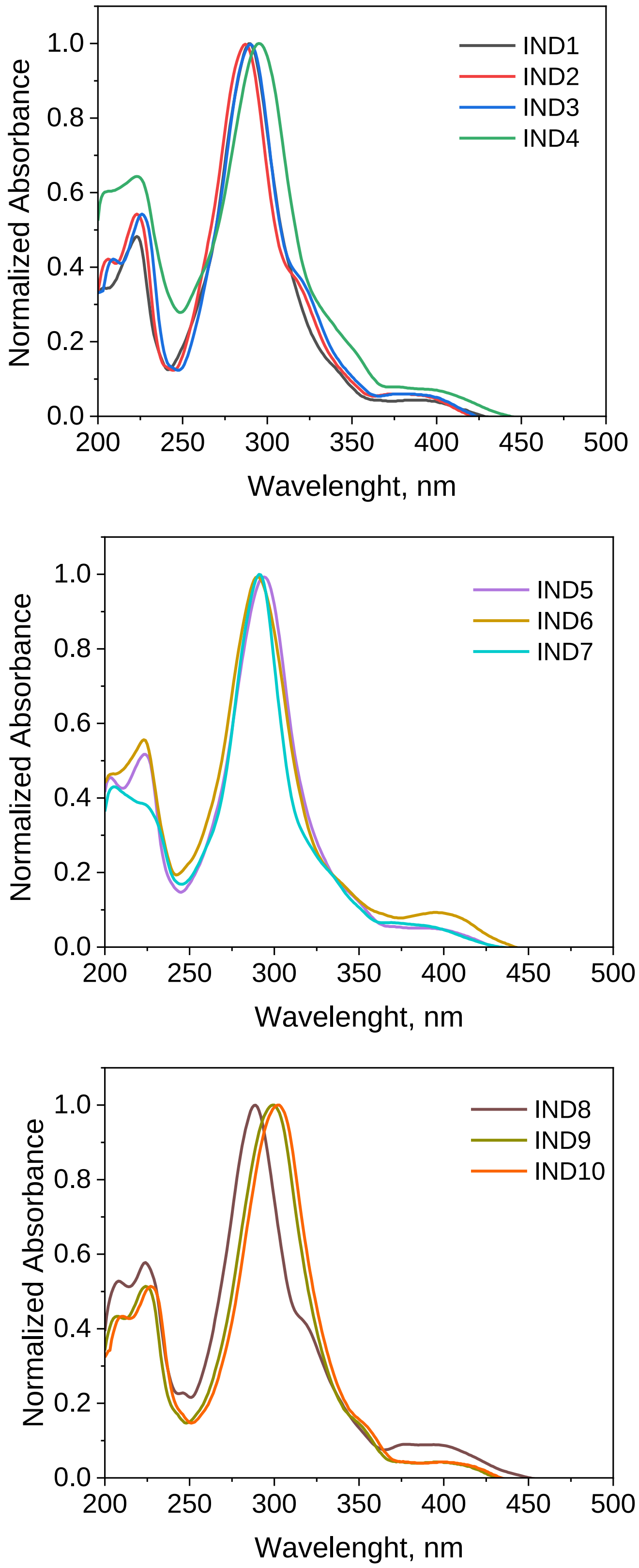

Figure S1. Normalized electronic absorption spectra of synthesized IND1-IND10 in ethyl acetate.

Table S1. Average values of solubility, sorption and mass change for the samples tested in distilled water, 3% acetic acid solution, artificial saliva, *n*-heptane and coffee.

Parametr	Storage time, dyes	Solution				
		distilled water	3% acetic acid	artificial saliva	*n*-heptane	coffee
**IND1**						
S_l, %	7	0.1007±0.05	5.8062±0.21	0.5842±0.23	2.3106±0.34	0.2250±0.21
	14	0.4279±0.15	13.1251±0.35	1.2369±0.31	4.5699±0.28	0.2750±0.32
	21	0.7552±0.19	18.9819±0.27	1.9895±0.29	9.4223±0.49	0.3250±0.41
	28	0.8308±0.17	26.5689±0.22	2.1895±0.35	13.2220±0.35	0.3500±0.21
S_p, %	7	3.10134±0.24	4.5016±0.27	3.5605±0.27	0.0525±0.04	4.9761±0.13
	14	3.11122±0.28	6.0670±0.32	3.5623±0.26	0.0537±0.08	5.2719±0.18
	21	3.24005±0.25	6.3869±0.35	3.5641±0.31	0.0566±0.09	5.4092±0.27
	28	3.2662±0.29	6.7476±0.38	3.5886±0.42	0.0591±0.11	5.5002±0.29
D_m, %	7	2.7693±0.33	-1.3661±0.11	3.5009±0.39	-2.2593±0.27	5.0000±0.05
	14	2.6693±0.34	-7.5140±0.23	3.4482±0.27	-4.5186±0.26	5.2750±0.05
	21	2.5679±0.52	-20.9319±0.22	3.3956±0.31	-9.3709±0.36	5.3750±0.05
	28	2.5176±0.48	-27.6897±0.38	3.3919±0.48	-13.1707±0.98	5.4500±0.05
**IND2**						
S_l, %	7	0.3303±0.21	4.1015±0.31	0.48708±0.07	2.2145±0.27	0.1968±0.24
	14	0.5082±0.18	10.0611±0.47	1.2588±0.21	4.6591±0.23	0.2707±0.26
	21	0.7623±0.24	13.6868±0.38	1.9466±0.32	8.0241±0.25	0.2953±0.54
	28	1.1181±0.30	21.6406±0.17	2.1327±0.38	10.8426±0.11	0.2953±0.08
S_p, %	7	3.7546±0.28	4.9415±0.23	3.4961±0.41	0.0881±0.63	4.6106±0.21
	14	3.7847±0.23	5.5449±0.09	3.5209±0.24	0.0904±0.04	4.9272±0.22
	21	3.7940±0.12	5.6958±0.52	3.5449±0.23	0.0937±0.23	5.1510±0.48
	28	3.9496±0.19	5.8022±0.45	3.5664±0.22	0.0966±0.08	5.3283±0.98
D_m, %	7	3.5578±0.31	0.8837±0.60	3.32535±0.20	-2.1282±0.61	4.6271±0.27
	14	3.4053±0.09	-4.7813±0.49	3.2775±0.08	-4.5729±0.14	4.8978±0.34
	21	3.1512±0.25	-8.8601±0.31	3.2535±0.18	-7.9378±0.19	5.1193±0.56
	28	2.9479±0.18	-16.8139±0.27	3.2514±0.34	-10.7563±0.71	5.3162±0.23
**IND3**						
S_l, %	7	0.0774±0.24	0.0759±0.05	0.6202±0.14	2.4364±0.20	0.2502±0.24
	14	0.1416±0.19	7.7682±0.15	1.3355±0.21	6.1689±0.13	0.2703±0.17
	21	0.1548±0.36	14.7520±0.22	1.9198±0.27	9.4349±0.10	0.2949±0.39
	28	0.2580±0.32	26.6447±0.34	2.0589±0.28	11.3271±0.33	0.2949±0.24
S_p, %	7	2.7624±0.20	5.15147±0.21	4.0893±0.17	0.0304±0.42	4.6849±0.20
	14	2.7512±0.18	5.9354±0.26	4.0584±0.19	0.0552±0.17	5.0093±0.11
	21	2.7645±0.27	6.5205±0.78	4.1338±0.43	0.0572±0.19	5.1659±0.04
	28	2.8406±0.35	6.8145±0.65	4.1916±0.38	0.0584±0.24	5.2324±0.25
D_m, %	7	2.7612±0.43	0.2277±0.57	3.2554±0.32	-2.7475±0.28	4.9152±0.32
	14	2.7096±0.17	-1.9483±0.08	2.8380±0.23	-6.1171±0.11	4.9889±0.38
	21	2.6838±0.24	-8.8056±0.31	2.3094±0.25	-9.3831±0.23	5.1363±0.16
	28	2.4980±0.61	-21.2803±0.18	2.2259±0.09	-11.2752±0.19	5.2101±0.23

			**IND4**			
S_l, %	7	0.2473±0.27	4.0310±0.29	0.5238±0.25	2.4036±0.57	0.15394±0.49
	14	0.5194±0.32	9.4949±0.13	1.4218±0.37	5.1642±0.25	0.1979±0.38
	21	0.7667±0.17	16.1728±0.14	1.9456±0.15	9.0433±0.14	0.2199±0.45
	28	1.4345±0.09	22.6080±0.61	2.120±0.34	10.6377±0.28	0.24191±0.42
S_p, %	7	3.2729±0.23	5.5921±0.18	4.1123±0.39	0.0731±0.33	5.4845±0.27
	14	3.3173±0.35	5.6933±0.31	4.2867±0.13	0.0752±0.39	5.5567±0.33
	21	3.3485±0.09	6.9792±0.30	4.4714±0.54	0.0784±0.47	5.7344±0.48
	28	3.5342±0.21	7.0574±0.52	4.5023±0.23	0.0798±0.56	5.8335±0.21
D_m, %	7	3.017±0.15	1.3355±0.50	3.5669±0.13	-2.3322±0.19	5.3221±0.18
	14	2.8938±0.07	-4.0310±0.32	2.9932±0.87	-5.092±0.27	5.6740±0.67
	21	2.6712±0.13	-9.8834±0.17	2.6440±0.63	-8.9719±0.39	5.8500±0.32
	28	2.5366±0.54	-16.7314±0.62	2.4943±0.22	-10.5663±0.45	5.9379±0.22
			**IND5**			
S_l, %	7	0.1426±0.27	0.1902±0.22	0.6384±0.09	3.5298±0.51	0.1973±0.52
	14	0.1902±0.58	9.7470±0.48	1.2153±0.12	4.4717±0.60	0.2659±0.31
	21	0.3328±0.56	15.5753±0.42	1.8524±0.13	8.4766±0.58	0.3261±0.54
	28	0.4517±0.98	25.2480±0.40	2.1464±0.25	9.3611±0.49	0.3561±0.63
S_p, %	7	4.3498±0.91	4.4785±0.99	3.4112±0.18	0.0414±0.89	5.6334±0.59
	14	4.3735±0.78	5.3083±0.10	3.6418±0.10	0.0771±0.25	5.5137±0.48
	21	4.4667±0.67	5.6279±0.09	3.8882±0.26	0.0804±0.38	5.6651±0.27
	28	4.5153±0.25	5.9887±0.02	3.9439±0.38	0.0812±0.05	5.7553±0.35
D_m, %	7	4.3984±0.26	1.0664±0.13	4.9831±0.42	-2.3832±0.20	4.9638±0.64
	14	4.2898±0.49	-9.6478±0.35	4.8189±0.44	-3.5410±0.26	5.3250±0.48
	21	4.1971±0.85	-10.5406±0.90	4.5104±0.49	-8.4029±0.35	5.7808±0.32
	28	4.0517±0.65	-25.2480±1.05	4.3464±0.17	-9.3611±0.82	5.9561±0.44
			**IND6**			
S_l, %	7	-6.3205±0.31	4.2126±0.63	0.5580±0.16	1.9264±0.33	0.1116±0.40
	14	-6.4858±0.24	11.1977±0.49	1.2092±0.35	2.2752±0.20	0.2418±0.29
	21	-6.3378±0.35	16.7493±0.97	2.4186±0.29	3.8028±0.15	0.4837±0.33
	28	-6.2145±0.18	25.9626±1.09	2.9766±0.69	4.7035±0.05	0.5932±0.56
S_p, %	7	3.6471±0.26	4.9734±0.18	2.1753±0.72	0.07235±0.13	4.4840±0.48
	14	4.2572±0.35	5.1476±0.29	3.5432±0.26	0.0747±0.48	5.3140±0.28
	21	4.3478±0.65	5.4467±0.32	3.7825±0.29	0.0779±0.63	5.4600±0.64
	28	4.3951±0.93	5.7726±0.53	3.8365±0.36	0.0786±0.21	5.5471±0.97
D_m, %	7	3.4525±0.14	3.2837±0.62	3.9069±0.43	3.1523±0.16	4.4943±0.90
	14	3.22±0.30	-6.3784±0.45	3.0465±0.19	0.3502±0.09	5.8223±0.87
	21	3.1713±0.26	-11.9534±0.39	2.67441±0.31	-3.7277±0.38	5.9506±0.70
	28	3.0974±0.85	-21.4268±1.05	2.4418±0.24	-4.6284±0.19	5.9928±0.61
			**IND7**			
S_l, %	7	-6.3936±0.13	4.2081±0.32	0.5466±0.25	1.8840±0.31	0.1093±0.23
	14	-6.3449±0.56	10.9646±0.28	1.1844±0.54	2.2691±0.54	0.2368±0.35
	21	-6.2002±0.39	16.4006±0.65	2.3690±0.42	3.7191±0.40	0.4738±0.46
	28	-6.0796±0.72	25.4221±0.49	2.9156±0.32	4.5999±0.28	0.5831±0.44

S_p, %	7	3.5705±0.81	4.9134±0.18	3.1515±0.25	0.0818±0.69	4.3936±0.31
	14	4.1739±0.25	5.0333±0.34	3.4726±0.28	0.0731±0.26	5.1734±0.29
	21	4.2627±0.56	5.3183±0.42	3.7069±0.36	0.0761±0.30	5.3157±0.22
	28	4.3090±0.32	5.6206±0.52	3.7597±0.39	0.0768±0.32	5.4006±0.35
D_m, %	7	2.9775±0.41	-3.2153±0.50	2.8883±0.16	3.0829±0.41	5.2667±0.38
	14	2.877±0.27	-6.2456±0.63	2.884±0.28	0.3420±0.50	5.3139±0.19
	21	2.6288±0.56	-11.7048±0.32	2.619±0.30	-3.6457±0.71	5.3838±0.25
	28	2.5564±0.42	-20.9808±0.98	2.5917±0.25	-4.5265±0.13	5.3997±0.28
**IND8**						
S_l, %	7	-6.5190±0.20	4.2116±0.25	0.554±0.87	1.9168±0.89	0.1110±0.10
	14	-6.4539±0.38	11.1450±0.27	1.2036±0.52	2.2738±0.63	0.2407±0.15
	21	-6.3067±0.19	16.6705±0.09	2.4074±0.63	3.7839±0.67	0.4814±0.11
	28	-6.1840±0.35	25.8405±1.09	2.9628±0.37	4.6801±0.49	0.5925±0.10
S_p, %	7	3.6298±0.47	5.9599±0.26	3.1699±0.26	0.0898±0.31	4.4636±0.26
	14	4.2384±0.45	5.1217±0.38	3.5272±0.65	0.0744±0.75	5.2821±0.21
	21	4.3286±0.49	5.4176±0.27	3.7655±0.67	0.0775±0.25	5.4272±0.29
	28	4.3756±0.52	5.7381±0.96	3.81920±0.28	0.0782±0.67	5.5139±0.15
D_m, %	7	3.4355±0.63	3.2682±0.83	3.2027±0.45	3.1366±0.66	5.4727±0.17
	14	3.1656±0.70	-6.3484±0.24	3.0324±0.45	0.3485±0.28	5.7232±0.57
	21	3.1165±0.64	-11.8974±0.12	2.6620±0.39	-3.7092±0.09	5.8519±0.55
	28	3.0429±0.59	-21.3261±0.19	2.4305±0.37	-4.6054±0.32	5.9943±0.24
**IND9**						
S_l, %	7	-6.3229±0.31	5.2141±0.19	0.5620±0.36	1.9410±0.09	0.1124±0.23
	14	-6.5341±0.17	11.2776±0.28	1.2176±0.21	2.2772±0.34	0.2435±0.99
	21	-6.3850±0.98	16.8689±0.33	2.4354±0.08	3.8316±0.46	0.4870±0.24
	28	-6.2608±0.87	26.1479±0.18	2.9976±0.39	4.7390±0.61	0.5995±0.11
S_p, %	7	3.6738±0.45	4.9940±0.88	3.1835±0.25	0.0726±0.71	4.5150±0.29
	14	4.2857±0.43	5.1868±0.79	3.5673±0.67	0.0753±0.24	5.3625±0.33
	21	4.3769±0.38	5.4909±0.13	3.8084±0.28	0.0785±0.32	5.5098±0.47
	28	4.4245±0.28	5.8252±0.78	3.8628±0.37	0.0793±0.45	5.5978±0.58
D_m, %	7	3.4782±0.21	-3.3071±0.99	2.9133±0.24	3.3425±0.39	5.0268±0.27
	14	3.3043±0.17	-6.4239±0.66	2.9079±0.38	0.3634±0.27	5.3727±0.38
	21	3.2546±0.31	-12.0390±0.70	2.6932±0.21	-3.7351±0.44	5.6005±0.46
	28	3.2223±0.25	-20.3901±0.24	2.6299±0.29	-3.7400±0.26	5.6484±0.55
**IND10**						
S_l, %	7	-6.7270±0.15	6.2167±0.23	0.5686±0.21	1.9657±0.33	0.1137±0.24
	14	-6.6163±0.26	11.4134±0.27	1.2322±0.14	2.2808±0.56	0.2464±0.18
	21	-6.4654±0.38	17.0719±0.25	2.4644±0.09	3.8805±0.49	0.4929±0.10
	28	-6.3398±0.21	26.4627±0.39	3.0330±0.26	4.7995±0.35	0.6066±0.23
S_p, %	7	3.7181±0.23	5.0288±0.43	3.1974±0.38	0.0710±0.53	4.5676±0.87
	14	4.3340±0.36	5.2536±0.39	3.6083±0.42	0.0763±0.40	5.4455±0.98
	21	4.4263±0.29	5.5663±0.45	3.8523±0.28	0.0796±0.61	5.5950±1.01
	28	4.4745±0.33	5.9149±0.23	3.9075±0.54	0.0803±0.23	5.6842±0.19
D_m, %	7	3.4782±0.25	3.3071±0.35	3.9133±0.50	3.1762±0.35	5.5268±0.36

	14	3.3043±0.10	-6.4239±0.26	3.7679±0.79	0.3529±0.23	5.6727±0.29
	21	3.2546±0.85	-12.0390±0.38	3.6932±0.23	-3.7559±0.36	5.7505±0.34
	28	3.2223±0.26	-20.3901±0.46	3.6299±0.56	-3.4030±0.19	5.8284±0.28
				**CQ**		
S_l, %	7	0.1902±0.20	4.5901±0.21	0.5714±0.33	2.8500±0.19	0.2025±0.31
	14	0.2731±0.37	13.3305±0.20	1.3100±0.56	4.8202±0.20	0.2531±0.25
	21	0.5200±0.46	19.8267±0.31	1.8622±0.48	5.8704±0.56	0.3037±0.18
	28	0.7601±0.09	26.4529±1.09	2.3905±0.56	7.5622±0.54	0.3037±0.11
S_p, %	7	3.7405±0.31	4.0234±0.28	4.1871±0.67	0.0949±0.52	5.2303±0.10
	14	3.7432±0.27	5.4545±0.21	4.2870±0.85	0.0963±0.30	5.5835±0.02
	21	4.0025±0.23	5.6470±0.65	4.3177±0.92	0.0951±0.19	5.7666±0.26
	28	4.0923±0.43	6.0618±0.42	4.3832±0.75	0.1054±0.11	5.8345±0.58
D_m, %	7	3.5929±0.44	4.1927±0.31	2.9001±0.65	0.0902±0.05	5.5189±0.33
	14	3.5945±0.51	-8.5239±0.87	2.2102±0.45	-4.7300±0.21	5.6455±0.45
	21	3.6239±0.63	-21.5422±0.73	1.8106±0.38	-5.7700±0.38	5.7974±0.51
	28	3.4656±0.71	-26.2820±0.64	1.5603±0.23	-7. 4705±0.24	5.8734±0.19

distilled water

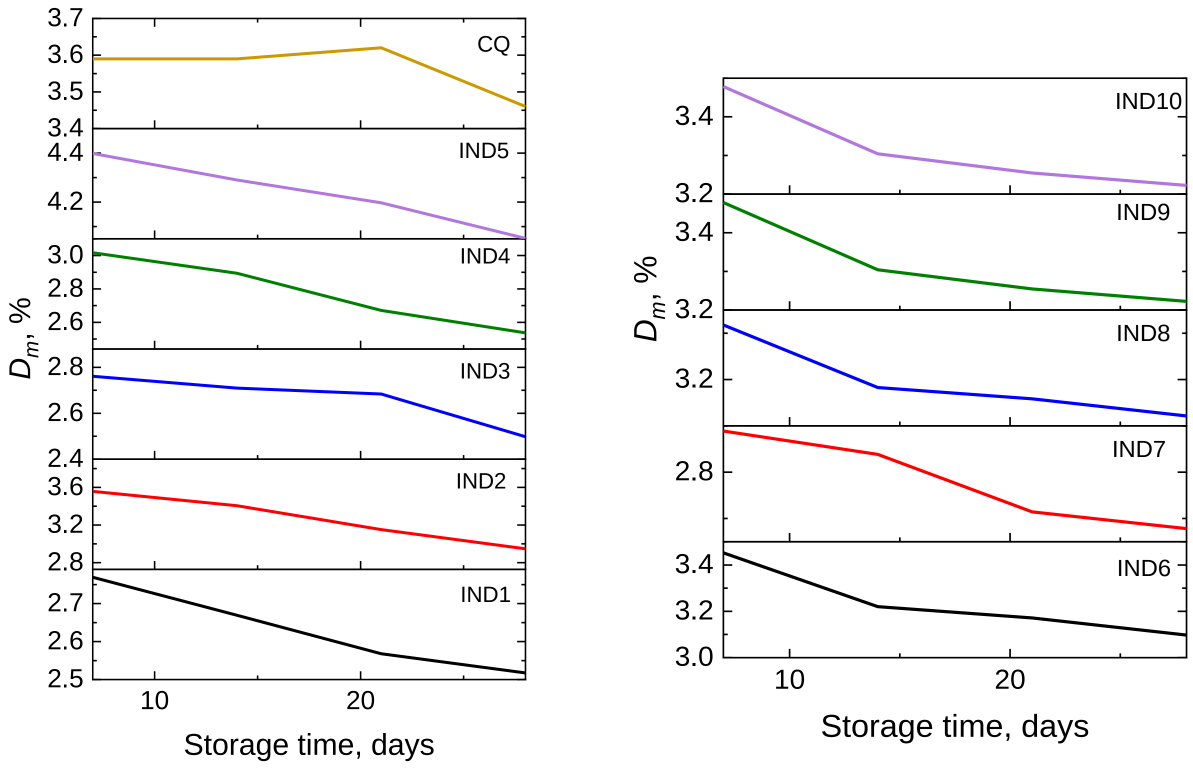

artificial saliva

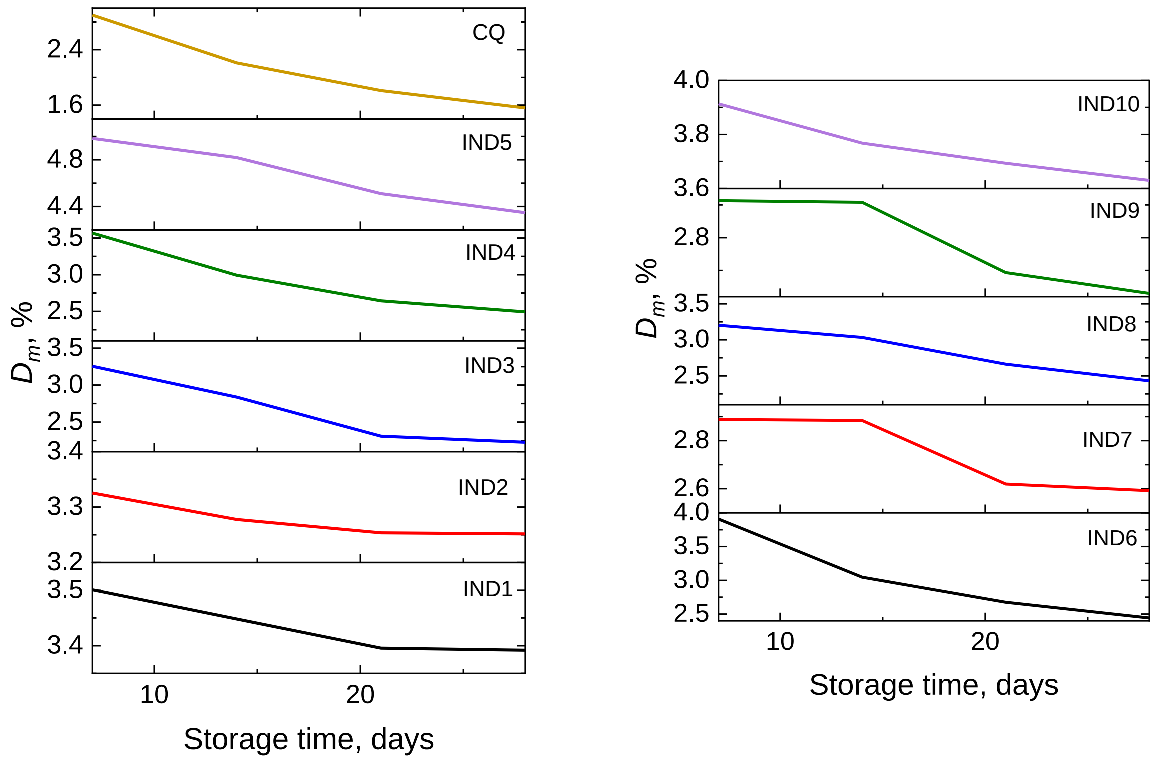

3% acetic acid

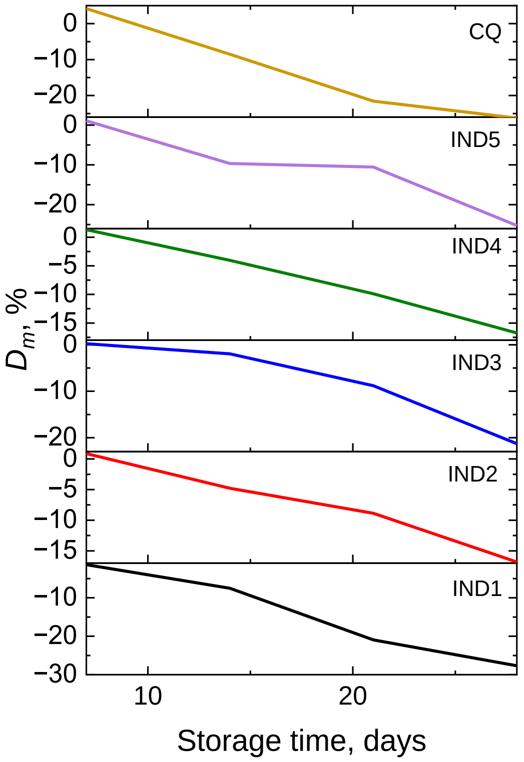

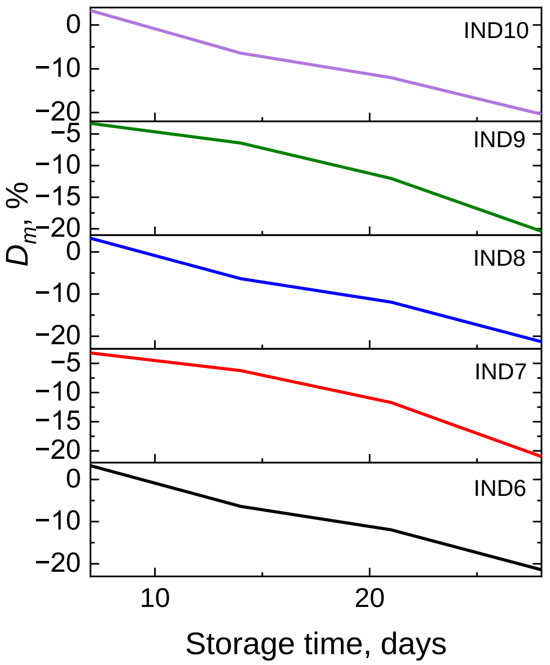

***n*-heptane**

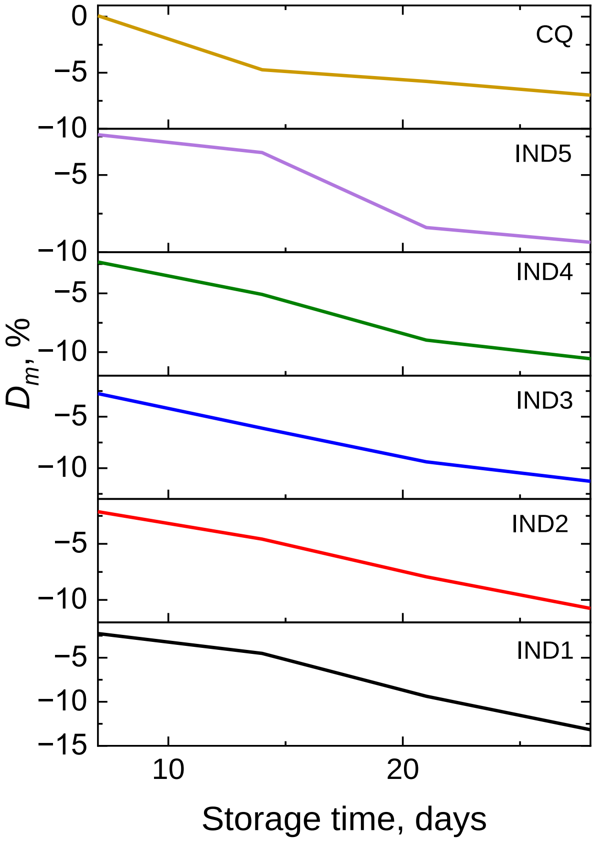

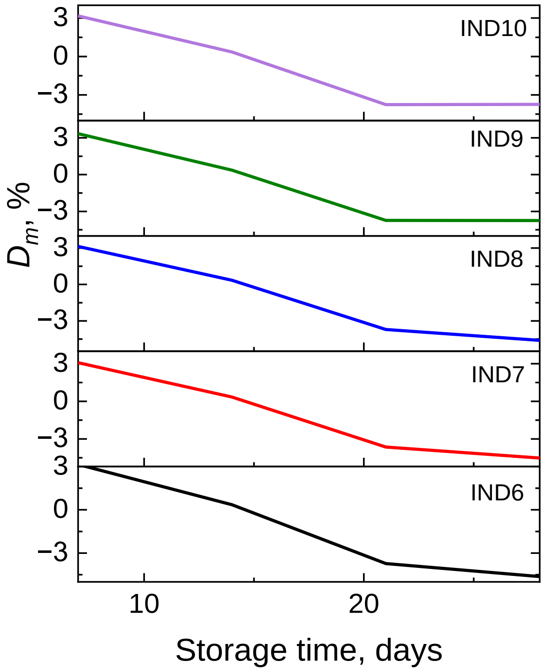

coffee

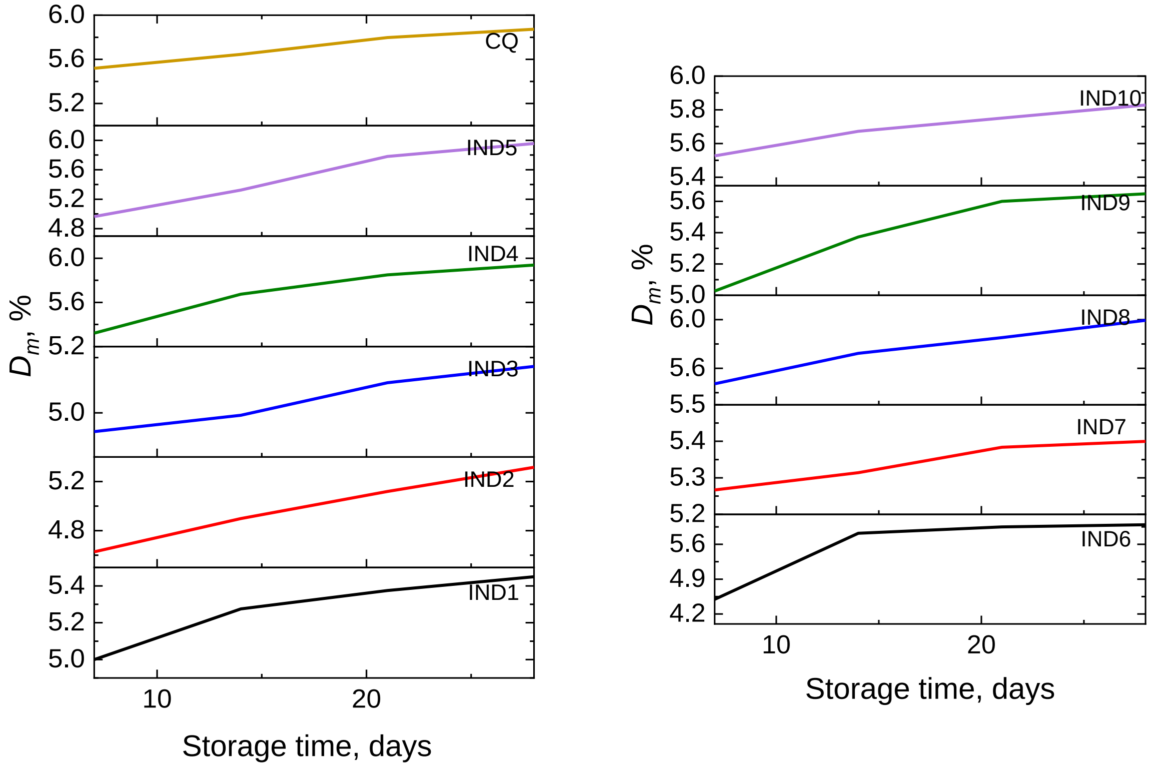

Figure S2. Dependence of the mean values of mass change of the tested materials on the conditioning time in selected solutions simulating the oral cavity environment.
